# Artificial Intelligence in Electrochemical Sensing: A Network Evidence Map of Translational Barriers and Pathways to Point-of-Care Deployment

**DOI:** 10.3390/bios16090470

**Published:** 2026-08-27

**Authors:** Muhammad Saqib, Elena I. Korotkova, Kunquan Li, Neda Firoz, Mrinal Vashisth, Amrit L. Hui, Olga I. Lipskikh, Pradip Kumar Kar

**Affiliations:** 1 Chemical Engineering Division, School of Earth Sciences and Engineering, National Research Tomsk Polytechnic University, 30 Lenin Avenue, 634050 Tomsk, Russia; eikor@tpu.ru (E.I.K.);; 2 UNESCO Laboratory of Environmental Electrochemistry, Department of Analytical Chemistry, Faculty of Science, Charles University, Hlavova 8/2030, CZ 128 43 Prague, Czech Republic; 3 College of Engineering, Nanjing Agricultural University, Nanjing 210031, China; kqlee@njau.edu.cn; 4 National Research Tomsk State University, 36 Lenin Avenue, 634050 Tomsk, Russia; 5 Parasitology Laboratory, Department of Zoology, Cooch Behar Panchanan Barma University, Vivekananda Street, Cooch Behar 736101, India; karpradip@gmail.com

**Keywords:** machine learning (ML), artificial intelligence (AI), electrochemical biosensor, electrochemical sensor, voltammertic method, network evidence map, explainable AI, point-of-care diagnostics

## Abstract

The integration of artificial intelligence (AI) and machine learning (ML) with electrochemical sensing has revolutionized analytical diagnostics by overcoming traditional limitations such as signal drift, peak overlapping, and matrix interference. However, despite the exponential growth of this field, a unified framework evaluating translational feasibility remains absent. This review critically analyzes AI/ML architectures applied to electrochemical sensors and biosensors from 2016 to 2025. To the best of our knowledge, this work introduces the first coded Network Evidence Map to quantitatively map the co-occurrence of methodological strengths, weaknesses, and translational barriers across the examined literature. The analysis reveals that while deep learning and ensemble models excel in signal deconvolution and multiplexing, the field is severely constrained by systemic bottlenecks. Network pathways demonstrate that over 83% of studies lack uncertainty quantification, and data scarcity coupled with restricted data-sharing policies critically undermines model reproducibility. Furthermore, batch-to-batch hardware variability measurably co-occurs with the opacity of black-box algorithms, hindering regulatory approval. We conclude that advancing from laboratory proof-of-concept to real-world point-of-care deployment necessitates a paradigm shift toward open-source electrochemical repositories, explainable AI (XAI), physics-informed machine learning, and hardware-software co-design.

## 1. Introduction

The integration of AI and ML with electrochemical sensing platforms has dramatically changed diagnostic and environmental monitoring [1,2]. The combination of real-time data acquisition and predictive analytics enables AI sensors to overcome traditional challenges in electrochemistry such as electrode fouling, signal drift, and interference from complex matrices [3,4]. The computational frameworks bridge the gap between raw electrochemical signals and actionable clinical and environmental insights, thus enabling adaptive calibration and automated decision-making [5,6]. Moreover, AI algorithms are enhancing sensor optimization through model-driven material discovery and performance prediction, replacing trial-and-error protocols [7]. Therefore, today’s AI-integrated electrochemical biosensors provide high-throughput screening, multiplexed detection of analytes, and scalability for point-of-care (POC) and Internet-of-Things (IoT) networks [8].

Electrochemical platforms remain highly popular for POC applications due to their low fabrication cost, miniaturization potential, and compatibility with flexible electronics [9,10,11]. For the last decade, researchers have systematically incorporated nanomaterials to expand electroactive surface areas and improve electron transfer kinetics [12,13]. Graphene and its derivatives stand out among other carbon-based architectures due to their exceptional electrical conductivity, tunable surface chemistry and high surface-to-volume ratios [14,15,16]. Enhanced signal-to-noise ratio, reduced degradation of sensitivity, and stable immobilization of biorecognition elements are achieved through graphene-based architectures [17,18]. Researchers continue to integrate graphene-based electrodes with flexible sensor printing and low-power microcontrollers to translate the advantages of materials into practical devices [14,19]. The convergence of engineering disciplines yields wearable, comfortable platforms capable of continuous physiological monitoring while maintaining mechanical durability and electrical reliability [20].

Although there has been significant progress in material modifications, the translation of nanomaterial-based electrochemical sensors and biosensors from controlled laboratory conditions to the complex real-world environment remains a critical bottleneck [21]. Biological and environmental samples introduce severe matrix effects including protein adsorption, biofouling, and overlapping redox signatures that obstruct target analyte signals [6,22,23,24,25]. Linear assumptions used in conventional calibration strategies fail to capture non-linear signal dynamics in multiplexed or dynamic settings [26]. To address these limitations, researchers are using an increasing number of ML architectures such as Artificial Neural Networks (ANNs), Convolutional Neural Networks (CNNs), and tree-based ensemble frameworks to decode multivariate voltammetric, amperometric and impedance data [27]. The algorithms identify hidden feature patterns, compensate for drift and enable robust quantification of multiple analytes without extensive sample pretreatment [28].

Although the field of AI/ML electrochemical biosensors has expanded exponentially within the last ten years, with most of the recent literature published during this time frame, comprehensive synthesis remains fragmented. Previous review papers focus either on the synthesis of nanomaterials [28] or on the isolated algorithmic performance [4,8], leaving a critical gap in understanding the interaction between material design, computational architecture, and deployment constraints interact. Few studies evaluate data transparency, uncertainty quantification (UQ), edge computing readiness, and standardized validation protocols for real-world use. As a result, the field lacks a unified evidence-based framework linking sensor architecture with AI robustness and translational feasibility.

The novelty of this critical review lies in the introduction of a quantitative 26-code Network Evidence Map; the first systematic, coded evidence synthesis in this domain that maps not only individual study attributes but the joint-probability co-occurrence and sequential pathways of strengths, weaknesses, and translational barriers. Unlike prior reviews that catalogue algorithmic performance in isolation, this work adopts a consistently critical lens, interrogating not only the achievements of AI/ML models in controlled settings, but also the underlying reasons many reported systems remain confined to the laboratory bench. This dual emphasis on capability and translational feasibility is intended to serve both newcomers entering the field and experienced researchers seeking a realistic appraisal of deployment readiness.

**Figure 1:**
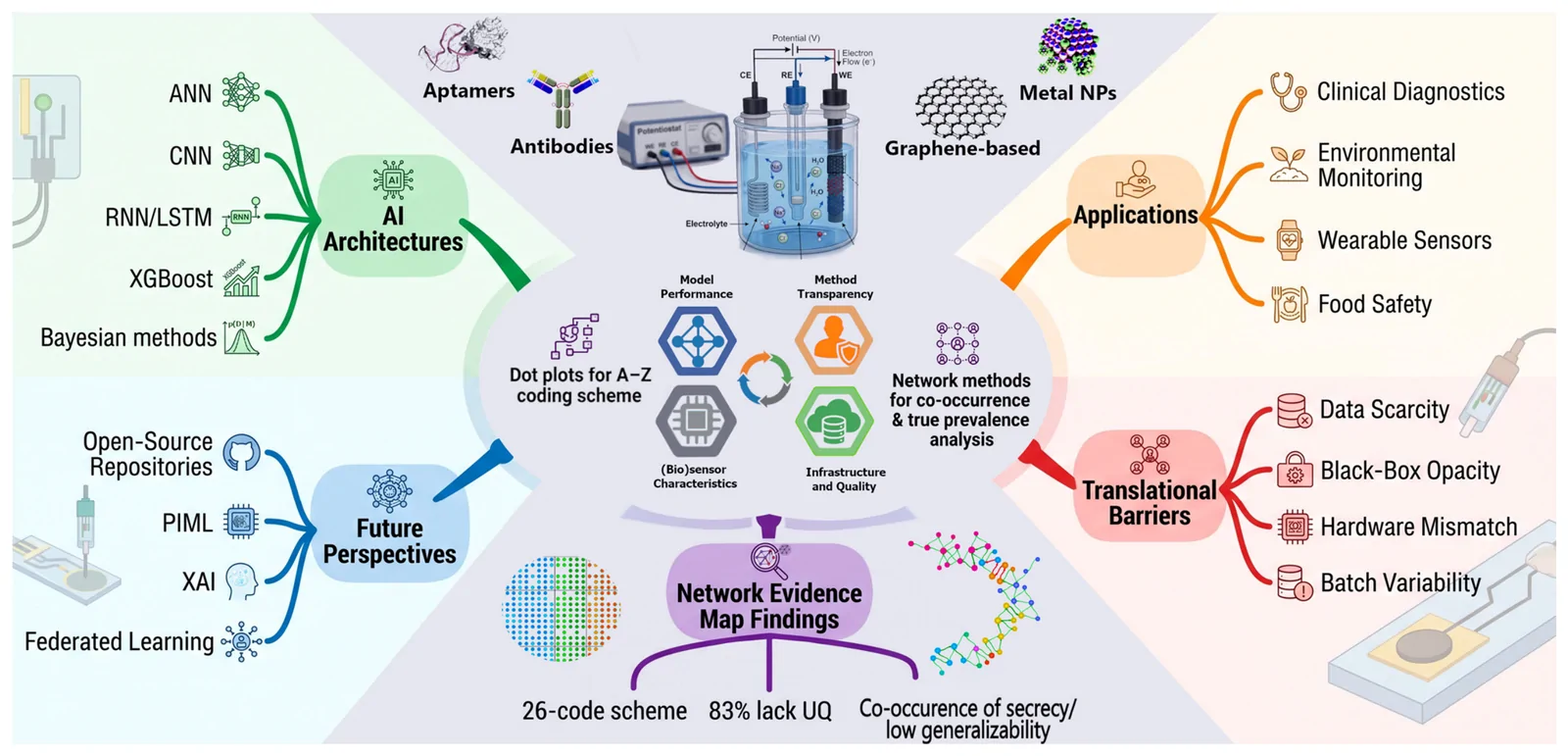
Schematic overview of AI/ML-driven electrochemical sensing: architectures, applications, translational barriers, and future perspectives.

This critical review addresses these gaps by systematically assessing the development of electrochemical sensors/biosensors and their associated algorithmic architectures over the past decade across clinical, environmental, and wearable applications. We map the co-evolution of electrochemical sensing platforms, algorithmic architectures, and real-world deployment barriers to identify pathways toward next-generation smart sensors. To ensure comprehensive coverage and reproducibility, the systematic literature search was conducted covering the time from 1 January 2016 to 30 December 2025. The Scopus database was queried using the following advanced search string: first (‘electrochemical sensor’ OR ‘electrochemical biosensors’ OR ‘electrochemical detection’ OR ‘electroanalytical methods’) AND second (‘artificial intelligence’ OR ‘AI’ OR ‘machine learning’ OR ‘ML’). This initial query yielded 195 records. After removal of review papers (5 removed), 190 records remained for title/abstract screening. Following application of inclusion criteria (peer-reviewed research articles reporting original AI/ML integration with electrochemical sensing platforms; English language; full-text accessible), 50 records were excluded for not satisfying the inclusion criteria (no AI/ML integration or use of an electrochemical method). Full-text assessment of 140 articles led to further exclusion of 7 studies, finally resulting in 133 research papers. The flow diagram is provided as Supplementary Figure S1. Figure 1 illustrates the critical review of AI/ML-driven electrochemical sensing, showing the integration of various AI architectures into the central electrochemical platform to enable key applications in clinical diagnostics, environmental monitoring, wearables, and food safety. However, widespread translation is hindered by systemic barriers. Furthermore, novel Network Evidence Map findings highlight the co-occurrence of restricted data access with low generalizability, leading to proposed future perspectives for overcoming these challenges.

This review is organized as follows: Section 2 focuses on the primary AI/ML architectures applied in electrochemical sensors and biosensors, including standard feedforward ANNs, CNNs, RNNs/LSTMs, hybrid models, and specialized DNNs. Section 3 examines Support Vector Machines (SVMs) and their variants for diagnostic screening and quantitative analysis. Section 4 discusses tree-based models and ensemble methods, such as Decision Trees, Random Forests, and Gradient Boosting algorithms. Section 5 covers linear and multivariate regression models, including PLS and PCR. Section 6 explores Bayesian and probabilistic methods for uncertainty-aware modeling. Section 7 highlights other specialized algorithms, including instance-based, unsupervised, and symbolic regression methods. Section 8 presents a novel Network Evidence Map, utilizing a 26-code scheme to quantitatively analyze the co-occurrence of methodological strengths, weaknesses, and translational barriers across the reviewed literature. Section 9 critically synthesizes the major translational barriers, including data scarcity, the “real-world” gap, black-box opacity, hardware–software mismatches, and batch-to-batch variability. Section 10 outlines future perspectives, emphasizing open-source repositories, XAI, physics-informed machine learning, and edge-AI co-design. Finally, Section 11 concludes the review by summarizing key findings and defining the prerequisites for transitioning from laboratory proof-of-concept to robust, real-world diagnostic ecosystems.

## 2. AI/ML Architectures in Electrochemical Sensing

**Figure 2:**
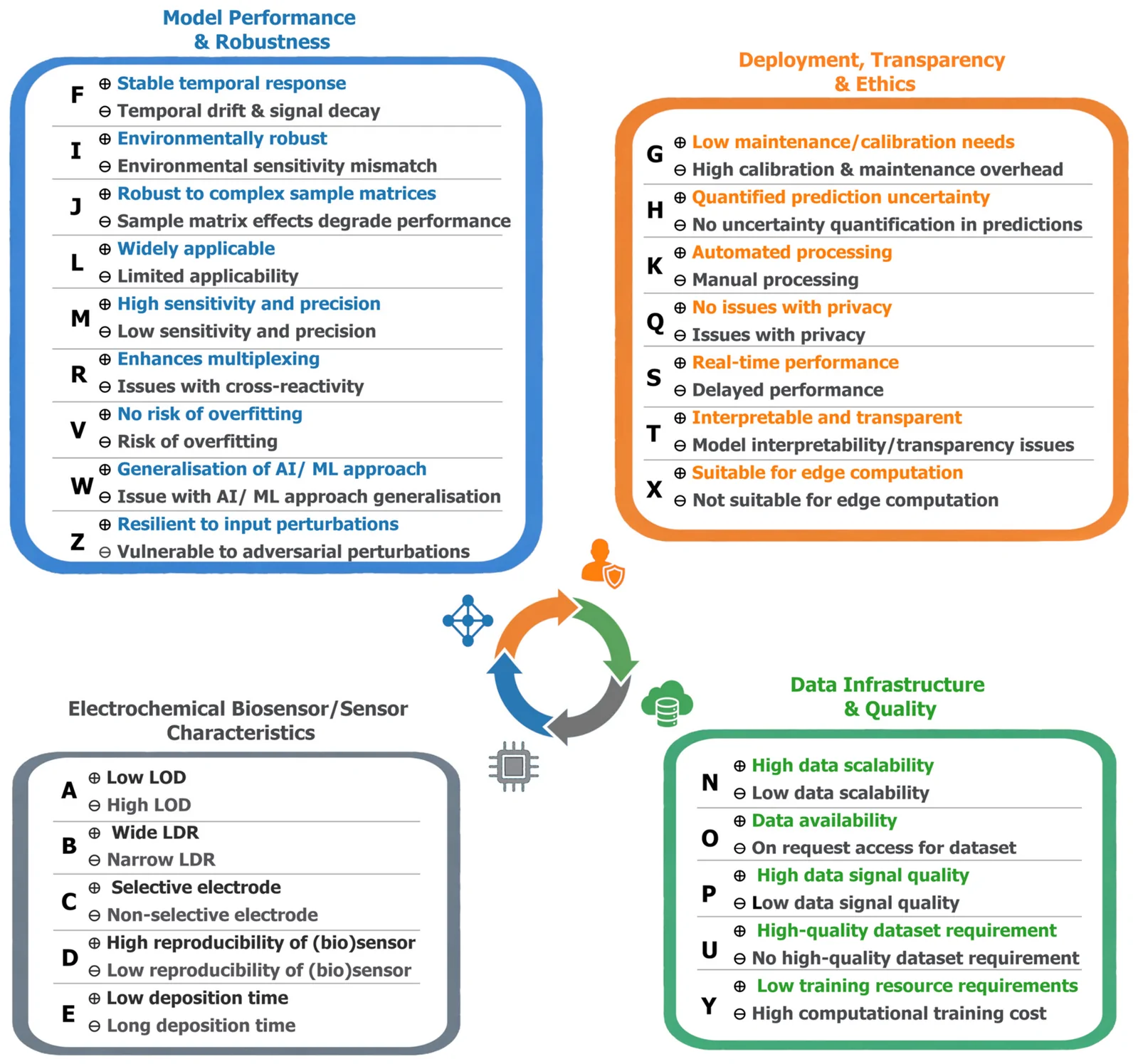
Schematic representation of the 26-code classification scheme used for the Network Evidence Map, organized into four thematic domains: Electrochemical Biosensor/Sensor Characteristics (Codes A–E, gray), AI/ML Model Performance & Robustness (Codes F, I, J, L, M, R, V, W, Z, blue), Deployment, Transparency & Ethics (Codes G, H, K, Q, S, T, X, orange), and Data Infrastructure & Quality (Codes N, O, P, U, Y, green). Within each domain, codes are listed alphabetically, and each code is defined by a positive (⊕, strength) and a negative (⊖, weakness) pole. The central clockwise arrows indicate the recommended reading order starting from the sensor/hardware domain (**bottom left**) and proceeding clockwise through model performance (**top left**), deployment/transparency/ethics (**top right**), and data infrastructure (**bottom right**), and the mutual interdependence of the four domains. Codes are standardized to allow quantitative co-occurrence analysis across the reviewed studies (2016–2025).

**Figure 3:**
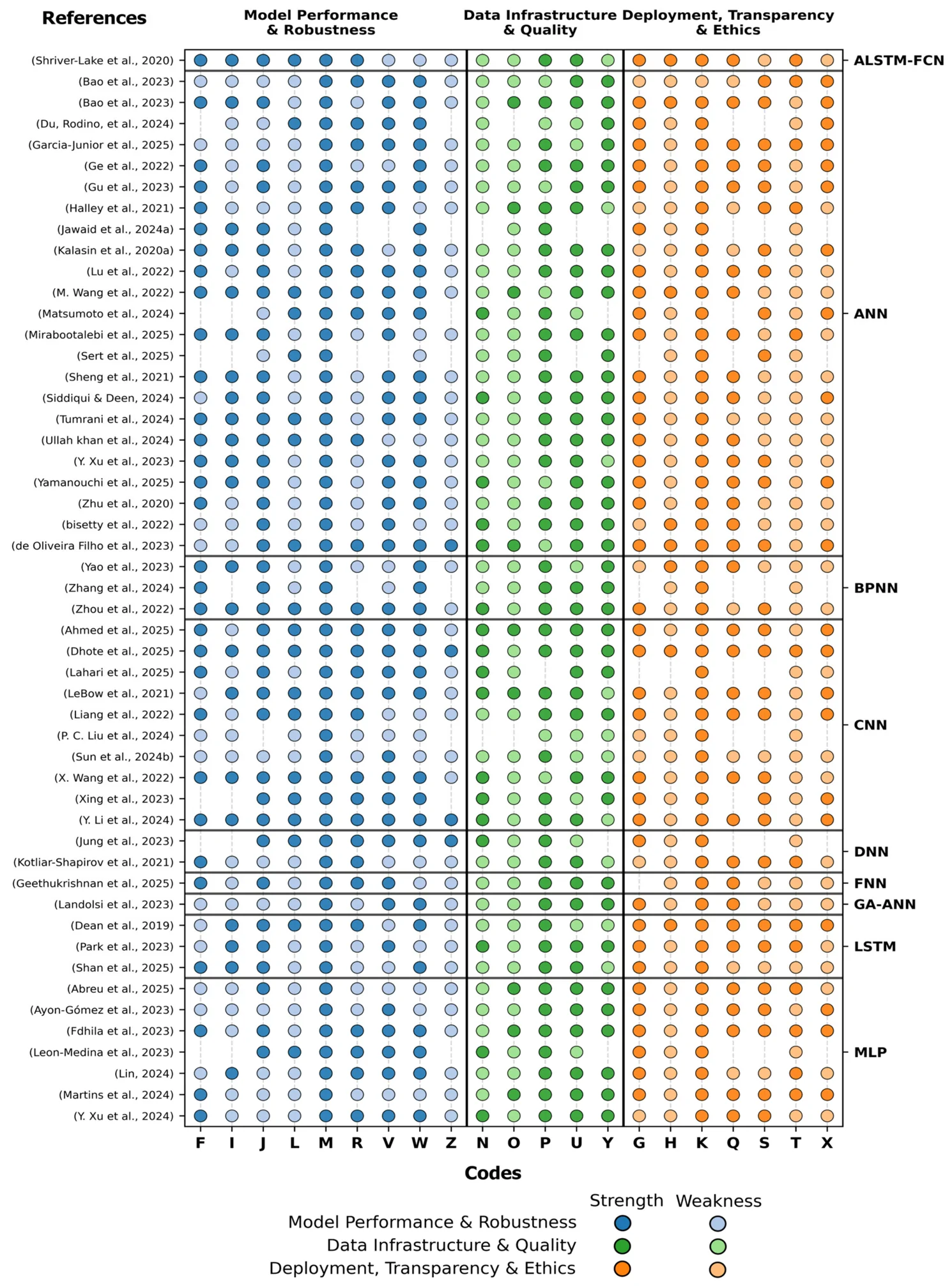
Dot-plot summary of strengths and weaknesses for Artificial Neural Network (ANN) and variant paradigms (including CNN, RNN/LSTM, hybrid, and DNNs) in electrochemical sensing (2016–2025). Dark colors indicate observed strengths; light colors indicate weaknesses. Absent cells indicate data not reported or inferred. Note the high frequency of “black-box” opacity and limited data scalability across deep learning architectures (Refs. [29,30,31,32,33,34,35,36,37,38,39,40,41,42,43,44,45,46,47,48,49,50,51,52,53,54,55,56,57,58,59,60,61,62,63,64,65,66,67,68,69,70,71,72,73,74,75,76,77,78,79]).

**Figure 4:**
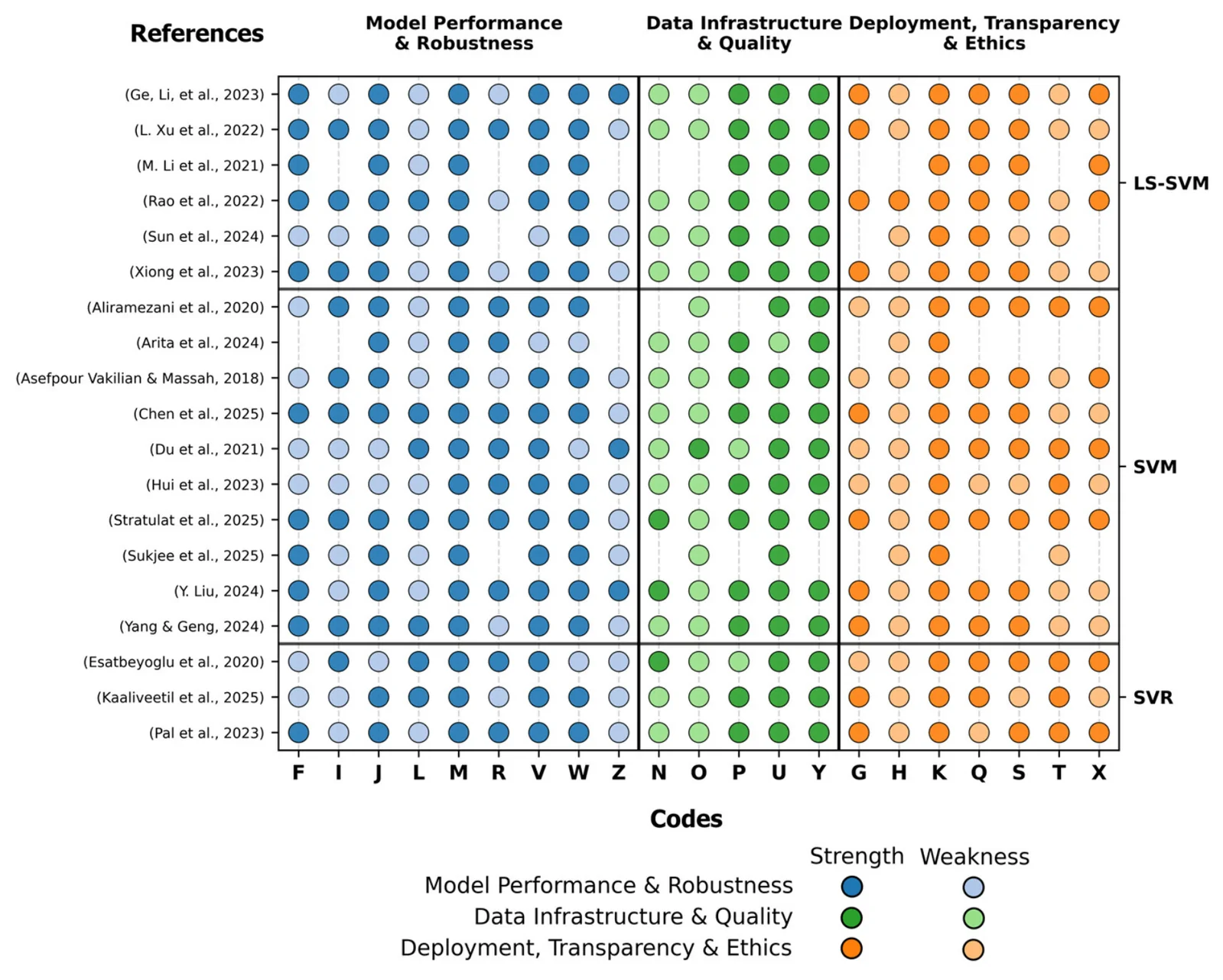
Dot-plot summary of strengths and weaknesses for SVM and variant implementations (2016–2025). The plot highlights the trade-off between computational efficiency in small datasets and the lack of probabilistic output (uncertainty quantification) (Refs. [80,81,82,83,84,85,86,87,89,90,91,92,93,94,95,96,97,98,99]).

**Figure 5:**
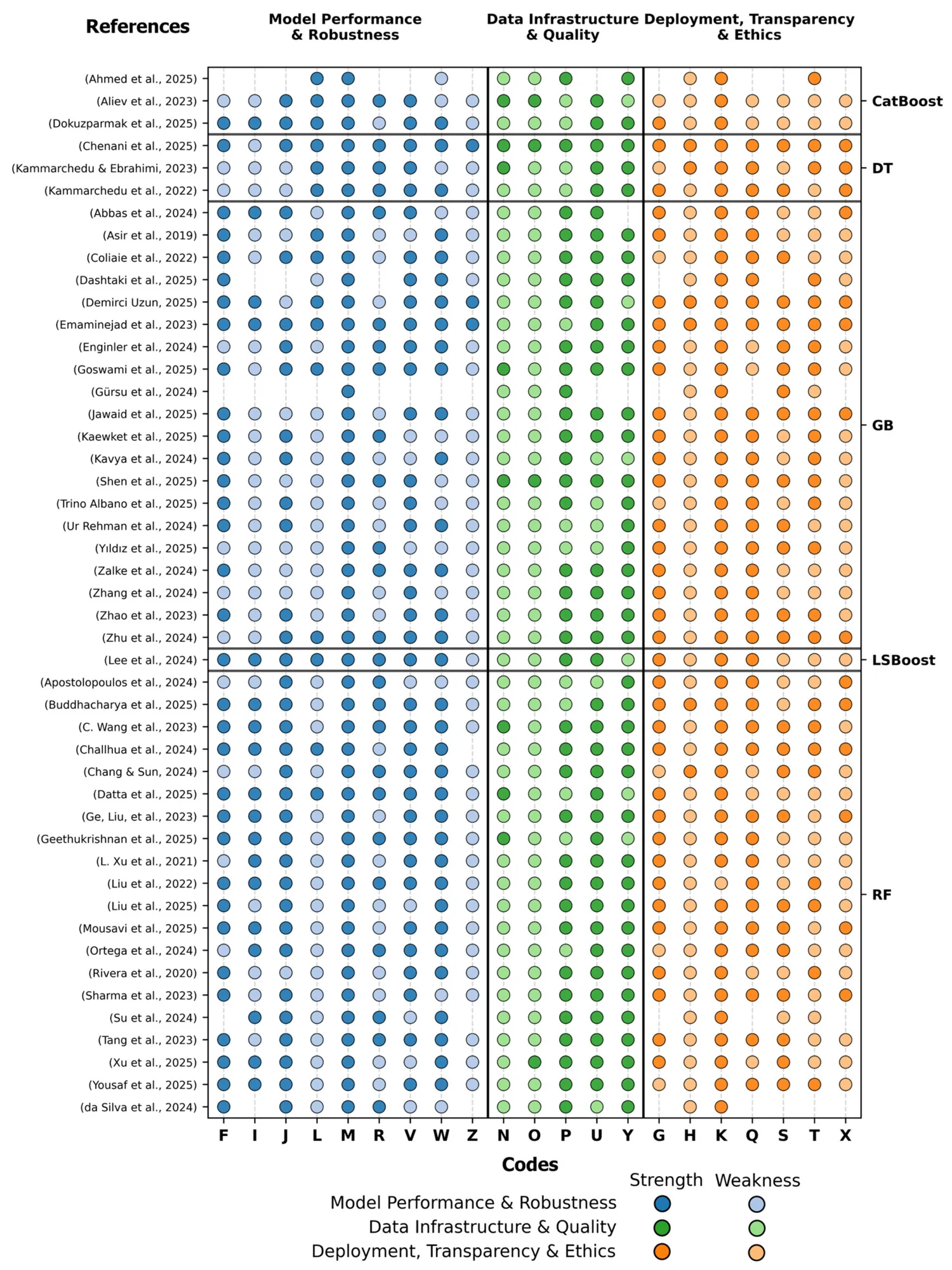
Dot-plot summary of strengths and weaknesses for Tree-based and ensemble paradigms (Decision Trees, Random Forest, Gradient Boosting) (2016–2025). The visualization underscores the correlation between high predictive performance and increased computational resource requirements, posing challenges for edge deployment (Refs. [88,100,101,102,103,104,105,106,107,108,109,110,111,112,113,114,115,116,117,118,119,120,121,122,123,124,125,126,127,128,129,130,131,132,133,134,135,136,137,138,139,140,142,143,144]).

**Figure 6:**
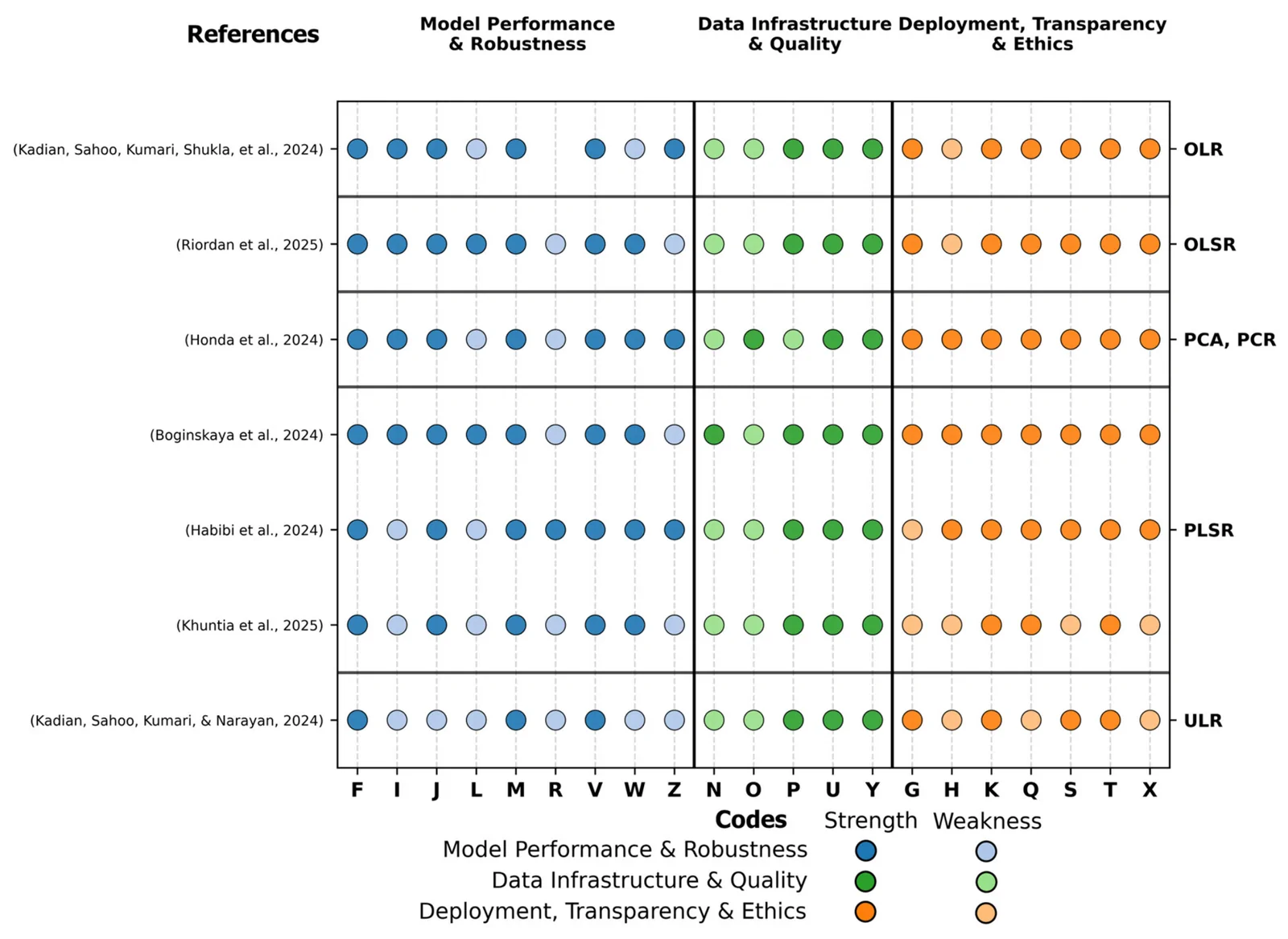
Dot-plot summary of strengths and weaknesses for linear and multivariate regression models (2016–2025). The plot highlights the high interpretability and low computational cost of these methods, contrasted with their limitations in handling non-linear signal drift and complex matrix interferences (Refs. [146,147,148,149,150,151,152]).

**Figure 7:**
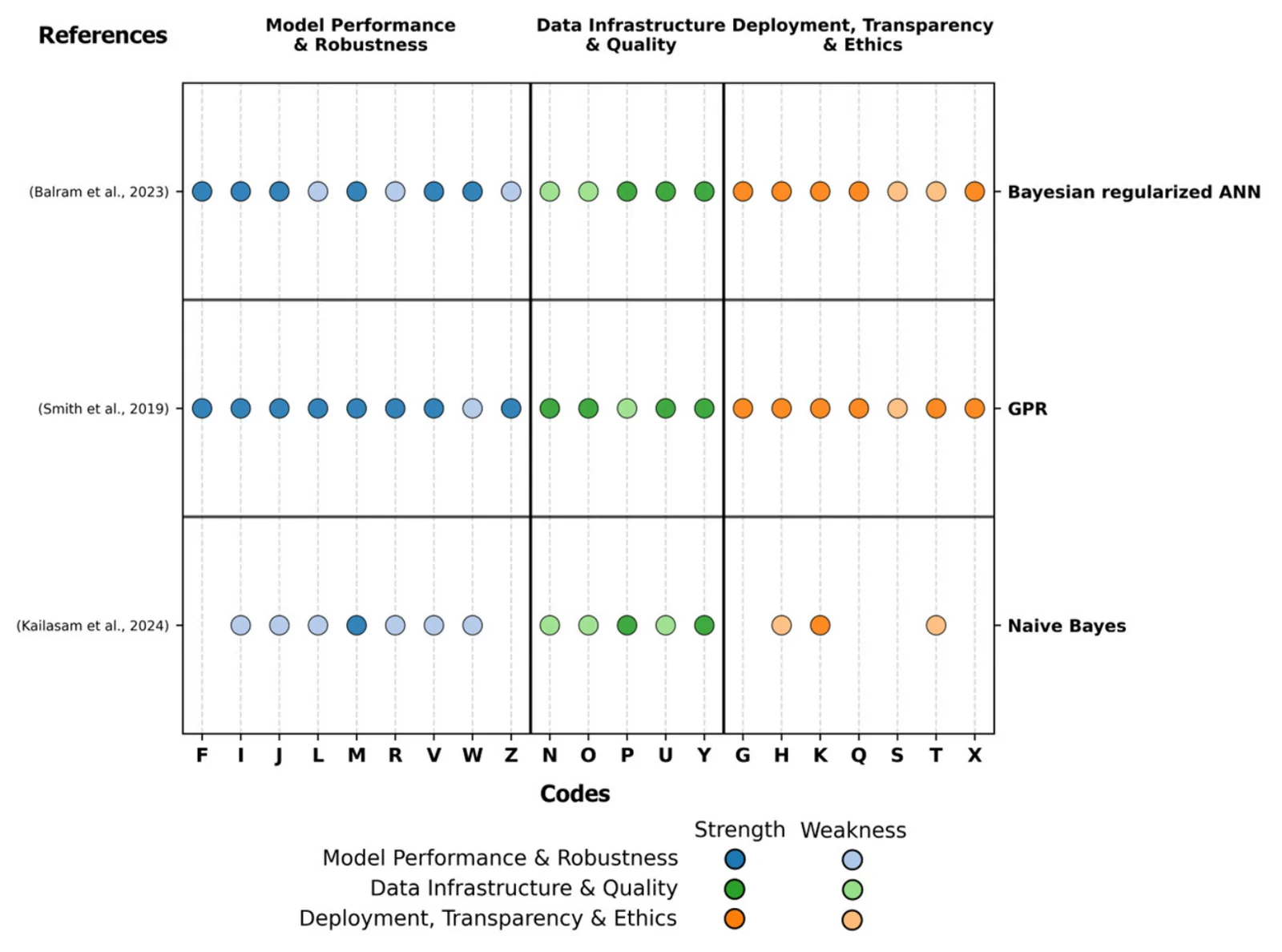
Dot-plot summary of strengths and weaknesses for Bayesian and probabilistic approaches (2016–2025). While these methods excel in uncertainty quantification, the plot indicates significant barriers related to computational scalability and integration with low-power electrochemical hardware (Refs. [153,154,155]).

**Figure 8:**
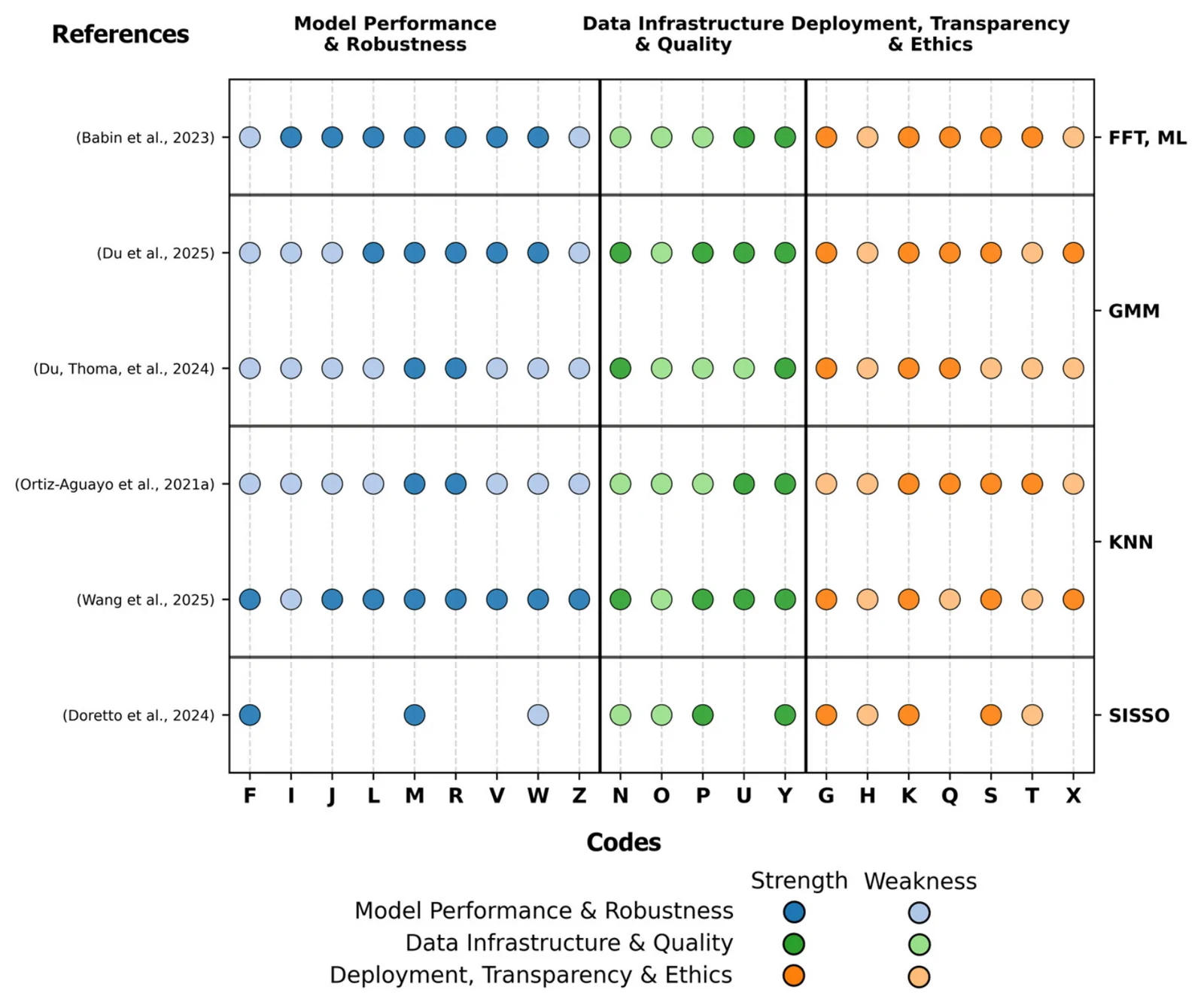
Dot-plot summary of strengths and weaknesses for “Other” algorithmic paradigms, including instance-based (KNN), unsupervised generative models (GMM, k-Means), symbolic regression (SISSO), and custom signal processing (FFT) (2016–2025). Dark colors indicate observed strengths; light colors indicate weaknesses. Absent cells indicate data not reported or inferred. The plot highlights the trade-off between computational efficiency in edge devices and the challenges of generalizability and regulatory standardization (Refs. [156,157,158,159,160,161]).

The potential to utilize AI and ML to enhance the capabilities of electrochemical biosensors is driven by the fact that electrochemical signal responses are inherently complex and exhibit non-linear response curves; overlapping redox peaks; temporal drift; and matrix-dependent interferences. These characteristics often render traditional univariate calibrations ineffective, necessitating the use of a variety of algorithmic structures. This section provides an introductory overview of the primary AI/ML architecture families discussed herein, covering their theoretical basis, applicability to electrochemical data, and representative applications. To systematically evaluate translational feasibility, we developed a 26-code Network Evidence Map scheme (detailed in Figure 2). The 26-code scheme was developed iteratively, validated by a subject-matter expert, and coded by a single trained coder across two rounds with near-perfect intra-coder reproducibility (Jaccard Similarity: 100%/99.8%) (Supplementary Section S1). The complete codebook with decision rules is provided in Supplementary Table S1. This coding framework categorizes electrochemical characteristics, AI model attributes, and translational barriers, which are visually summarized in the subsequent dot-plots (Figure 3, Figure 4, Figure 5, Figure 6, Figure 7 and Figure 8).

### 2.1. Standard and Feedforward Neural Networks

ANNs, specifically multilayer perceptron (MLP) feedforward architectures such as back propagation ANNs (BP-ANN) and single-layer perceptrons, continue to be the most popular machine learning approaches used in electrochemical biosensors. This is largely because these architectures can simulate non-linear interactions between a variety of voltammetric characteristics and analyte concentration, making such networks particularly suitable for resolving complex electrochemical responses where traditional univariate calibrations fail. Recent studies have demonstrated that feedforward ANNs perform four related functions, including (i) disentangling overlapping signals in multiplexed multi-analyte mixtures, (ii) correcting sensor drift and changes due to environmental factors, (iii) enabling POC edge deployable diagnostics, and (iv) providing data for an electronic tongue (e-tongue).

#### 2.1.1. Resolving Signal Overlap and Enhancing Computational Selectivity

We first consider feedforward ANNs for decoupling overlapped redox peaks in complex biological/environmental matrices, because conventional electrochemistry has long suffered from cross-reactivity; ANNs can overcome this limitation by identifying multivariate feature patterns from raw or preprocessed voltammograms. For example, radial basis function (RBF) networks and back propagation Artificial Neural Network have been used to identify palmitic acid signals in food matrices based on current responses obtained using differential pulse voltammetry (DPV) [29], whereas a feedforward ANN was shown to reduce metronidazole interference in chloramphenicol detection by utilizing features that correlate with Pearson’s correlation coefficients from both voltammetric and amperometric measurements [30]. Similar to these examples, ANNs were also successful in quantifying paracetamol, ascorbic acid and uric acid in pharmaceutical formulations [31], as well as caffeine and chlorogenic acid in coffee samples where the prediction accuracy increased from approximately 30% to greater than 90% [32]. The use of shallow ANNs in neurochemical sensing has also played an important role in discriminating Dopamine from ascorbic and uric acid in serum and cerebrospinal fluids, resulting in >96% classification accuracy even when the sample was contaminated [33]. While there has been progress made in reducing cross-reactivity, it remains clear that computational selectivity cannot replace optimized surface chemistry or molecular imprinting [30,34].

#### 2.1.2. Modeling Sensor Degradation, Drift, and Environmental Variability

Furthermore, beyond the ability to quantify an analytical compound, ANNs are being used increasingly as virtual calibration tools to create models of sensor age, hysteresis, and cross-sensitivity with regard to environmental factors. Low-cost and biodegradable electrochemical sensor platforms are highly susceptible to temporal signal decay. As such, using longitudinal datasets, ANNs can be trained to predict and correct this type of signal decay. An example is training a two-hidden-layer ANN on 9385 chronoamperograms (CA) generated from free chlorine sensors operated under various pH levels and time points to achieve a Pearson correlation coefficient of 0.9950 [35]. Later, a much larger feedforward network (five layers) was developed to continuously generate predicted values of free chlorine concentration based upon the raw linear sweep voltammetry (LSV) curve and adjust for pH shift and electrode drift [36]. Additionally, MLPs were incorporated into electrochemical impedance spectroscopy (EIS) data to develop predictive models of glucose concentration that accounted for changes in reactance [37]. Finally, ANN architectures have also been developed to predict the non-linear responses of tyrosine sensors in response to variations in pH and temperature [38,39]. In total, these studies demonstrated significant reductions in labor required for manual recalibration of sensor platforms. However, it has been observed that models trained on buffer systems may lose accuracy when tested in real biological fluids due to differences in ionic strength, protein fouling, and batch-to-batch variation [39,40].

#### 2.1.3. Edge Deployment and Real-Time Point-of-Care Systems

The relative simplicity and computational efficiency of shallow feedforward networks have enabled direct deployment on low-power microcontrollers, facilitating real-time, standalone POC testing without cloud connectivity. As such, many successful demonstrations of embedding ANNs/MLPs onto ESP32 system-on-chips (SoCs) and Raspberry Pi have been reported for on-device inference. Examples of such successful implementations include: a non-enzymatic glucose sensor implemented using ANN-MLPs for inference directly on a microcontroller (i.e., ESP32) operated at fixed potentials, achieving an accuracy of 97.8% [41]; a Dopamine biosensor developed using TinyML, for wearable neurochemical screening with quantized 8-bit neural networks [33]; and a satellite linked textile-based sweat sensor for real-time detection of creatine levels and heat stress [42]. Additionally, an MLP regressor has been utilized in combination with a Decision Tree to predict glucose concentrations in diluted serum without requiring traditional calibration curves, thereby streamlining the diagnostic workflow for rapid POC testing [43]. Although the ability to deploy AI at the “edge” greatly reduces latency and increases mobility, this capability is often achieved through model quantization, resulting in a loss of approximately 2–4% in accuracy. While this margin may be acceptable for screening applications (e.g., glucose monitoring), it may exceed clinical tolerance limits for low-abundance biomarker detection, necessitating rigorous validation against gold-standard methods. Most current embedded systems also lack sufficient memory bandwidth to perform high-dimensional raw voltammetry processing; therefore, aggressive feature extraction is required before performing inference [33,43]. While these demonstrations prove technical feasibility, such implementations remain isolated case studies rather than standardized commercial solutions, as detailed in the translational barrier analysis in Section 9.4.

#### 2.1.4. Electronic Tongues and Fingerprint-Based Classification

Feedforward architectures are well-suited to generalization across whole patterns, especially within e-tongues and electrochemical fingerprinting. The feedforward architecture is capable of classifying many complex samples by means of a global profile of signals from multiple electrodes as opposed to peak signals of individual analytes. Such applications include authentication of liquors produced in specific regions at 100% accuracy using dimension-reduction techniques such as Laplacian Eigenmaps [44]; identification of 17 different plant species of Lycoris using pollen DPV fingerprints [45]; rapid screening for SARS-CoV-2 antibodies utilizing perceptrons to recognize laser induced graphene sensor immune response signatures [46]; classification of salivary voltammograms to screen for early detection of COVID-19 with 90% sensitivity and specificity [47]; and identifying breast cancer biomarkers (ECM1) through inhibition-based DPV immunosensing [48]. Neural networks outperformed traditional linear discriminant functions in all classifications; however, the diagnostic reliability of both was highly dependent upon the diversity, sample size, and the clinical representativeness of the training libraries [45,46].

Although the ANN has been successful as an electrochemical biosensor in many applications, there are four key systemic barriers that limit its use clinically and industrially. The first barrier to its use clinically and industrially is that there is still a severe lack of datasets, and most studies have fewer than 50–200 experimental points, leading to increased risk of overfitting and reduced generalizability of the model [29,39,49]. The second barrier to its use clinically and industrially is the “black-box” nature of ANNs. Only one study out of all the studies reviewed used either SHAP (SHapley Additive exPlanations or LIME (Local Interpretable Model-agnostic Explanations) interpretability frameworks, or provided any measure of prediction uncertainty; both of these are required to receive regulatory approval from agencies such as the FDA in order to utilize ANNs in clinical diagnostics [47]. The third barrier to its use clinically and industrially is limited access to data. Many of the datasets that were developed are only made available upon request, limiting independent validation, comparison/benchmarking, and utilization of models through transfer learning [32,39,48]. The fourth barrier is that, although ANNs achieve high accuracy under controlled conditions, these models often underperform in undiluted biofluids owing to unaccounted factors such as biofouling at the electrode surface, adsorption of proteins onto the electrode surface, and variability in electrodes fabricated [34,40,50]. To ultimately transition ANNs into deployable AI-based biosensors in laboratories, clinics, and industries, future development will need to focus on developing open-source electrochemical datasets, neural networks that can quantify uncertainty in predictions, and co-design of software and hardware.

### 2.2. Convolutional Neural Networks for Signal Processing

One-dimensional CNN (1-D CNN) architectures are becoming an increasingly important tool in a wide variety of applications for processing raw signals from electrochemical systems. The use of sliding window-based convolutional layers on current/potential and current/time data allows CNNs to identify hierarchical features within signals for tasks such as multi-analyte deconvolution; environmental calibration; and classification with an electronic tongue.

#### 2.2.1. Direct Processing of Raw Voltammetric Signals

One of the most significant benefits of 1D CNNs is their ability to bypass standard peak fitting/baseline subtracting methodologies. For example, parallel 1D CNNs were trained using raw differential pulse voltammogram data to both categorize and quantify Cd^2+^, Pb^2+^, Cu^2+^, and Hg^2+^ ions in water; this was done while maintaining an accuracy greater than 99% for all ion combinations [51]. Additionally, 1D CNNs have been employed as tools for identifying volatile organic compounds (VOCs) by analyzing cyclic voltammetry (CV) scan data at an accuracy rate of 99.09% [52] and as predictors of capsaicinoid concentration in complex food brine mixtures using multiple electrodes and CV and DPV matrix data [53,54]. In addition, a deep CNN has performed peak deconvolution on over 100,000 individual voltammograms and automatically calibrated those results within milliseconds to enable real-time analysis of voltammograms [55]. Therefore, these studies illustrate that CNNs may be extracting features that exist within the latent electrochemical signals that many shallow multivariate or univariate models would typically overlook.

#### 2.2.2. Multimodal Fusion and Environmental Calibration

The incorporation of CNNs into Dual-Pathway/Multimodal Frameworks is being adopted to mitigate cross-sensitivity as well as the impact of environmental changes on sensor calibration. Using two pathways with a modified one-dimensional Convolutional Neural Network (CNN), simultaneous pollutant feature extraction and temperature/humidity pattern recognition were used to maintain R^2^ ≥ 0.82 through all four seasons [56] in the calibration of low-cost air quality sensors. In addition, using an aqueous sensor system that combines the output of electrochemical voltammograms with the output of fluorescence spectroscopy, a one-dimensional CNN has been used to reduce mean absolute error to <0.61 μg/L when quantifying both Cd^2+^ and Pb^2+^ [57]. Furthermore, CNNs have also been used in conjunction with feedforward Artificial Neural Networks (ANNs) to identify whether ideal or faulty CV signal results should be generated based upon low-resolution images produced by a nanopipette-based interfacial tension (ITIES: Interface between Two Immiscible Electrolyte Solutions) sensor, thereby improving the quality control process [58].

#### 2.2.3. Neuromorphic and Edge-Deployable Implementations

The increased processing capabilities of CNNs allow them to be used in low-power applications such as microcontrollers or the new generation of low-power neuromorphic processors. An example is the implementation of an Intel Loihi processor, which uses a rate-based spiking CNN that was converted from a traditional ANN, and can perform classification on beverage type using real-time multi-sensor time series sensor data, and can achieve up to 97% accuracy at only 0.1 mJ per inference [59]. Another example is the use of 1-dimensional CNNs for self-powered corrosion-based pressure sensors for speech recognition, by classifying vocal patterns with 99.07% accuracy by using direct time series voltage signal output [60].

Despite having the ability for feature extraction, CNNs in electrochemical sensing have several translation issues. The first issue is their extreme data hunger; most studies rely on augmented or simulated datasets to avoid overfitting, yet these augmented datasets often fail to reflect the stochastic noise and matrix interference present in real-world samples, resulting in over-optimistic performance metrics that do not generalize to complex biological fluids [52,53]. The second issue is that CNNs act as opaque black boxes, with very few studies utilizing feature importance analysis to provide insight into what areas of the voltammetry generate the prediction [55]. The third issue concerns limited model transferability: most architectures are trained on a specific electrode geometry or buffer system, and their performance degrades substantially when applied to a different sensor batch or biological fluid [51,57].

### 2.3. Recurrent Neural Networks (RNNs) and Temporal Modeling

Recurrent architectures are uniquely suited in electrochemistry due to their ability to model temporal dependencies, sequential drift, and other forms of time series signals within electrochemical applications. A prominent recurrent architecture is “Long Short-Term Memory” (LSTM), which retains the previous state of the network, allowing it to model aging sensors, changes in the environment, and voltage scans.

#### 2.3.1. Modeling Temporal Dynamics in Environmental and Physiological Monitoring

LSTMs have also been successful at predicting the changing values of analytes over time in a dynamically changing environment. For example, an LSTM successfully modeled the hourly changes in dissolved oxygen levels due to eutrophication in an estuary over 124 days based on redox potential and other water quality parameters. The Root-Mean-Squared Error (RMSE) for this study was found to be 1.08 mg/L [61]. Another area that has seen success is in wearable sensing, where an LSTM was able to accurately predict riboflavin levels from sequential CV time series data as well as optimize the nanoplate doping ratio. This study found a 96.8% accurate prediction of riboflavin levels [62].

#### 2.3.2. Attention-Enhanced and Hybrid Sequential Architectures

To reduce ambiguity of signals and provide a focus on those regions of interest where redox reactions are most likely to be occurring, attention has been added to both LSTMs and fully convolutional networks. Specifically, an Attention LSTM-FCN model (ALSTM-FCN) was used to classify various types of heavy metals, pesticides, and industrial contaminants within seawater samples through cyclic square-wave voltammetry (CSWV) signatures and demonstrated >80% accuracy as well as the ability to construct dose–response curves [63]. Additionally, a comparison of several sequential classification techniques showed that both LSTM and FCN-based models were superior to the other two models, Principal Component Analysis–Support Vector Machine (PCA-SVM) and dynamic time warping (DTW), in terms of identifying chemical threats within the multi-class seawater and explosive datasets tested using receiver operating characteristic area under the curve (ROCAUC) > 0.99 [64]. The use of attention maps in such architectures may also serve to indicate areas in which redox reactions are likely to occur, thereby reducing some degree of the ‘black-box’ characteristics associated with deep sequential models.

Training sequential models using long time series data is generally possible using GPUs; however, this can conflict with the low power requirements associated with POC devices [64]. LSTM architectures are sensitive to sample size; training with fewer than 100 sequential samples has been shown to cause overfitting and poor classification, particularly for low-concentration analytes whose weak signals yield ambiguous temporal patterns [62,63]. Most sequential models are tested off-line and there are relatively few examples of ongoing inference occurring continuously while running sequentially on edge microcontrollers. Integrating compact temporal models (Temporal Convolutional Networks or Distilled LSTMs) into microcontrollers is an important next step toward enabling real-world applications.

### 2.4. Advanced Optimization Strategies: Evolutionary Tuning and Custom Training Dynamics

Hybrid models based on standard ANNs are being designed to overcome some of the most common limitations of ANNs, including their inability to avoid local minima and sensitivity to hyperparameters and the inefficiency of training ANNs with data from electrochemistry. A hybrid model combines a neural network with an evolutionary algorithm, custom loss function, and automatic selection of hyperparameters to better support the process of fabrication of sensors and improve the predictive ability of the model.

#### 2.4.1. Evolutionary Optimization of Sensor Fabrication and Hyperparameters

The coupling of genetic algorithms (GAs) to ANNs has proven useful in optimizing parameters associated with electrochemical sensors. A GA-ANN model achieved an R^2^ > 0.99 using only 19 experimental data points and predicted current responses as a function of pH, electrolyte concentrations and accumulation times for heavy metals [65]. Similarly, a GA-BP-ANN hybrid reduced the detection limit to 0.003 µg/L for detecting Pb^2+^ [66], through optimization of deposition potential, time and volume of deposited nanomaterials. In contrast, while Extreme Gradient Boosting (XGBoost) was initially examined for nitrate sensing, it was ultimately replaced by custom-tuned ANNs with SELU and LeakyReLU activation functions to provide superior predictive performance in terms of modeling pulse-electrodeposition parameters; this highlights the importance of selecting appropriate activation functions specific to the type of neural network architecture employed to represent non-linear fabrication parameter space [67].

#### 2.4.2. Custom Loss Functions and Multi-Pathway Regression

To address systematically biased drug quantification, a novel Two-Way ANN architecture with a Weighted MSE loss function was proposed, achieving accuracy of up to ~87% on real cyclic voltammograms of etoposide and methotrexate mixtures [68], demonstrating that it is possible to greatly improve the fidelity of regression by changing the optimization goal instead of simply increasing the number of layers of the networks; hybrid pipelines for combining simulated data creation using computational models with experimental validation allow to increase the reliability of models, especially in cases where there are no enough real-world datasets available [68].

Hybrid ANN systems produce improved predictive power at a cost of increased complexity and poor reproducibility. The parameters for both the Genetic Algorithm (GA)-ANN and Custom-Loss models have to be optimized computationally extensively with little opportunity to port an “optimal” configuration among various analyte/electrode types [65,66]. Most hybrid model studies employ very limited experimental datasets (<50 pts) and therefore it is unclear if performance improvements occur through genuine generalized predictions or through fitting to some form of laboratory-specific noise [67,68]. Moreover, hyperparameters (e.g., mutation rate, population size, loss-weight schemes) are reported inconsistently across studies, limiting independent reproducibility of hybrid models. A comprehensive comparison of the theoretical bases, applicability, and performance metrics of primary AI/ML architectures reviewed in this section is summarized in Table 1.

### 2.5. Deep Neural Networks and Specialized Models

Beyond standard ANNs/CNNs, new deep-architecture designs were developed that can be used in multiple tasks (multi-task), biologically inspired classification, and to automatically screen drugs. These new model types are able to use deeper hierarchies of features/representations, prototype-based reasoning, and branches of processing paths to handle difficult electrochemical problems.

#### 2.5.1. Multi-Task and Multi-Branch Architectures for Therapeutic Monitoring

The use of an architecture specifically designed for the IdentQuantNet, such as a two-branch Deep Neural Networks (DNNs) in which drug classification is separated from the determination of drug concentrations by using CV feature extraction based on Gaussian distributions, has demonstrated 100% accuracy in classification of chemotherapeutic drugs (cyclophosphamide vs. ifosfamide) and a mean absolute error (MAE) of less than 6.67 µM [77], depending upon drug concentration range.

#### 2.5.2. Bioinspired and Prototype-Based Deep Learning

Prototype-based DNNs have also been developed for electronic tongue systems as an analog to human sensory classification. A soft-voting DNN was used to classify six different types of wines using the open-circuit potential and EIS data collected from lipid-membrane sensors. The DNN produced a 95% accurate classification rate and maintained a 90% classification rate even after one-third of the total number of samples included artificially added noise [78]. In gas sensing applications, combinations of deep networks, PCA, and KNN/SVR baseline methods were utilized to deconvolve overlapping redox signals for NO_2_ and CO_2_. However, results showed that while the DNN performed well when there was an abundance of data available, a KNN method performed better than the DNN methods when working within low-data regimes [79].

Limited validation and significant data demands characterize specialized Deep Neural Networks. The majority of multi-branch and prototype-based models were trained using simulated or buffer-spiked datasets with little testing done in undiluted biofluids that can cause distortion in deep feature extraction due to protein fouling and ionic strength changes [77,78]. Furthermore, the degree of architectural complexity is far ahead of interpretability; no studied DNN provided layer-by-layer relevance propagation or visualized an “attention” process to describe the mapping of voltammetric characteristics to a clinical output [77,79]. Lastly, multi-branch DNNs need cloud or desktop inference and therefore are not able to have real-time point-of-care application. A comprehensive visualization of these trade-offs is presented in Figure 3, which maps the co-occurrence of strength and weakness for all ANN-based architectures reviewed.

## 3. Support Vector Machines and Kernel Methods

Support Vector Machines (SVMs) remain a major part of electrochemical data analysis because of their effectiveness at identifying patterns in complex datasets; such models can be trained with very small training data; and as such, provide some assurance that the model is generalizable. The two primary types of SVMs utilized are SVC (classification) and SVR (regression). Additionally, LS-SVM has also gained widespread use for various applications including but not limited to diagnostics, authentication and interference-compensated quantification.

### 3.1. Support Vector Classification for Diagnostic Screening

SVC models are able to discriminate between classes of samples (binary or multi-class) using electrochemical sensing, which may have very slight, non-linear separation. A model that used an RBF kernel was able to detect urine samples positive for Zika virus with 91% accuracy based on both absolute and relative changes in the LSV signal [80]. In addition, SVMs were used to authenticate different red wine types from boron-doped diamond CV fingerprints and resulted in an authentication rate of 96% [81]; similarly, SVMs were used to distinguish dengue virus serotypes from EIS-derived Randles circuit parameters, resulting in an identification rate of 94% [82]. In food and forensic-related applications, SVMs were used to classify tea antioxidant capacities with an accuracy of 93.5% [83] and identify illicit drugs from multi-pH square-wave voltammetry super-fingerprints with an accuracy of 91.8% [84]. These examples illustrate the reliability of SVM for fast, label-free diagnostics and authenticity testing.

### 3.2. Support Vector Regression for Quantitative Analysis

SVR and LS-SVM algorithms are preferred over ANN models when working with smaller datasets and where a stable prediction is necessary over raw predictive power. Using Bayesian hyperparameter optimization, an SVR algorithm was able to predict Dopamine using paper-based sensors made of MoS2 with a 98.87% [85] rate of accuracy. The SVR also improved the accuracy of nitrogen dioxide gas (NO_2_) measurement in mobile air quality monitors that were affected by both temperature and humidity, resulting in a maximum reduction in Mean Squared Error (MSE) of 60% [86]. Multiple variations in LS-SVM have shown significant improvement in predicting agrochemicals using ANN models; R^2^ values greater than 0.997 were achieved for maleic hydrazide [87,88], clenbuterol [89], benomyl [90], hesperitin [91], and salicylic acid [92]. The mathematical formulation of LS-SVM differs significantly from that of ANNs because it reformulates inequality constraints as equality constraints, allowing for faster processing time, as well as better generalization to limited electrochemical datasets. Key implementation details, performance benchmarks, and identified limitations of SVM-based approaches are compiled in Table 2.

### 3.3. Hyperparameter Optimization and Kernel Engineering

The performance of the SVM-based model is mainly determined by the choice of the kernel and parameters. Particle Swarm Optimization (PSO) has been combined with SVM to predict nitrates under the influence of chlorides, which showed an R^2^ value of 0.997 [93]. Gray box configurations of SVM using Gaussian and linear kernels have been used in gas sensing to minimize the cross-sensitivities due to ammonia (NH_3_) in oxygen (O_2_), nitrogen oxides (NOx) sensor systems. These gray box SVM configurations achieved a correlation coefficient (R^2^) value close to 0.99 [95]. The comparative studies clearly demonstrate that RBF kernels perform better than other kernels, including Gaussian and polynomial functions, in the non-linear voltage regression of voltammograms; however, linear kernels can be used for EIS and square-wave voltammetry (SWV)-based classifications when the features are well separated [82,84]. Although SVM methods have been shown to be very robust, there are still limitations of SVM-based methodologies in terms of scalability and interpretability. As shown in Figure 4, while SVM models demonstrate robustness in small-data regimes (high ‘low training resource’ scores), they consistently lack ‘uncertainty quantification’, limiting the clinical regulatory approval potential.

As datasets grow in size, training times increase quadratically as well as the number of samples (the maximum sample count that can be used to train an SVM model is approximately 10,000) [84,97]. In addition, SVM models generate point estimates without inherently quantifying uncertainty associated with those predictions, which makes them less useful for clinical applications such as decision-making where confidence intervals are a necessity [84,96]. Lastly, selecting appropriate kernels for SVM models has historically remained empirical; the electrochemical rationale for selecting one kernel over another has rarely been demonstrated; further, performing grid searches to find optimal values for kernel parameters is significantly less efficient than using either Bayesian methods or gradient-based methods for optimizing parameter values [85,93]. It appears that integrating probabilistic SVM variations into future generation deployable sensor systems along with automated kernel selection will be required.

## 4. Tree-Based Models and Ensemble Methods

Tree-based machine learning (Decision Trees, Random Forests, Gradient Boosting) is becoming the dominant paradigm in AI-enhanced electrochemical sensing. Tree-based approaches offer natural robustness against overfitting; can incorporate both numerical and categorical variables into an algorithmic framework; and can easily assess variable contribution to a given model’s performance.

### 4.1. Decision Trees for Threshold-Based Classification

Decision Trees provide transparent, rule-based reasoning that can determine when a sample exceeds a given limit-of-detection (LOD) threshold and can also provide a basis for rapid screening. A two-stage Decision Tree pipeline first classifies Dopamine and serotonin samples as being either below or above an operationally defined LOD. Then, Decision Tree regressions are used to quantify each of these analytes. This was shown to improve the LOD by greater than 60-fold over single-mode voltammetry [100]. Similar two-step Decision Tree (DT) regression approaches were developed to enable simultaneous analysis of tyrosine and uric acid in both sweat and saliva through fusion of multimodal voltammograms [101]. The combination of DT with k-Nearest Neighbor (KNN) algorithms also resulted in an improvement of 8× in the LOQ for neurotransmitters measured in artificial saliva [102]. The ability to assign values to Decision Tree split criteria based on physiologic and chemical principles makes Decision Trees very attractive for establishing regulatory-compliant diagnostic thresholds. Table 3 summarizes the architectural choices, predictive performance, and computational requirements of tree-based and ensemble methods, illustrating the hardware–software mismatch discussed above.

**Table 3:** Summary of characteristics and performance of tree-based and ensemble learning in paradigms in electrochemical biosensing in food, biological and environmental matrices.

Algorithm Architecture	Electrochemical Method	Sensor Material	Linear Range	LOD	Electrochemical Characteristics		AI/ML Model Characteristics			Matrix	Ref.
					Adv.	Disadv.	Input	Output	Performance		
DT, KNN	DPV, SWV, LAACV	AA-LIG	–	–	A, D	C, E	Multi-peak parameters from DPV/SWV/LAACV: Train:Test::90:10, leave-one-out	Neurotransmitter conc. (DA, SER, EP, NEP)	LOD improvement: 8× vs. single-peak	Artificial saliva	[102]
Two-step (Binary classifier + DT)	CV, SWV, DPV, LAACV	eMoS_x_-LIG	–	–	E	C, D	Multimodal electrochemical data: Train: 1 mixed analyte; Test: Unseen mixed samples	TYR and UA conc.	R^2^: 0.85 (UA), 0.95 (TYR) in sweat; LOD improvement: 100×	Sweat & saliva	[101]
DT Classifier and Regressor	DPV, SWV	NF-LIG	0.005–500 μM	5 nM	A, B, C, D, E	–	DPV & SWV voltammograms (peak heights, positions, widths)	Classification (above/below threshold) and Conc. for DA and SER	A_pred_: 85–90%; R_pred_: >65%; Pearson R > 0.8; Recovery: 91–108%	Undiluted human urine	[100]
RFR	DPV	NiCoMn-MOF/PCL@PD	0.005–1 μM	0.00238 μM	A, B, C, D	E	Metal conc.: Ni, Co, Mn ratios, Test: Validation sets for ML predictions	Voltametric current response	R^2^: 0.94857	Agricultural soil	[103]
RF	DPASV	ErNBC	80–1200 μg/L	0.49 μg/L	A, B, C	–	DPASV current (ΔI)	Pb^2+^ conc.	R^2^: 0.9985 (train), 0.9877 (test); RMSE: 13.99 (train), 35.65 (test); RPD: 9.03	Water samples	[104]
RF	i-t Amp	Metal electrodes	–	–	D, E	C	Collective sensor voltage responses (6 m. hourly data), Train site: Athens, Test site: Patras, 1-week inter-sensor consistency validation	CO, O_3_, NO_2_, NO conc.	Test site: CO: R^2^ = 0.64, O_3_: R^2^ = 0.58, NO_2_: R^2^ = 0.31, NO: R^2^ = 0.35	Ambient air	[105]
RF, ANN	EIS	Stainless-steel capillaries; Au IDEs	0–115 g/L total ions	–	B, D	C	Whole impedance spectra Train samples: 50 produced water and 49 crude oils, Test: 20% real samples	Multi-output regression of ion conc. (Mg^2+^, Ca^2+^, Sr^2+^, Cl^−^)	Produced water Acc.: 96.7–99.2%, Crude oil Acc.: 95.2–99.8%, MAE: <5%	Aqueous samples from oil	[106]
SVM, RF	DPV	CuO/RGO/NPAN composite	0.5–100 μM	0.19 μM	A, B, D, E	C	DPV data	Pesticide classification (carbendazim identification)	Accuracy: 89.87–100%	Tea samples	[107]
RF, SVR	SWV	VP-PCM	2.49–71.14 μM	0.0187 μM	A, B, C, D, E	–	SWV data	MPA conc.	R^2^: 0.9999, MAPE: 0.0081	Corn silage and wheat silage	[108]
LS-SVM, ANN, RF	DPV	LIPG	–	–	C, D, E	–	Peak currents from DPV: Approximately 34 points per range; Train: 70% of each range	MH conc.	R^2^: 0.9999 (train), 0.9994 (test) RMSE: 0.388 (train), 0.7717 (test) RPD: 42.4412 Recovery: 99.07–104.80%	Potatoes, peanuts	[88]
RF, SVM, ANN	SWV	SPE/THC or CBD	0–5 ng/mL	–	E	B, C, D	SWV signals: entire sweep scans; Train:Test::80:20; 122 instances (THC), 181 instances (CBD)	Classification of THC/CBD conc. (0, 2, 5 ng/mL)	Accuracy: 92% (THC), 84% (CBD) for RF with PCA	Human saliva	[109]
RF	i-t Amp, EIS	Gold-coated needle electrodes	3–15 mM	–	C	B, D, E	EIS parameters (Cd1, Rs): *N* = 54 data points; Train:Test::70:30	Glucose sensor sensitivity (nA/mM)	MAE: 1.50 nA/mM	Artificial ISF	[110]
Random Forest	EIS	3-APBA cell-imprinted polymer/AuE	10–10^6^ CFU/mL	10 CFU/mL	A, B, C, D, E	–	Six EIS parameters (R_ct_, R_s_, W-R, W-P, W-T, CPE) from Nyquist plots: *N* = 2592 spectra; Train:Test::80:20	Classification and conc. of three bacteria (*EColi*, *StaphA*, *VibrioP*) via 18 labels	Accuracy: 95%, Loss rate: 5%	Seawater, milk	[111]
RF, FNN	CA	SPCE	0.02–2.5 μM	–	C, D, E	B	ECL images and amperograms: Train: 105 interpolated points; Test: 3 experimental points	Ru(bpy)_3_^2+^ conc.	R^2^: 0.996 (RF), 0.961 (FNN) MSE: 0.0012 (RF), 0.0356 (FNN)	Aqueous solution	[112]
ANN, SVM, RF	CA, CV	SPCE/chitosan–gold NPs	0.5–90 ppm	0.1 ppm	A, B, C, D, E	–	1050 data points (21 conc. × 5 temps × 10 lifespans); CA current, electrode lifespan, storage temperature	Histamine conc. (0–200 ppm)	R^2^ = 0.95, RMSE = 13.2 ppm (ANN-GA, 40 d.)	Beef and trout	[113]
KNN, RF, SVM, GP, LR, Multivariate	SWV	Stencil-printed carbon	0–16 μM	1.18 μM	A, D, E	B, C	9 voltammogram features (Min dN/dV), *N* = 225 (Epsilon dataset)	Conc. for CBZ	R^2^: 0.84–0.87 %error: 19% (KNN/RF Epsilon) 19% (KNN Delta)	Spiked saliva	[114]
ANN, BP, RBF, RF	DPV	Ag/ZIF-67/g-C_3_N_4_/GCE	0.01–250.00 μmol L^−1^	5.32 nmol L^−1^	A, B, C, D, E	–	Current values from electrochemical sensor (12 train, 8 test)	Chloramphenicol conc.	RF– R^2^: 0.99834; RMSE: 0.00403; MAE: 0.00315	Pharmaceuticals, urine	[115]
ANN, RF	DPV	NiO/GCE	16.1–58.8 μM	1.3–2.61 μM	A, D, E	B, C	150,600 DPV signals (potential, current—4 electrodes); 10 plasma samples (1:9, 1:15, 1:30 dilutions)	L-lactic acid conc.	R^2^: 0.9749–0.99; RMSE: 0.05436—0.0995	Plasma, PBS buffer	[116]
DT, RF, KNN, SVM, LR	DPV, CV, LSV, EBFC	GDY/AuNPs/carbon	0.01–10,000 ng/mL	0.0033–0.0081 ng/mL	A, B, C, D, E	−	Pre-treated milk (centrifuged, diluted 1000×); low-, medium-, high-conc. sets	Classification (low/med/high conc.) & quantification (β-LG conc.)	Acc.: 93%; AUC: 0.93–0.99	Milk	[117]
ANN, RF, KNN	DPV, CV, EIS	CuNW/MSQD	1.96–966.0 μM	0.001–2.3 μM	A, B, D	C, E	7–11 input features (potential, current, chemical, samples, number of folds, resistance, peak potential, etc.)	Creatinine conc.	R^2^ = 0.679–0.984; RMSE = 0.017–193.66; LOD = 0.001–4.49 μM; LOD = 4.33 μM	Mixture with interferents, urine	[118]
SVM-PCA, RF-PCA, ELM-PCA	DPV	RGO/SPE	5–40% adulteration	2.8% adulteration	C, D	E	460 samples (360 authentic + 100 adulterated), 601 DPV signals—5 PCA features	Authentic/counterfeit/adulterated antler samples	Acc.: 97.9%; Sens.: >97%; Spec.: >98%; Validation Acc.: 97.0%	Sika deer antler powder	[119]
RF, SVR, PLS	SWASV	Bi/GCE	2–175 μg/L	–	B, D	C, E	SWASV stripping currents (90 for Cd, 98 for Pb); 64 samples (43 calibration, 21 validation)	Cd^2+^ and Pb^2+^ conc.	R^2^v = 0.992–0.985, RMSEV = 1.439–5.348 μg/L	Soil extracts	[120]
DT, RF, GB, XGBoost, Bagging	DPV	PPy/MWCNT/GCE	–	–	C, D, E	–	MIP synthesis param.: MWCNT volume, pyrrole conc., DOX conc., CV scans, scan rate, overoxidation cycles, incubation time, DOX-to-pyrrole ratio (total data points NS, small dataset)	DOX oxidation peak (sensor output current)	R^2^: 0.993; RMSE: 0.436; MAE: 0.244	Phosphate Buffer	[121]
CatBoost	DPV, CV	AuNPs/CHI/GCE	0.0–1.25 μM	0.049 μM	A, C	–	Electrochemical parameters: potential, scan rate, temperature, pH; *N* = 2319 datapoints, Train:Test::80:20	Cathodic current (μA) for CIP conc.	R^2^: 0.9986; SE: 1.3171; RMSE: 1.1476; MAE: 0.5923; MedAE: 0.2954	Aqueous solutions	[122]
ANN, SVM, XGBoost, OLR	CV	PANI: D9B NCs	0.625–5 μM	0.078 μM	A, D, E	B	Features from CV scans: Train: LOOCV (n− 1 samples); I_max_, I_min_, Istd, slope, peaks count, symmetry; Test: 1-sample per fold) from D9B sensor	DA conc.	ANN (best model): MSE: 0.1699; MAE: 0.3817; RMSE: 0.4122; R^2^: 0.9395	Phosphate buffer	[123]
XGBR, RFR, DTR	i-t Amp	PEDOT-C@Cu-NPs	5–580 μM	3.91 μM	A, B, C, D, E	–	Experimental parameters: pH, drying time, conc.—Train set; Test: grid search with cross-validation	Current	R^2^ suitable for XGBR/RFR/DTR; Grid search optimization	Pickled vegetable extract	[124]
LR, LSR, ENR, DTR, RFR, KNNR, SVR, XGBR	i-t Amp	CN-CoNPs-CP@NF	0.002–220 μM	0.00114 μM	A, B, C, D	E	Experimental parameters: pH, drying time, material conc.	Current response	R^2^ SVR: −0.2; R^2^ LR, DTR, RFR, XGBR > 0.95; R^2^ LSR > 0.75; R^2^ ENR, KNNR > 0.35	Beef meat extract	[125]
XGBoost	EIS, CV	MIP/AuNPs/PEI-MWCNTs/GCE	0.5–70 μM	0.078 μM	A, B, C, D	E	EIS parameters (Rp, Rs, W-P, W-R): 500 impedance datasets (100 data points per conc.)	Conc. of propachlor	RMSE minimized during training	Food samples	[126]
XGBoost, ANN, RF, FB Prophet	i-t Amp	BES	–	–	C, E	D	BES signal and process variables (9 m., 10 min. intervals); Test: validation subset	MNR and influent events	R^2^: 0.872, RMSE: 414.4 lb/day	Wastewater	[127]
CatBoost, Fedot	CV	Cu, Ni, C fiber electrodes	–	–	D	C, E	CV data from multielectrode: Train: 1377 scans (5 antibiotics); 2122 scans (15 antibiotics); Test: 20–50% of total scans	Antibiotic classification	Accuracy: 0.99 (antibiotic), 0.74 (milk); F1: 0.9533, 0.8387	Milk	[128]
NN, SVM, RF, GB	CV	Pt	–	–	C, E	D	Anodic CV slope, initial L-histidine conc., antisolvent ratio: *N* = 40 points; Train:Test::70:30	Supersaturation	Acc: 97.4% (NN), 91.7% (SVM), 79.7% (RF), 90.8% (GB) Std: ±3.7% (NN), ±8.7% (SVM), ±23.1% (RF), ±8.0% (GB)	Water–ethanol	[129]
RF, ET, XGBoost, LR, SVM, KNN, DT, GB	CV	Co@Cu/GCE	0.00–4.00 mM	0.13 mM	C, D, E	A, B	56 CV scans (7 conc. × 8 replicates); 10 features (peak heights)	Creatinine conc.	R^2^: 0.361–0.916	Urine samples	[130]
RF, XGBoost, LightGBM, ANN	CV	GAgNPs/Au	5–100 U/mL	0.07–0.11 U/mL	A, B, C, D, E	–	CV-derived features: charge, peak potentials; 17 tumor samples and 1 control; LOOCV	Binary tumor grade class (CA 15-3, MUC-1)	Tumor detection: 95–98% Tumor grading: CA 15-3: 62–76%)	Serum, tissue	[131]
LR, DT, RF, KNN, AdaBoost, GB	CV	MWCNT-ZnO/CuO-MFs	500–8000 μM	0.0785 μM	A, B, C, D	E	CV measurements: current values from CV; *N* = 200; Train:Test::80:20	Urea conc.	MAE: 0.0001 MSE: 1.93 RMSE: 0.0004 R^2^: 0.981 (KNN)	Simulated blood matrix	[132]
GBA	CV	PGE	2000–10,000 μM	–	B, D, E	C	CV scans: *N* = 100 variants, 5 instances each; Train: 70% and 60% of the variants; Test: 30% and 40% of the variants	Conc. classification of potassium ferricyanide	Acc.: 60–76.6%	Nitric acid solution	[133]
AdaBoost, CatBoost, GB, RF, SGD, MNB, Quadratic Discriminant	EIS	ZIF-8 IDEs	31.25–4000 ng mL^−1^	0.2419–0.3495 ng mL^−1^	A, B, C, D	E	*N* = 60 OSCC saliva samples (30 N0, 30 N+), 4:1::train: test, EIS features at 0.4 Hz	Class. (N0 non-metastatic/N+ metastatic OSCC prognosis)	LTA4H (CatBoost tuned): AUC 97%; Combined (AdaBoost): AUC 0.76 F1-score 0.78, Balanced Acc. 0.78	OSCC patients’ saliva samples	[134]
GPR, XGBoost, ANN	Cyclic Potential Scan	CP-Glucose Oxidase-GA	–	–	C, D	E	Fabrication param. (Enzyme amount, GA conc., pH, CP scan number, Analyte conc.), Synthetic + Bootstrapping	Regression (I_P2_ current)	RMSE = 0.1465; MAE = 0.0865; R^2^ = 1.00 (Best models: DT, GPR, ANN)	Buffer	[135]
CatBoost (two-stage hybrid)	DPV, CV	His-pHEG/NF/SPCE	0.28–11.36 μM	0.09 μM	A, C, D, E	B	Electrochemical DPV response data (stratified balanced)	Classification (AA Presence/Absence), Regression (Conc.)	Acc.: 86.19%; Prec.: 92.42%; Rec.: 92.42%; F1: 92.42%; MAE: 0.0514; RMSE: 0.0737; R^2^: 0.7039; Hybrid Score: 0.8141	Lemon juice	[136]
ANN, XGBoost	DPV	Carbon slurry	50–1000 mmol L^−1^	–	B, D, E	C	728 DPV scans (70:30::train: test), 88 features (class.), 101 features (reg.), SG Smoothing & Normalization	Classification (AA, DA, UA combinations), Regression (Conc. AA, DA, UA)	ANN–Acc.: 94.06%; XGBoost R^2^: 96.2% (Avg)	PBS buffer	[137]
LR, ANN, RF, XGBoost, LightGBM	CV	PANI: Co, Cu, Ni, Zn (II)/Orotate NCs	0.3125 μM–5 mM	0.33 μM–1.89 mM	A, C	B, D, E	CV signals (charge, skewness, peak current, potential); LOOCV	Regression (Dopamine, Glucose conc.)	R^2^: 0.7598; MAE: 0.561–2939; RMSE: 0.835–3001	–	[138]
KNN, LR, RF, SVM, Gaussian Naïve Bayes, LDA, AdaBoost, XGBoost	CV, DPV, SWV	ZnO nanoflowers	10 fg/mL–10 ng/mL	0.32 fg/mL	A, B, C, E	D	9135 (315 × 29); 4 datasets (DPV & SWV for cTnI & cTnT); 15 data points per conc. class augmented 45 each; 75:25:: Train: Test split	7 conc. classes for Cardiac Troponin-I and Troponin-T	Physics-informed features–Acc.: 100%; AUC: 100%	Plasma	[139]
ANN, XGBoost, LightGBM, RF, SVM	DPV	Oxygen vacant-CoV_2_O_6_ microspheres	0.15–4.0 μM	0.03 μM	A, C, D, E	B	DPV voltammograms (current–conc.); skewness, kurtosis, symmetry	HVA conc.	MAE: 0.2927; MSE: 0.2112; R^2^: 0.8566; RMSE: 0.4595	PBS (pH 6.5)	[140]
SVM, KNN, NB, DT, RF, GBT	SCV	RGO, GO-1, GO-2/Au	0.145–25.39 ng/μL	0.104 ng/μL	A, B, C, D, E	–	232 signals, 103 features, 2 RFE-features (SWAB-PBSmax, SWABMaxStepCurrentR8)	Presence/Absence for Rabies	Acc.: 98.33%; Pcs.: 98.89%; Spec.: 99.80%; F2: 0.9830	Bat nasopharyngeal swabs	[141]
XGBoost	EIS, CV, CA	SPE/Tyr/HSA/GA	–	–	C, D, E	–	EIS feat. (R_ct_, C_dl_, impedance magnitude/phase spectra) from equivalent circuit modeling; 3 juice types with CV	Antioxidant capacity, TEAC values	MAE: 0.341; RMSE: 0.458; R^2^: 0.983; AUC: 0.92	Fruit juices	[142]
LR, DT, RF, SVM, GB, OLS, GLM	CV, DPV	Au/MIL-101(Cr)–NH_2_	0.5–100 μM	0.011 μM	A, B, C, D	E	Conc. (μM), Scan rate (mV/s), pH, Peak potential (V), Time	Predicted current (μA) for caffeic acid detection	R^2^ = 0.9969 (DPV conc. Vs. I_pa_); R^2^ = 0.9992 (I_pa_ vs. V^1/2^); R^2^ = 0.9996 (pH vs. E_pa_)	Coffee, red wine	[143]
XGBoost, RF, DT, ANN, MLR	DPV	Carbon electrode	0.1–0.5 mmol/L	100 nmol/L	A, C, D, E	B	825 DPV scans (80:20:: Train: Test). Features: Peak apex, ABTS vol, HRP/GOx vol, Scan rate	Glucose conc.	R^2^: 0.928; MAE: 13.931 μmol/L; RMSE: 31.034 μmol/L	PBS solution	[144]
LSBoost	DPV	AuNF/(IL/GO)/SPCE	–	0.58–0.59 pg/mL	A, C, D, E	–	640 param. (DEHP and BPA: 320 each), 8 multi-indexes (Ip_0_, Ep_0_, Ap_0_, I_p_, E_p_, A_p_, ΔI_p_, ΔI_p_/Ip_0_) pH 4.5–10.5	DEHP and BPA conc.	MAE, MSE, RMSE, RRMSE < 10%; Relative error < 6% compared to LLE-GC-MS	River water	[145]

**Electrochemical Sensor Characteristics**. Advantages: (A) low LOD, (B) wide LDR, (C) selective electrode, (D) high reproducibility of sensor, (E) low deposition time. Disadvantages: (A) high LOD, (B) narrow LDR, (C) non-selective electrode, (D) low reproducibility of sensor, (E) high deposition time, Conc.—concentration.

### 4.2. Random Forest for Robust Regression and Feature Analysis

Random Forest’s use of bootstrap sampling and out-of-sample error estimates provides an impressive level of robustness to fit the complex relationships involved in electrochemical modeling. The Random Forests developed to find an optimal trimetallic Metal–Organic Framework (MOF) for the detection of p-nitrophenol [103] were able to predict the concentration of Pb^2+^ from nano-biochar sensors with a coefficient of determination R^2^ = 0.9877 [104]; also, the authors successfully calibrated multiple air-quality sensor networks across urban environments based on the cross-sensitivities among adjacent electrodes [105]. In addition, Random Forest was used to model histamine levels in meats at electrode lifespans extending up to 40 days [113], classify pathogenic bacteria using EIS Nyquist plots with 95% accuracy [111] and authenticate deer antler powder samples via their chemical composition with 97.9% accuracy [119]. In each case, the features identified by RF that are most relevant to the task are always peak current, separation potential and phase angle.

### 4.3. Gradient Boosting and Stacking Ensembles for High-Precision Quantification

Sequential error correction by boosting algorithms provides high levels of performance on difficult quantification problems. For example, XGBoost was used to optimize nitrite concentration in preserved foods like pickled vegetables [124], as well as to predict clenbuterol in meat extract samples [125], and to use EIS data to estimate total antioxidant capacity of fruit juices [142]. Similarly, categorical boosting (CatBoost) has been shown to excel when dealing with categorical variables; examples include prediction of ciprofloxacin cathodic current values with an R^2^ value of 0.9986 [122] and multi-stage classification/regression of vitamin C content in lemons [136]. As such, stacking ensembles consisting of Decision Trees (DTs), Fandom Forests (RFs), Gradient-Boosted (GB), XGBoost, bagging using DT were combined with a DT meta-model which resulted in an R^2^ value of 0.993 for optimizing the response time of a doxorubicin microcantilever sensor based on a MIP film [121]; this demonstrates that diversity among models can help mitigate bias within each model.

While tree-based ensembles offer high classification accuracy, these models also exhibit limited edge compatibility compared to other methods. For example, training CatBoost/XGBoost on more than 1000 voltammetric data points typically requires a GPU; this contradicts the low-power design constraints of POC devices [135,145]. Another limitation of these types of models is their inherent “black-box” nature. While these ensembles yield feature importance scores quantifying the relative contribution of each input variable, the underlying basis for the misclassification of a particular voltage sweep curve remains insufficiently elucidated. Figure 5 illustrates that while tree-based ensembles achieve high predictive precision, they frequently suffer from ‘High Computational Latency’ and ‘Edge-Deployment Barriers,’ confirming the mismatch between model complexity and point-of-care hardware constraints.

In addition to the lack of local interpretation of tree-based ensemble models, nearly all studies reporting results using tree-based ensemble models fail to report prediction intervals or conformal prediction bands around the predicted class probabilities [113,139]. A third major drawback of tree-based ensemble models is their sensitivity to changes in the fabrication process for the electrodes used in the experiments. Models trained using data collected during one fabrication run may perform poorly when applied to data collected from new batches of fabricated electrodes due to differences in the surface morphology that were not modeled during the calibration phase [117,144].

## 5. Linear and Multivariate Regression Models

Techniques of linear and multivariate regression continue to be an essential part of electrochemical analysis, which are commonly used to calibrate baselines, correct drifts and perform multivariate deconvolutions. Although neural or ensemble techniques offer much greater flexibility than these classical techniques, the transparency of linear and multivariate regression techniques, along with their computational efficiency and statistical properties, makes them a staple in validating sensors and performing quality control.

### 5.1. Ordinary Least Squares for Baseline Calibration

Univariate linear regression (ULR) and ordinary least-squares (OLS) regression methods have been used extensively to quickly provide an interpretive model for calibration of wearable sensors and microneedle sensors. The ULR method has successfully correlated the peak SWV current values with buprenorphine concentrations in interstitial fluid (ISF), R^2^ = 0.985 [146], while also allowing on-site lidocaine detection using a Flask web application [147]. OLS regression was innovatively utilized as an in silico drift corrector within dual-Chronoamperometry by determining the correlation between reference and test current waveforms so that biofouling-induced signal decay can be normalized among Interferon-gamma (IFN-γ), Dopamine, and DNA targets [148]. These examples show that linear models can be very useful in real-time low compute monitoring when the priority is transparency rather than non-linear fitting.

### 5.2. Partial Least Squares and Principal Component Regression for Multivariate Deconvolution

Partial least squares (PLS) and principal component regression (PCR) are ideal when there is significant peak overlap or high-dimensional collinearity of electrochemical signals. The PLS regression was able to quantify levels of the pesticide malathion from DPV inhibition ratios with a prediction error ≤ 5% [149], and resolve overlapping morphine, methadone and uric acid signals in urine at a Root-Mean-Squared error of prediction (RMSEP) ≈ 0.16–0.20 μM [150]. For protein detection, PLS was superior to SVM and RF for determining human serum albumin concentrations from surface-enhanced Raman scattering (SERS)-electrochemical deposition spectra (R^2^ = 0.98) [151]. The interpretability-performance trade-off of regression techniques is detailed in Table 4, which contrasts linear methods with multivariate approaches across diverse analyte matrices.

Similarly, PCA combined with PCR determined caffeine content in commercial coffee products with an accuracy of 93.88%, combining concrete voltage coordinates with abstract spectral area features [152]. Latent variable techniques will continue to be necessary for multi-analyte determination without extensive feature extraction.

Models based on linear assumptions (latent linear structure) can be adversely affected by non-linear electrode fouling, saturation effects, and complex interactions within a matrix, as well as being limited by their inability to adapt when an initial calibration has been made (i.e., such models require manual recalibration or some form of automated drift correction) [146,147,148,150]. Although the number of components in PCR and PLS models varies across studies, it has become common practice to select this number arbitrarily before cross-validation. Very few studies validate the optimal number of latent dimensions using cross-validation error and permutation testing [149,151]. The integration of these linear baseline models with an adaptive non-linear correction mechanism or physics-based constraint will increase their potential use in real-world deployments. Despite their interpretability, linear models struggle with complex matrix effects. Figure 6 demonstrates that while ‘Transparency’ is a key strength, ‘Non-Linearity Handling’ remains a significant weakness for standard regression techniques in complex biofluids.

## 6. Bayesian and Probabilistic Methods

Probabilistic machine learning provides a method to quantify uncertainties, incorporate priors into model formulations, and provide regularization, concepts significantly absent from traditional deterministic electrochemical models. Although less common in reporting results, probabilistic (Bayesian) methods can be very beneficial with respect to translating electrochemical models to the clinical setting and providing robustness in the presence of limited data.

### 6.1. Uncertainty-Aware Modeling and Probabilistic Classification

Naïve Bayes models provide rapid probability-based classification at low computational cost. The accuracy was 93.2% when using a Naïve Bayes model to classify the nickel ion concentrations from augmented CV data, which performed better than SVM and ANN in low sample conditions [153]. Gaussian Process (GP) regression has been used to calibrate clusters of electrochemical air quality sensors. GP not only predicts the concentration, but it also predicts a variance that quantifies the confidence of the prediction, as it varies across temperature and humidity gradients [154]. Probabilistic output is important for risk-aware environmental monitoring and clinical triage.

### 6.2. Bayesian Regularization for Small-Data Robustness

Bayesian regularization penalizes a complex configuration of weights in an ANN during the training process; it has the effect of preventing overfitting when there are insufficient experimental measurements. A BRNN incorporating prior distributions on all network weights used only 48 experimental points to predict sunset yellow FCF concentrations from DPV data, achieving R = 0.99 and MSE = 0.29. This demonstrated superior stability relative to standard back propagation [155]. By including prior distributions on network weights, Bayesian regularization allows one to obtain stable inference under conditions where cross-validation folds are limited. Despite their niche status, probabilistic methods offer unique advantages in uncertainty estimation. Table 5 describes the limited but critical applications of Bayesian approaches, noting computational and integration challenges. The potential of Bayesian and probabilistic approaches remains largely unexploited because of high computational requirements and poor hardware/software integration. A limitation in this area is that GP computation scales as a cubic function of the number of data points (n^3^), which limits applications using more than approximately 1000 data points for typical electrochemistry experiments without using some form of sparse approximation [154]. Most published studies do not include the full distribution of posteriors, nor credible intervals, with many probabilistic models reduced to mere point estimates [153,155].

Electrochemical prior distributions are very rare with respect to justification; default Gaussian or uniform priors will likely not accurately describe actual sensor noise distributions or variations due to fabrication. Utilizing variational inference and Monte Carlo Dropout along with electrochemically informed prior distributions will provide an opportunity to close the existing gap between probabilistic methodologies and practical biosensor development. Consequently, while probabilistic methods offer inherent uncertainty quantification, their adoption remains limited. As detailed in Section 9.3, this scarcity of UQ-aware models represents a significant barrier to regulatory approval for clinical electrochemical biosensors. Although probabilistic methods offer inherent uncertainty quantification, Figure 7 reveals that their adoption is hindered by ‘High Computational Requirements’ and ‘Limited Hardware Integration,’ resulting in few real-world deployments.

## 7. Instance-Based, Unsupervised and Symbolic Algorithms

Beyond mainstream supervised learning, instance-based, unsupervised, symbolic, and custom signal processing techniques were used to develop specialized methods to deal with specific electrochemical problems, such as lightweight classifications of pathogens and automated creation of datasets.

### 7.1. Pattern Recognition and Generative Data Augmentation

The k-Nearest Neighbor (KNN) algorithm provides a simple, distance-based prediction model with no need for parameters, which can be very effective on embedded systems. A KNN model had 100% accuracy in distinguishing opiates from cutting agents by using data collected from multi-electrode SWV arrays [156], and also provided dual classification/regression results of detecting ascorbic acid in sweat and fruit juices with an average of 96–97% accuracy [157]. Additionally, unsupervised clustering was integrated into generative models to provide automatic data generation: the k-means, Gaussian mixture models (GMM) and polynomial regression methods were used to create 100,000 simulated drug mixtures so that the trained MLP could have MAPE < 5% [158]. Likewise, GMM-assisted identquantnet algorithms were able to automatically identify and quantify multiple drugs from GMM-extracted CV features [159].

### 7.2. Symbolic Regression and Domain-Specific Signal Processing

Symbolic and domain-specific algorithms are particularly useful when both interpretability and physical consistency are required. The Sure Independence Screening and Sparsifying Operator (SISSO) was used to develop multivariable electrochemical descriptors for predicting cancer cell viability based on interactions at an SWV redox probe; SISSO showed 98–104% accuracy and provided reliable LC_50_ estimates [160]. For detecting pathogens, a custom Python pipeline applied the Fast Fourier Transform (FFT) to time series data obtained from potentiometry in smart blood culture bottles, which identified gram type with 95% accuracy and genus with 85% accuracy within hours of first signs of growth [161]. These studies show that there exist opportunities to create innovative algorithmic techniques that are based upon electrochemistry and can potentially provide higher levels of performance than generic machine learning/distributed neural networks in particular diagnostic applications. Beyond mainstream supervised learning, specialized algorithms address unique challenges in signal processing and data generation. Table 6 details the implementation specifics of KNN, FFT, SISSO, GMM, and custom pipelines, highlighting their niche strengths and generalizability limitations.

Limitations of alternative methods are based on scalability and generalizability. The KNN algorithm is limited by a degradation of performance as the number of features increases. This also limits the number of samples that can be stored for use with each model due to limitations associated with the need to store every sample used during the training phase [156,157]. Moreover, because unsupervised generative models cannot detect when a synthetic dataset contains a biased representation of the system, such bias may propagate through the entire pipeline. These would then provide misleading representations of the true state of the electrochemical system to subsequent classification models in the pipeline [158,159]. A second area where future research should concentrate is developing custom FFT/SISSO pipelines in ways that promote standardization. Because current pipelines are not standardized, such pipelines limit the ability to adopt these types of models across different laboratories and ultimately hinder their regulatory approval [160,161].

Collectively, Figure 8 demonstrates that while ‘Other Algorithms’ excel in niche applications requiring low computational overhead or physical interpretability, these models struggle with generalizability and standardization. This limits their widespread adoption compared to the more robust, albeit complex, neural network and ensemble frameworks.

### 7.3. Cross-Paradigm Comparative Synthesis

Synthesizing Section 2, Section 3, Section 4, Section 5, Section 6 and Section 7, no paradigm is universally superior; each is optimal within a specific data-regime/deployment window (Table 7). Deep learning is optimal for raw, high-dimensional, non-linear signals when large representative datasets exist; SVM and Bayesian methods for data-scarce regimes; tree ensembles for tabular mixed-type features; linear models for transparent, resource-constrained calibration; and symbolic/instance methods for interpretable or ultra-lightweight niches. The translational bottleneck is therefore not algorithmic but evidential: irrespective of paradigm, the Network Evidence Map shows 83.5% of studies lack uncertainty quantification, 85.7% restrict data access, and cross-batch validation is exceptional conditions under which even the best-matched inductive bias cannot guarantee clinical reliability.

## 8. Network Evidence Map: Quantitative Co-Occurrence of Methodological Strengths and Weaknesses

The key areas limiting the development of electrochemical sensors and their integration with artificial intelligence are deeply interconnected. For example, the lack of available data exacerbates the black-box nature of model interpretation, further amplifying the concerns regarding batch-to-batch variation. A systematic evidence map in the form of a systematic network is used to illustrate the interrelatedness of the areas and aspects of interest for future research. Network nodes represent coded labels, with edges denoting either joint probabilities or strong triplet participation, thereby identifying high-leverage intervention points. Thus, the 26 different strengths/weakness pairs are mapped, and the mechanistic associations between electrochemical sensor design and performance, the properties or type of inputs and outputs, AI model performance and translational barriers are quantitatively investigated.

**Figure 9:**
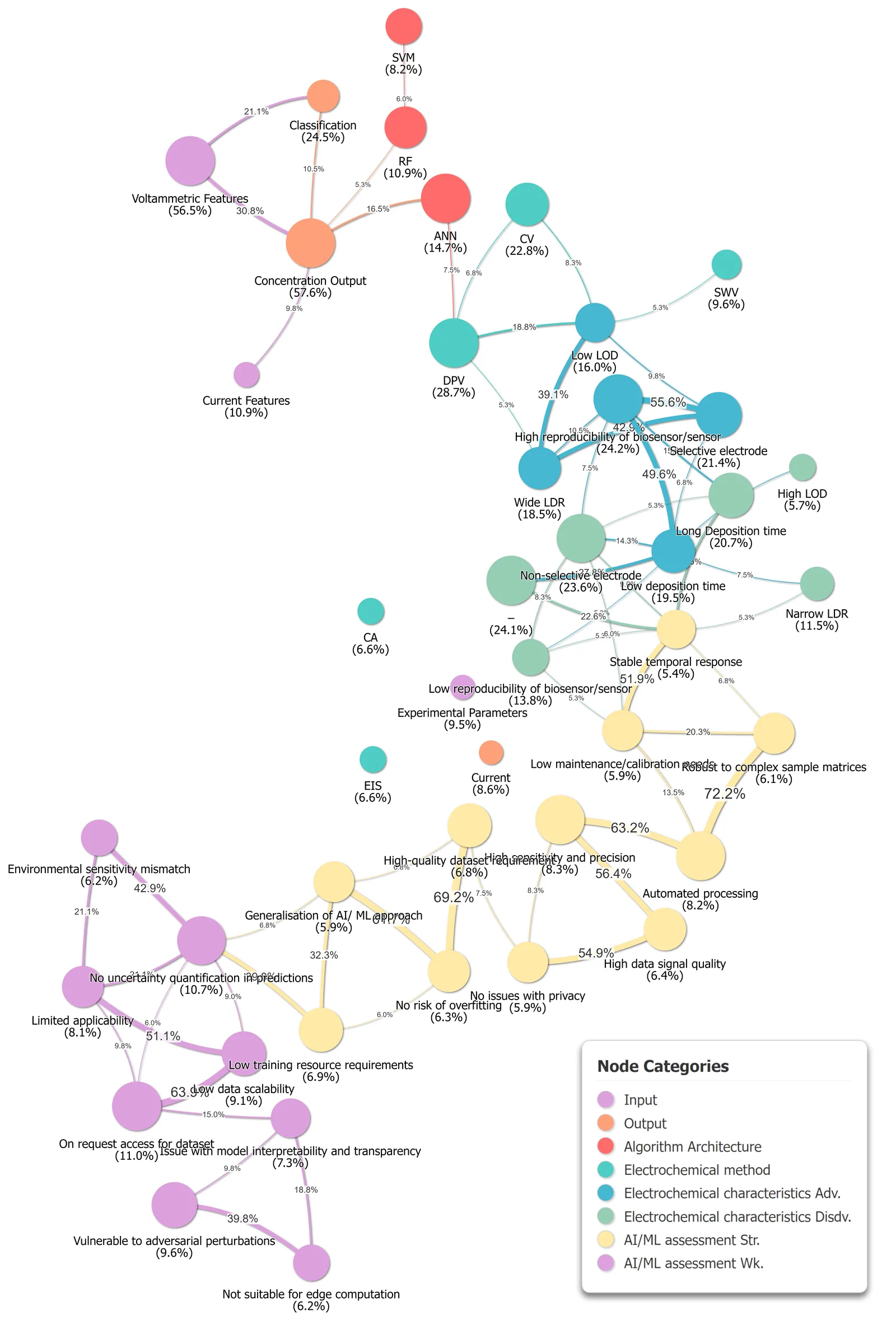
Joint-probability network map of methodological strengths, weaknesses, and translational barriers across all 133 reviewed studies (2016–2025). Node sizes are scaled relative to the frequency of code occurrence. Edges represent the co-occurrence probability between characteristics, filtered at ≥5% to reduce visual clutter. The network highlights strong associations between automated processing and matrix robustness, as well as the critical cluster linking data scarcity with restricted access and limited generalizability.

**Figure 10:**
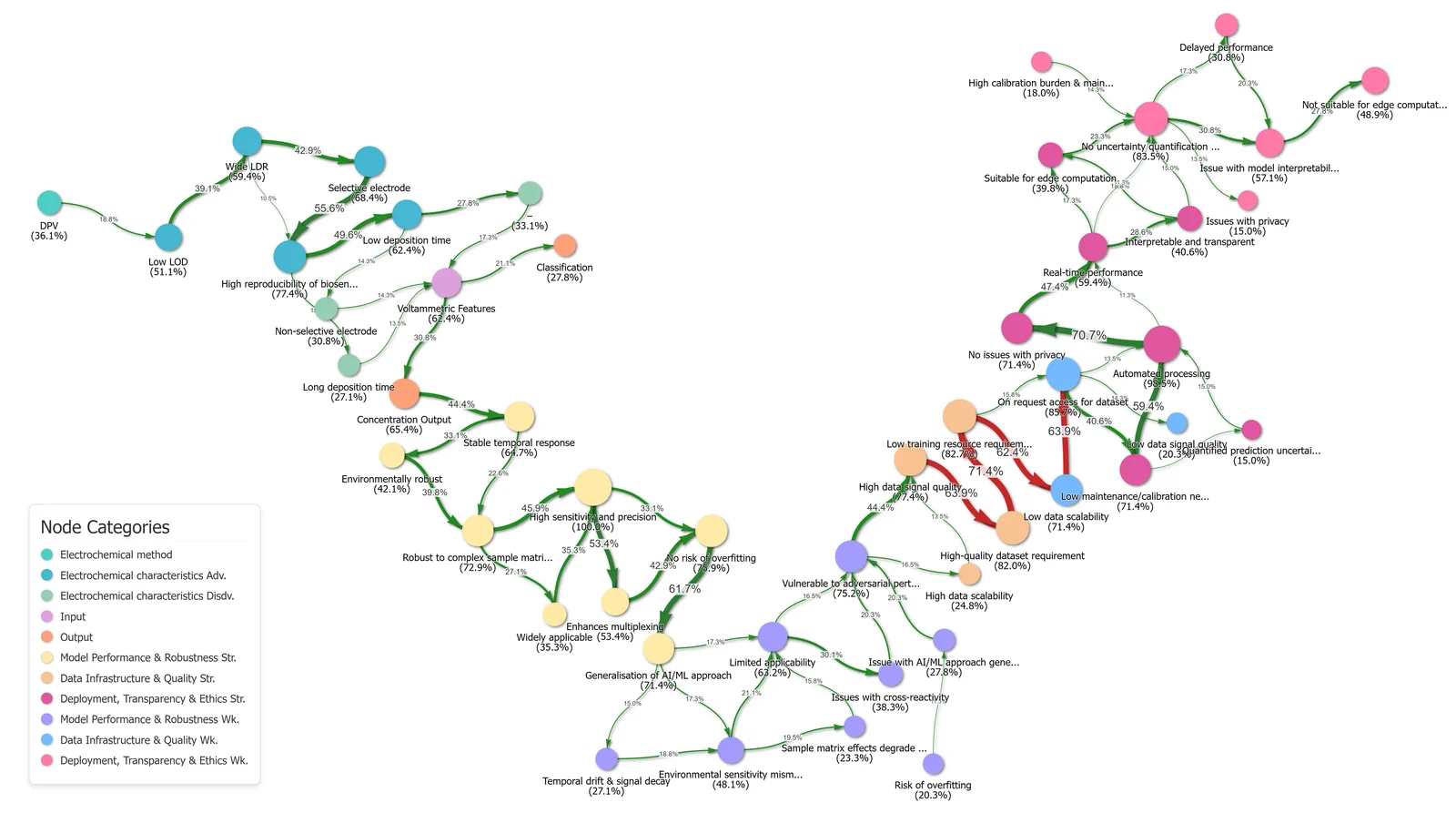
Path-based network analysis investigating contiguous doublets and triplets across the dataset. Node sizes correspond to overall frequency, while edge widths are proportional to the strength of connection (sub-path probability). Red edges indicate strong triplet participation (>0.5 probability), revealing strong associated pathways as the link between data quality, training efficiency, and model reliability. Green edges denote strong doublet associations. This visualization identifies high-leverage intervention points for overcoming translational barriers.

As shown in Figure 2, the same code may appear as an advantage/strength (⊕) or a disadvantage/weakness (⊖); this dual-pole design prevents code redundancy and reduces human error by simplifying the coding process. The codes are not presented in grouping order to prevent ordering bias; observations were pooled in a single spreadsheet. The contributing terminologies were normalized to reflect uniform usage across the data. Two summary networks were created using Python 3 (3.9.20), pyvis (0.3.2) for network visualization and selenium (4.35.0) with ChromeDriver (145) was used to capture high-resolution screenshots. All data wrangling steps were performed using pandas (2.3.2) and numpy (2.0.2), with pattern search and matching using regex (2024.4.28). For visualization, matplotlib (3.9.4) and seaborn (0.13.2) were employed. Co-occurrence was quantified using two complementary network approaches. (i) The joint-probability network (Figure 9) treats coded characteristics as unordered sets; edge weight represents the joint probability P(A∩B) = frequency of co-occurrence/total number of studies (N = 133), filtered at ≥5% to reduce visual clutter (65 of 658 unique edges retained). (ii) The contiguous doublets and triplets’ network (Figure 10) preserves sequential ordering within coded paths; sub-path probability represents the true prevalence of ordered code pairs/triplets across all studies, filtered at ≥10% (66 of 617 sub-paths retained). Edge widths in both networks are proportional to these probabilities (additional information in Supplementary Section S1.2).

### 8.1. Joint-Probability Analysis of Strengths and Weaknesses

In this analysis, the coded characteristics are treated as an unordered set of strings. The corresponding columns of advantages/strengths and disadvantages/weaknesses were exploded and mapped to full text descriptions. A flattened string structure representing all study characteristics is then computed for joint-probability analysis. Figure 9 shows the global co-occurrence of strengths and weaknesses. It provides a visual summary of claims that certain advantages (like automation) are universally linked to specific performance metrics, while disadvantages (like data scarcity) cluster together. The individual codes and characteristics are described as nodes with percentages, and the co-occurrence probability is represented as edges. The following frequently co-occurring trends are observed:

• As visualized in the dense clustering of nodes in Figure 9, studies demonstrating robustness to complex matrices strongly co-occur with automated processing (edge probability ≈ 72%). This illustrates that AI-driven automation is a primary driver for handling matrix effects. However, automation is a key benefit of AI/ML paradigms and automated processing code co-occurs frequently with high sensitivity and precision (≈62%), further confirming the benefit from AI/ML approaches.
• The requirement for high-quality datasets and their relationship to generalizability of the AI/ML paradigm and no risk of overfitting (≈62%) is evident. This is crucial for practical deployment of these sensors and biosensors.
• Conversely, reviewed studies reveal a distinct cluster linking ‘Low Data Scalability’ with ‘Restricted Data Access’ (≈64%), visually mapping the transparency crisis identified.
• The data signal quality and a lack of privacy issues co-occur (≈55%). These are interpretable in terms of application. The environmental, food safety or industrial applications often report high signal quality, and clinical applications require additional ethical considerations.
• Regarding sensor performance characteristics, the frequent co-occurrence of electrode selectivity and high reproducibility (≈56%) reflects the electrochemical advantage conferred by selective recognition mechanisms.
• Stable temporal response (as opposed to signal decay in complex matrices) frequently co-occurs with low calibration requirements (≈52%).

Finally, limited applicability co-occurs with low data scalability (≈51%). However, this indicates a utilitarian aspect of the devices, where single-purpose studies may limit investment in large-scale data collection.

### 8.2. Pathway Analysis: Contiguous Doublets and Triplets

The network described in Figure 10 has a modified counting approach. Here, the codes are subdivided into four initially planned categories: one group of electrochemical characteristics and three groups of AI/ML approach strengths and weaknesses. Semantic ordering is preserved as described in Figure 2. Doublets (2-nodes) and triplets (3-nodes) are extracted without combinatorial inflation. Therefore, apart from the benefit of joint probabilities, the contiguous paths provide additional quantitative trends revealing translational barriers. The key findings observed are as follows:

• The path analysis in Figure 10 identifies a high-probability triplet (red edges): ‘High Data Signal Quality’ leads to ‘High-Quality Dataset Requirement,’ which correlates with ‘Low Training Resource Requirement’ (56.4% participation). This suggests that improving initial signal quality is a high-leverage strategy for reducing computational burden.
• The triplet ‘Automation → Privacy → Real-Time Performance’ (47.4%) and the triplet ‘Low Maintenance → Automation → Privacy’ (44.4%) are essential for effective AI/ML workflow integration.
• Extending from the pattern related to low training resourse requirement, data scalability and limited access to the dataset (55.6%), a strong triplet participation indicates an unwillingness to provide open-source datasets, hindering the transparency of implementation.
• Sensors and biosensors with wide linear range demonstrate selectivity and high reproducibility (37.6%), indicating a synergistic interaction between fabrication approaches and performance. Often, the barriers of stability, cost and selectivity prevent effective commercialization, a previously observed trend in 10-year descriptive and quantitative analyses [162,163].

Critically, Figure 10 maps a barrier pathway where ‘Limited Applicability’ is moderately linked to ‘Low Data Scalability’ and ‘Restricted Access.’ This triplet indicates that the lack of open-source data is not just an administrative issue but a fundamental technical bottleneck preventing broad device applicability.

### 8.3. Syntheses of Co-Occurrence Patterns

Nearly 62% studies employ voltammetric features for concentration predication (joint probability: 30.8%), of the 133 reviewed studies, 111 studies (83.5%; 111/133) reported only deterministic point estimates without any accompanying measure of predictive uncertainty, while only 22 studies (16.5%; 22/133) met the positive criterion for uncertainty quantification (Code H). The operational definition of ‘uncertainty quantification’ used for coding was the explicit reporting of confidence intervals, prediction intervals, Bayesian credible intervals, conformal prediction bands, error bars, or variance estimates alongside the model’s point prediction. Studies reporting only aggregate performance metrics (R^2^, RMSE, MAE) without per-prediction uncertainty were coded as lacking UQ (Supplementary Table S1, Code H). Nearly 43% of these UQ-lacking studies also exhibited field-deployment barriers (likely environmental sensitivity mismatch). Several critical advantages and failure points are identified:

• The synthesis confirms that automated processing is a universal advantage. As seen in both the joint-probability map (Figure 9) and the path analysis (Figure 10), automation is tightly coupled with low training resource requirements and high reproducibility.
• The most critical finding of this analysis is the clustering of transparency failures. Figure 9 shows the high co-occurrence of on-request data access (85.7%; 114/133 studies), lack of uncertainty quantification (83.5%; 111/133 studies), and vulnerability to adversarial perturbations (75.2%; 100/133 studies). These three codes cluster together, revealing systemic issues with data transparency, model interpretability, scalability, and robustness. Only 22 studies (16.5%) employed any form of UQ (e.g., Bayesian methods, Monte Carlo Dropout, conformal prediction, or explicit confidence interval reporting). Figure 10 maps this as a persistent barrier path that prevents regulatory approval and independent validation.
• The network evidence suggests a potential cascading association, where poor data foundations correlate with complex ‘black-box’ models that are unsuitable for edge deployment (15.8%). This cascade is present only in the lower-probability paths of the network (not visible after filtering). While vulnerability to adversarial perturbations (100/133) and unsuitability for edge computation (65/133) are recurring themes.

Automation improves precision and reduces the operational burden of electrochemical devices. However, limited applicability also correlates with data scarcity and transparency; therefore, the path forward should emphasize these principles to qualify these sensors and biosensors for resource-constrained, rapid and self-adjusting smart devices. The reproducibility barrier while progressing remains to be addressed in complex matrices (sensitivity mismatch, degraded performance and limited applicability: 15.0%). The developed sensors and batch-to-batch effects should be validated initially before combining with automation paradigms, as one third of the studies are vulnerable to adversarial noise and issues with generalizability occur frequently with this characteristic (20.3%). Finally, sharing of data, demonstration of wide applicability and documentation of data processing protocols are key to standardization and wide integration of these devices.

## 9. Systemic Translational Barriers in AI-Enhanced Electrochemical Sensing

However, the high predictive accuracies reported across the reviewed studies do not necessarily translate into successful clinical or environmental use of these devices. Five major interrelated barriers must be overcome before significant translational progress can be achieved. To contextualize these barriers quantitatively, study-level statistics were computed from the coded dataset (n = 133). The median dataset size was ≈68 samples (IQR 24–210), with ≈72% of studies using <200 and ≈34% <50 samples. External validation was performed by only 11% of studies and cross-batch validation by ≈6%; ≈93% of studies reported no cross-batch reproducibility metrics, while the reproducibility-failure cluster in complex matrices (sensitivity mismatch, degraded performance, limited applicability) co-occurred in 15.0% of studies. Uncertainty quantification was absent in ≈83% of studies, of which ≈43% also exhibited a field-deployment barrier, and explainable AI was likewise negligible (a single ANN study employed SHAP/LIME). Regulatory-relevant transparency failures were pervasive: data access was restricted to on-request in ≈85%, ≈75% occurred in adversarial vulnerability, and 15.8% displayed the poor-data → black-box → edge-unsuitable cascade. Ultimately, ≈2% of studies simultaneously satisfied the four prerequisites for clinical translation: external validation, uncertainty quantification, cross-batch reproducibility testing, and edge-deployment demonstration.

### 9.1. The Data Scarcity and Accessibility Crisis

High-quality, large-scale training data are essential for robust machine learning; however, the current scarcity of accessible datasets poses a major concern for reliable electrochemical biosensing research. Many studies include datasets with very small sample sizes (typically fewer than 50 samples per study) [29,39,71]; such small datasets increase the risk of overfitting and inflate performance metrics such as R^2^, without ensuring generalization to new, unseen data. A systematic assessment of validation rigor across the 133 reviewed studies reveals heterogeneous practices that exacerbate this risk. Approximately 21 studies employed k-fold cross-validation (k = 5 or 10), 15 studies used leave-one-out cross-validation (LOOCV), 16 studies reported a single train/test split, and 81 studies did not explicitly describe their validation protocol. Nested cross-validation (separate inner loop for hyperparameter tuning and outer loop for performance estimation) was not identified in any of the 133 studies. The risk of data leakage, where information from the test set inadvertently influences model training, was not systematically reported in most studies. Further complicating the situation, several authors have used artificial, synthetic, buffer-spiked, or artificially increased datasets to train their models [53,158]. However, none of these approaches can replicate the random variation present in real-world samples nor the background interference often found in those samples. Crucially, while augmentation increases dataset size, it fails to capture complex matrix effects (e.g., protein fouling, ionic strength variations) inherent in undiluted biofluids, leading to models that perform well in silico but fail in clinical deployment. Unfortunately, most of the reviewed studies were limited in terms of transparency when it came to their use of data. In fact, the vast majority of the reviewed studies stated that their data was available upon request only [32,48,96,115]. The lack of openness related to sharing data does not allow other researchers to independently verify the results of previous studies. Additionally, this lack of openness limits the ability of others to develop a set of standard benchmarks for comparison among different models, which ultimately impacts the ability to build larger, open-source electrochemical databases that would be useful for developing common features via transfer learning.

### 9.2. The Real-World Gap: Matrix Effects and Environmental Drift

The most common drawback found in almost all the research examined is that many researchers have tested very sophisticated AI models with simple matrices. Frequently, AI models achieve >95% accuracy in PBS or artificial urine; however, their accuracy drops substantially in undiluted biological samples such as whole blood, serum or sweat, or even complex environmental samples [34,40,50]. This decrease in accuracy is primarily attributable to unmodeled environmental variables. Because electrochemical signals can fluctuate depending on the changes in pH, temperature, and ionic strength [39,100], some researchers have attempted to adjust for these variables by utilizing dual-path CNNs or multi-feature regression [38,56]; however, there has been a narrow focus placed on modeling the sensor as a dynamic unit that experiences long-term temporal drift, biofouling and electrode passivation while continuously monitoring over time [35,110].

### 9.3. The Black-Box Paradigm and Absence of Uncertainty Quantification

As modeling complexity increases (from linear regression through Deep Neural Networks and gradient-boosted ensembles), interpretability decreases correspondingly. Most studies that were reviewed viewed their AI models as being “opaque” or “black box,” and provided a single value representing the analyte’s concentration; however, the majority (over 83%) of the reviewed studies relied on deterministic point estimates without providing an associated measure of predictive confidence or uncertainty quantification (e.g., confidence intervals, Bayesian credible intervals, or conformal prediction bands) [32,48,118]. Specifically, 111 of 133 studies reported only deterministic point estimates, with no per-prediction confidence intervals, Bayesian credible intervals, conformal prediction bands, or variance estimates (operational definition per Supplementary Table S1, Code H). While Section 6 highlights emerging probabilistic approaches such as Gaussian Processes and Bayesian Neural Networks, these methods remain underutilized compared to standard deterministic architectures like CNNs and XGBoost. In both clinical diagnosis and environmental regulation, a single predictive point estimate, with no associated error estimate, has been found to be unacceptable on both legal and clinical grounds. Additionally, advanced techniques for interpreting the results of complex machine learning algorithms (SHAP/LIME) are very rarely used to identify the electrochemical feature(s) (for example, the particular peak potential(s), or shift(s) in the baseline) driving the model’s predictions [47,127,135]. Without such transparency, electrochemists cannot gain mechanistic insight into electrode-surface processes, and the information necessary for regulatory approval of in vitro diagnostic (IVD) devices remains lacking.

### 9.4. Hardware–Software Mismatches and Edge-Deployment Bottlenecks

While the pioneering works highlighted in Section 2.1.3 demonstrate that edge deployment of AI models (e.g., on ESP32 or TinyML platforms) is technically feasible [33,41], these instances remain exceptions rather than the rule. The majority of reviewed studies still rely on cloud-based or desktop-bound processing due to a persistent hardware–software mismatch. Complex architectures such as Deep CNNs, XGBoost ensembles, and LSTMs typically require computational resources (GPU acceleration, high RAM) that are incompatible with the low-power, memory-constrained microcontrollers used in portable POC devices [116,145].

To quantify the deployment landscape, we extracted the coding data for Code X (Edge Computability) and Code S (Real-Time Performance) across all 133 studies. Of these, only 7 to 10 studies demonstrated successful edge deployment on microcontrollers or embedded systems (e.g., ESP32 [41], ATmega328 [141], Intel Loihi [59], TinyML [33]), while ~21 studies were explicitly coded as unsuitable for edge computation due to cloud dependency, GPU requirements, or absence of deployment discussion. The remaining ~102–105 studies did not explicitly address deployment infrastructure, defaulting to desktop or workstation-based inference.

Among the edge-deployed studies, quantization was explicitly employed in 1–2 studies, with reported accuracy losses of 2–4% following 8-bit quantization (e.g., Dopamine detection: 98.1% at 32-bit vs. 96.01% at 8-bit [33]; glucose estimation: 97.8% on ESP32 [41]). In the context of clinical tolerance limits, a 2–4% accuracy loss may be acceptable for high-concentration screening applications. However, for low-abundance biomarker detection (e.g., cardiac troponins at ng/L levels [139], cancer biomarkers at pM concentrations [48]), even a 2% loss in classification sensitivity may result in missed diagnoses near critical clinical thresholds. Furthermore, quantization-induced errors are non-uniform across the concentration range, disproportionately affecting predictions near the limit of detection. We therefore recommend that future edge-deployment studies report: (i) pre- and post-quantization accuracy at clinically relevant concentration thresholds; (ii) per-class sensitivity/specificity changes; and (iii) uncertainty bounds on quantized predictions. The integration of uncertainty-aware quantization methods (e.g., quantization-aware training with Bayesian layers) represents a promising avenue to maintain clinical utility under hardware constraints.

### 9.5. Batch-to-Batch Variability and Reproducibility Crisis

The robustness of machine learning models depends entirely upon the hardware that produces the data used to train these models. Unlike traditional calibration, which may only require a new standard curve, AI models often require complete retraining or domain adaptation to account for batch-specific morphological changes, creating a significant computational and logistical burden for commercial scalability. In this regard, there exists an important but often neglected constraint, which is the inherent variability across batches in sensor manufacturing [35,117,139]. For example, drop-casting of electrodes introduces random variability; nanomaterial loading fluctuates between production runs; and the printing process itself introduces additional inconsistencies. As such, a model developed using data from a single, well-fabricated electrode batch will most certainly fail with respect to its ability to predict behavior within a new batch due to minute changes in the baseline current or peak potentials. Only very few of the existing studies have incorporated cross-batch validation or domain adaptation into their training methodologies to validate the model’s robustness with respect to manufacturing variance at the hardware level. This reproducibility deficit effectively confines many reported AI-enhanced electrochemical sensors to Technology Readiness Level (TRL) 3–4, corresponding to laboratory-scale validation under controlled conditions. Only a small number of demonstrations, typically those incorporating edge-deployable inference and a cross-batch testing approach TRL 6–7, where a system prototype is evaluated in an operationally relevant environment. This maturity gap underscores that batch-to-batch variability is not merely a fabrication inconvenience but one of the largest barriers preventing the transition from academic proof-of-concept to commercially viable, regulatory-compliant diagnostic devices.

To provide explicit evidence for the association between batch-to-batch variability and model opacity, we extracted the co-occurrence of Code D (Reproducibility) and Code T (Interpretability) from the Network Evidence Map coding data across all 133 studies. Among the 24 studies coded with low sensor reproducibility (Code D-negative), 13 simultaneously lacked any interpretability framework (SHAP, LIME, attention maps, or feature importance analysis), representing 54.2% of that subset. Among the 100 studies with high reproducibility (Code D-positive), 59 also lacked interpretability measures. The joint probability of Code D-negative co-occurring with Code T-negative in the Network Evidence Map is 9.8% (13/133). We note that Code T-negative (lack of interpretability) is pervasive across the field (≈58% of all studies), meaning that opacity is not uniquely associated with hardware variability. However, the co-occurrence data reveal that studies with unaddressed batch-to-batch variability are disproportionately those employing opaque models without explainability tools, creating a compounded barrier to regulatory approval. Notably, among the 76 studies coded as opaque (T-negative), 13 (17.1%) also exhibited low reproducibility, indicating that hardware variability and model opacity frequently co-occur as simultaneous translational barriers.

## 10. Future Perspectives: Pathways to Real-World Deployment

The Network Evidence Map demonstrates that the primary challenges facing AI-enhanced electrochemical biosensors have shifted from algorithmic development to the lack of reproducible, transparent, and reliable data. Thus, future research should be focused on addressing the above-mentioned strategic priorities as we transition from laboratory proof-of-concept to deploying the technology in real-world diagnostics and environmental applications.

### 10.1. Open-Source Repositories and Standardized Benchmarking

As indicated in the network analysis, there is a strong association between the limited capability of data scalability and “only on demand” data accessibility. It is imperative that the electrochemistry community create community-based, open access repositories for electrochemical data (similarly to PhysioNet for biomedical signals). In addition, creating standard formats for data (i.e., using standardized metadata for electrode dimensions, scanning rate, and matrix composition) will allow for robust benchmarking, support transfer learning, and eliminate the repetitive creation of small, isolated datasets.

### 10.2. UQ and XAI

The contiguous path analysis found that almost 85% of studies failed to report uncertainty in predictions, which is essential for clinical and regulatory acceptance. Therefore, future models should include uncertainty estimation frameworks (for example, Bayesian Neural Network (BNN), Monte Carlo Dropout (MC-Dropout), conformal prediction, etc.) that estimate confidence intervals along with point estimates. In addition, the “Black-Box” nature of complex models (such as Deep Convolutional Neural Networks and XGBoost) needs to be addressed by using XAI tools (like SHAP, LIME). Mapping model decisions back to specific electrochemical features (e.g., peak-potential shifts, baseline drifts) will build trust among electrochemists and regulatory agencies.

### 10.3. Physics-Informed Machine Learning (PIML)

Purely data-driven models require large datasets and typically fit training data well but generalize poorly to unseen conditions. The addition of domain expertise (i.e., Butler–Volmer kinetics, Randles–Ševčík equation, Randles circuit equivalent), in some form directly within the model architecture or loss function (Physics-Informed Neural Networks, PINNs), will greatly reduce the amount of data required for training. Electrochemical platforms can be fabricated with the goal of constraining ML as part of their initial fabrication process (i.e., minimize batch-to-batch variance from the start to reduce the need for frequent model retraining).

### 10.4. Hardware–Software Co-Design and Edge AI (TinyML)

The network map clearly shows that there is a significant trade-off in model complexity vs. edge deployability. Therefore, future development of sensors needs to follow an approach that has a co-designed hardware–software philosophy. This approach is more promising than the current design of heavy AI models on existing sensors. As noted in Section 10.3, fabrication processes should be designed to minimize batch-to-batch variance from the outset, thereby reducing the computational burden of drift compensation during edge inference. At the same time, advancements in Model Compression (Pruning, quantization, and distillation of knowledge) will allow the deployment of robust TinyML models directly on low-power microcontrollers, enabling truly standalone, real-time POC operation.

### 10.5. Federated Learning (FL) for Privacy-Preserving Clinical Translation

The need for data protection grows with the rise in personalized medicine and continuous monitoring as more use is made of electrochemical biosensors. FL enables decentralized model training across multiple clinical institutions without pooling raw patient data. Additionally, this allows for large amounts of high-quality data to be combined into models, which will increase the models’ ability to resist variations caused by biology. Furthermore, FL maintains compliance with local and federal data-protection regulations: the General Data Protection Regulation (GDPR) and the Health Insurance Portability and Accountability Act (HIPAA).

## 11. Conclusions

This review critically evaluated the integration of artificial intelligence and machine learning with electrochemical biosensing platforms, analyzing algorithmic architectures, application domains, and translational barriers across the literature from 2016 to 2025. The evidence demonstrates that AI/ML paradigms successfully overcome traditional electrochemical limitations. By resolving overlapping voltammetric peaks, compensating for environmental drift, and enabling multiplexed detection, machine learning has significantly enhanced sensor sensitivity, lowered limits of detection, and expanded the operational utility of electrochemical platforms for POC diagnostics and environmental monitoring.

However, the systematic Network Evidence Map unequivocally highlights that the field is constrained by systemic vulnerabilities. The most critical barriers identified are the pervasive reliance on small, non-representative datasets; the pervasive absence of uncertainty quantification identified herein; and the reproducibility crisis driven by batch-to-batch sensor variability. The strong network correlation between data scarcity, restricted data sharing, and limited model generalizability indicates that algorithmic sophistication cannot compensate for poor data foundations. Furthermore, the “black-box” nature of complex models without XAI frameworks remains a significant hurdle for clinical and regulatory acceptance.

In conclusion, the next decade of AI-enhanced electrochemical biosensing must shift its focus from isolated, proof-of-concept algorithmic demonstrations to the development of robust, transparent, and standardized ecosystems. The transformative potential of AI in electrochemical sensing is considerable; machine learning has already demonstrated the ability to resolve previously intractable analytical challenges such as overlapping redox peaks, multi-analyte deconvolution, and real-time drift compensation. However, realizing this potential in routine clinical and environmental practice demands that the community confront the technological, methodological, and regulatory challenges identified herein with the same rigor and urgency currently devoted to algorithmic innovation. The prerequisites identified in this review—community-driven open data, explainable and uncertainty-aware AI, physics-constrained learning, and hardware-aware model design—must transition from aspirational goals to enforceable community standards. Building upon the foundational prototypes, future work must transition from isolated single-analyte demonstrations to flexible, multi-target edge systems that maintain accuracy under quantization constraints. Only by addressing these foundational translation barriers can the field realize the full potential of intelligent biosensors for reliable, real-world clinical diagnostics, continuous health monitoring, and sustainable environmental protection.

## Figures and Tables

**Table 1:** Summary of characteristics and performance of ANN and variant paradigms in electrochemical biosensing in food, biological and environmental matrices.

Algorithm Architecture	Electrochemical Method	Sensor Material	Linear Range	LOD	Electrochemical Characteristics		AI/ML Model Characteristics			Matrix	Ref.
					Adv.	Disadv.	Input	Output	Performance		
ANN, LR	CV	OTE-Ag NPs	0.5–100 μM	0.102–4.86 μM	A, B, C, E	–	CV scan data features, LOOCV: a single observation from the original sample as for validation while remaining observations for training	Urea and fructose conc.	R^2^ = 0.58–0.86; RMSE = 0.43–2/92; MAE = 0.33–1.86	Aqueous Solutions	[69]
FNN	DPV, CA	MWCNT/MSQD/GCE	2–966 μM	0.6 μM	A, B, D	C, E	DPV scan features: 29 solutions (100 potential datapoints)	Dopamine conc.	R^2^ = 0.9879; MSE = 0.0012; MAE = 3.1 × 10^−5^; Huber Loss = 3.05 × 10^−5^	Human blood serum	[40]
ANN	DPV	CoWO_4_-activated CC	0.1–1.0 μM	0.024 μM	A, C, D, E	B	DPV features: current, charge, *N* = 182 data points, leave-one-out cross-validation	TBPA conc.	R^2^ = 0.9659; Low MSE/MAE/RMSE	PBS buffer, river water	[70]
BP-ANN	DPV	MIP-MXene/Fe-GO-MWCNT/AuNPs/SPE	0.001–1000 μM	0.00048 μM	A, B, C, D	E	DPV current (ΔI), Train:Test::60:40, *N* = 12 samples	PA conc.	R^2^ = 0.9998; RMSE = 0.0094; MAE = 0.0058; RPD = 37.3868	Pork samples	[29]
ANN	CA	Asparagine-functionalized GO	0–8 ppm	0.2 ppm	A, B, C, D, E	–	10 input features (*N* = 9385 data points): Sensor current at different time points and fabrication parameters, Train:Test::80:20	Free chlorine conc. (ppm)	MAE = 0.1603 ppm; Pearson r = 0.9950	Tap water	[35]
NN	CV	R6G/BIAI1/SPCE	1.125 × 10^3^–1.44 × 10^5^ ffu	1.61 × 10^4^ ffu	B, C, D, E	A	CV reduction curve data, Train: 10× stratified CV 9-folds, Test: 1 subset per fold (20 patients)	COVID-19 classification	Sensitivity: 90%; Specificity: 90%; Acc.: 90%	Human saliva	[47]
Perceptron (Adam)	CV	rGraphene-LIG	5 × 10^−9^–1 μg/L	0.032 μg/L	A, B, D, E	C	Voltammogram patterns: Train: NS; Test: 4 serum samples	Antibody detection	Acc.: 100%	Human serum	[46]
MLP	DPV	Green AgNPs/GCE	0.5–40 μM	0.126–0.147 μM	A, B, C, D, E	–	DPV current responses: Train:Test::75:25	BPA, PHE, CC conc.	R^2^: 99.19–99.81%	Water samples	[34]
Laplacian Eigenmaps, MLPNN	i-t Amp	Au, Pt, Ag, Graphite, Au-Pt alloy	–	–	D	C	Amperometry signals from sensor array: LOOCV; Train:Test::80:20	Beverage class	Acc.: 100%	Sabajon beverage	[44]
ANN-MLP	CV	MWCNT-Cu	1–250 μM	–	B, C, E	D	Experimental CV data: Current at 1.3 V and −0.455 V; Glucose conc. values	Glucose conc. estimation	Acc.: 96.7% (1.3 V); 97.8% (−0.455 V), MAE: 0.0138 (1.3 V); 0.0341 (−0.455 V)	NaOH solution	[41]
ANN	SWV	Unmodified SPCE	100–1000 μM	–	B, E	C, D	Raw SWV current curves: Train: 5492 curves (augmented); Test: 21 serum curves	Classification of sample type (4 classes)	Acc.: 98.1% (32-bit), 96.01% (8-bit)	CSF, PBS, human blood serum	[33]
Feedforward ANN	CV, DPV, CA	AuNPs/Poly(L-Arg)/RGO/GCE	30–1600 μM	–	B, D, E	C	Features from CV, DPV and CA: peak current, area); Train: 1440 sets (8 CAP conc. × 3 MNZ conc. × 20 repetitions × 3 methods), Train:Test::80:20	CAP conc.	R^2^: 0.95 (MNZ unknown), 0.99 (MNZ known), RMSE: 0.12 mM, MAE: 0.09 mM	Buffer, eye drops, honey, yogurt	[30]
ANN	DPV	C-ZIF-67/GCE	0.001–9 μM	0.0003 μM	A, B, C, D, E	–	DPV voltammograms	NA conc.	R^2^: 0.9928–0.9931, RMSE: 0.2710–0.2788, MAE: 0.1216–0.1262, R^2^ validation: 0.9954–0.9998, RMSECV: 0.5524–0.5583	PBS (pH 7.0)	[49]
ANN (3-6-1)	DPV	Pt-Pd NPs/COOH-Gr/MoS_2_	0.55–14.9 μM	0.18–0.44 μM	A, B, C, D, E	–	Experimental parameters (CCOOH-Gr, cycles, pH): *N* = 19 experiments, CCD design	Current response	R^2^ = 0.94, RMSE = 0.14	Meat products	[71]
ANN	DPV	pTS^−^/PPy MIPs/graphite-epoxy	0.5–2500 μM	1.1–320 μM	B, C, D, E	A	DPV scans (DCT coefficients): Train *N* = 33 samples from factorial research design; Test: *N* = 11 sample random subset	Conc. of PAC, AA, UA	R^2^ > 0.987, NRMSE = 0.020	Pharmaceuticals	[31]
ANN	LSV	P-PCMs-BP-AgNPs	0.2–125 μM	–	B, C, D, E	–	LSV scans: Train: Calibration data (0.2–125 μM, points not specified) with 5-fold CV; Test: Spiked human urine (3 levels, 3 replicates)	8-OHdG conc.	R^2^ = 0.9996	Human urine	[72]
ANN (1-5-1)	DPV	MoS2/MWCNTs porous nanohybrid with CMC	0.04–100 μM	0.0074 μM	A, B, C, D, E	–	DPV current responses: Train: calibration data; Test: real samples	CBZ conc.	R^2^ (Train): 0.9979 R^2^ (Test): 0.9985 RMSEP: 0.1533 RPD: 20.781	Tea and rice samples	[73]
ANN, SVM, Ridge, Elastic Net, DTR, Lasso, Bayesian Ridge, KNR	DPV	CB-GO/CP	40–2080 μM	–	B, C, D, E	–	13 DPV features with pH and temperature: *N* = 32 samples, Train:Test::70:30	TYR conc.	R^2^: 0.9828 (Ridge) RMSE: 5.4170 MAE: 4.4912 Recovery: 99.24%	Artificial urine	[38]
MLP	CA	Au UME	–	–	C, D, E	–	*N* > 50,000 collision events (≥1000 events per size) Train:Test::80:20 I_p_, Q, t_d_ parameters	NP size classification (30, 45, 60 nm)	Acc.: 100% (30 nm) >95% (45, 60 nm)	Alkaline solution	[37]
MLP, SVM	DPV	GO-SPCE	–	–	C, D	E	DPV fingerprints: Train: 70% of samples; Test: 15% of samples	*Lycoris* species classification	Acc.: 96.2%	Pollen extracts	[45]
ANN	DPV	CuO-Cdot-16.6	100–8500 μM	0.0014 μM	A, B, C, D	E	DPV data for glucose oxidation, *N* = 85 samples; LOOCV	Glucose conc.	MSE: 0.0141, MAE: 0.0142, RMSE: 0.0179, R^2^: 0.994	Blood serum	[50]
BPNN	CA	NiO/Pt, Ni(OH)2/Au, Ni(OH)2/Pt	0.25–20 mM	–	B, C, D, E	–	CA current data from three electrodes: Train: 96 groups of current data; Test: 24 groups with 15 groups for generalization	Conc. of glucose and lactate	Correlation coefficient: 0.9997, MRE: 0.01629 (glucose), 0.02414 (lactate)	Alkaline solution and serum samples	[74]
ANN (3-[30]-1)	DPV	AuNP/MWCNT/GCE	9.9 × 10^−12^–2.9 × 10^−11^ μM	9.8 × 10^−12^ μM	A, C, D, E	B	CV data (V, pH, SC): *N* = 7840; Train:Test::70:15	Current intensity	MSE: 0.007, R^2^: 0.9992	Sugar-free chewing gum	[75]
ANN	DPV, LSV, EIS	PVA-Cu^2+^-PEDOT	0.4–960 μM	0.06 μM	A, B, C, D	E	Sweat creatinine levels: Train: 50 datasets of no-stress and high-stress conditions; Test: Real-time monitored data	Heat-stress levels	–	Sweat samples	[42]
ANN	MPES	YSZ, LSC, ITO, Au, Pt	50–4000 ppm	–	B, C, E	D	Sensor signals from 4 electrodes: Train:Test::80:20; PCA pre-processing	Multi-class classification (3/5 gas sources) and CH_4_ conc.	Acc.: >98% Quantification error: <1.5% ppm	Air mixtures	[76]
ANN	LSV	GCE	0–55 ppm	–	D, E	B, C	3835 LSV curves (221-point raw current values or 442-point with 0 ppm baseline)	Regression (Free Chlorine Conc.)	MAE: 0.72–0.74 ppm (5-F CV), 2.48 ppm (Real); SMAPE: 10.1–11.4% (5-F CV), 25.2% (Real); R^2^: 0.9965–0.9968 (5-F CV), 0.9986 (Real)	0.01 M PBS, vegetable washing water	[36]
DT, MLP	CA	Pt-SPE/BGr/GOx, PDP	0.75–40 mM	0.078 mM	A, B, C	D, E	83 (calibration), 522 (glucose conc.); 80:20::Train:Test; BGr layers, time, analyte, current	Calibration– slope, y-intercept, limits; Glucose conc.	R^2^: 0.828–0.979	Cormay serum	[43]
ANN (ReLU, Linear)	DPV	MX-Au-C-1	0.1–7.5 nM	0.012 nM	A, B, C, D, E	–	DPV features (peak current, peak potential, relevant parameters)	ECM1 biomarker conc.	RMSE: 0.0073; MAE: 0.0042; R^2^: 0.999	PBS buffer, urine	[48]
ANN	LSV, DPV	Graphite	100–5000 mg/L	–	B, E	C, D	Voltammogram features—CAF: 701 training cases (6 features) CGA: 428 training cases (6 features)	CAF & CGA conc. (mg/L)	Acc.: 90.6–92.4%; RMSE: 4.48–10.20; MAE: 6.31–7.00	Coffee samples	[32]
ANN, LR, Ridge, Lasso	DPV	NF/CB–GO/SPCE	0–200 mM	–	B, C	D, E	32 DPV samples, 12 eigenvalues (70:30::Train:Test)	TYR Conc.	MAE, MSE, Loss, R2: 0.90–0.99	PBS (pH 7.2)	[39]
1D-CNN, ANN	DPV	AuNP	1–100 μM	0.62–1.38 μM	B, C, D, E	A	*N* = 180 DPV scans, 200-length 1D PCA SMOTE features from 180 DPV scans	Classification for Cd^2+^, Pb^2+^, Hg^2+^, Cu^2+^	Acc.: 97–100%; Pcs.: 0.99–1.00; Rec.: 0.99–1.00; F1: 0.99–1.00	Water samples	[51]
1D-CNN, ANN	Electrochemical sensing	SGX Sensortech, Spec Sensors	CO: 0.08–1.81 ppm O_3_: 0–107.8 ppb	–	–	C, D	24 h sequence of sensor readings: Multiple sensor recordings with environmental parameters; Train: 2 m.; Test: 8 m.	Calibrated CO and O_3_ conc.	CO: Mean RMSE = 0.2775 ppm; Mean R^2^ = 0.85, O_3_: Mean RMSE = 0.7454 ppb; Mean R^2^ = 0.38	Urban atmosphere	[56]
CNN (ResNet-50, SqueezeNet)	DPV	AgNPs/SSN	0.1–20 μM	0.01 μM	A, B, E	C, D	Train:Validation:Test::60:20:20, Dropout regularization, DPV scans	Sudan Red I conc.	R^2^: >0.96, RMSE: Less for Inception/ResNet	Food products	[53]
CNN	CV	Ti, Cu, Cd, W, Ag	–	–	D	C	CV curves from MEA: Train:Test::8:20, 20 × 446 input features from 1DConv Layer 1 to ReLU	Capsaicinoids conc.	RMSE: 5.407 μg/mL	Stewed duck wing brine	[54]
1D CNN	Corrosion potential variation	Zn/C, Al/C, or Cu/C electrodes	–	–	C, D, E	–	Voltage-time signals: *N* = 418 sample, Train:Test::80:20	Speech word categories	Accuracy: 99.07%	Human body (throat, wrist, chest, muscles, feet)	[60]
CNN, SNN	Conductometric, Potentiometric	Pt electrodes, ISFETs/polymeric membranes	6 × 10^−7^–10^−1^ M	–	B, D, E	C	16 × 9 time-series points (*N* = 21,197 data points); Train:Test::80:20	5 beverage classes	Acc.: 97.0% Latency: 5.6 ms Energy: 0.1 mJ/inf	Commercial beverages	[59]
1D-CNN	CV	Au/Pt electrodes, IL/DMSO	0–50 μL	9.3 μL	C, D, E	A, B,	CV scans: Voltammogram data; *N* = 108, 12 types, 9 cycles each; Fivefold cross-validation	VOC classification and methanol quantification	Acc.: 99.09% (VOC) 98.18% (methanol)	IL/DMSO solution	[52]
CNN	FL-EC	Etched CdTe/CdS QDs/sea urchin- FeOOH/Au	10–1000 μg/L	2.82 μg/L	A, B, C, D	E	FL spectrum and EC voltammetry data: Train:Test::44:4 samples	Conc. of Cd^2+^ and Pb^2+^	MAE: 0.2176–0.6002 μg/L; R^2^: 0.974–0.999; Recovery: 99.18–101.60%; RSD: 0.33–0.60%	Model solutions with complex background	[57]
CNN, RF, ANN, SVR	DPASV	g-C_3_N_4_/MWCNT/NH_2_-MIL-88(Fe)-AuNP NC	10 nM–15 μM	7.6–39.6 nM	A, B, C, D, E	–	100,000 voltammograms (1024 dp. each); single peak, binary, ternary, quaternary mixtures and exp. samples 15,000–60,000	Simultaneous conc. of Pb^2+^, Cd^2+^, Cu^2+^, Hg^2+^	RMSE: 0.032 nM R^2^: 0.9967 Acc.: 98.7% AUC: >0.99 all Rec.: 92–105% RSD: 2.9 ± 0.4%	Water (industrial, lake, municipal)	[55]
CNN, ANN	CV	TDDATFAB, DCE	–	–	C, E	D	2175 CV signals (Train: 1522, Validate: 435, Test: 217). CNN: Low-res CV images. ANN: 2 extracted (x,y) coord. baseline & plateau	Classification (Good/Faulty; Cd^2+^ vs. Cu^2+^) and Quantification (Conc. in mM)	CNN–Acc.: 95.37–99.15%; ANN–MAE: 0.0158–0.0127 mM; MSE: 4.09 × 10^−4^–2.61 × 10^−4^	KCl, DCE organic phase	[58]
LSTM, RF	ORP	Flexible carbon fiber	–	–	C, E	D	ORP and water quality parameters: *N* = 2376 pts; Train:Test::80:20	DO conc. prediction	RMSE: 1.08 mg/L R^2^: 0.80	Estuarine water	
ALSTM-FCN	CSWV	Multilayer epitaxial graphene/SiC	100–2000 ppb	70–380 ppb	B, E	A, C, D	CSWV scans: Train:Test::70:30	Analyte categories (e.g., MeP, BPA)	AUC > 0.90, Accuracy > 80% at ≥500 ppb	Seawater	[63]
RNN, LSTM	DPV, CV, OCP	GdSn-MnO_2_/PET/Carbon	10–620 nM	3 nM	A, C, D	B, E	CV features (voltage-current sequences), 3 doping ratios	Riboflavin detection, Optimal doping ratio	RMSE: 1.24 × 10^−4^ mA; Accuracy: 96.8%	Food samples, urine, PBS	[62]
LSTM, FCN, ALSTM, SVM, LDA, 1NN-DTW	CSWV	CSPE	–	50–2000 ppb	A, C, D, E	–	CSWV voltammograms (cathodic + anodic scans concatenated, 1002 data points); 4 datasets; 70:30::Train:Test	Heavy metals, pesticides/herbicides, industrial phenols, explosives classification	F1: 0.962–1.000; ROCAUC: 0.995–1.000	Seawater samples, buffer	[64]
ANN with custom SELU/LeakyReLU, XGBoost	CV, LSV	Cu/SPE/GCE	0.1–20 mM	0.05 mM	B, C, D, E	A	Pulse deposition param.: Voltage, ton, toff, cycles, substrate type; Train: 168 samples (100 electrodes × augmented data) Test: 10%	Peak current, peak potential, stability for NO^3−^ reduction	Cu/SPE and Cu/GCE MAE: Peak current: 8.82 μA, 8.97 μA; Peak potential: 0.01 V, 0.02 V; Stability: 0.19, 0.43	Tap water, river water	[67]
TwoWayANN	CV	SPE (DropSens DRP-11 L)	0–130 μM	–	B	C	Gaussian features (μ, σ^2^, A) from CV peaks: Train: 10 k simulated samples; Test: 2 k simulated CV and 15 real CV	ETO and MTX conc. (regression)	Acc.: 73.3–86.67%, MAPE: 18.68–42.07, MAE: 2.91–4.81, MSE: 14.09–32.28, OE: 80–99.38%	PBS with drugs	[68]
GA-ANN	SWV	POAP/DA/NPsTiO_2_	–	–	C, E	D	Accumulation time, pH, electrolyte conc.: *N* = 19 samples; Train:Test::70:30;	Current intensity prediction	R^2^ train: 0.99171 R^2^ test: 0.99808	Acetate solution	[65]
BP-ANN, GA	DPASV	BiFeO_3_/MX	0.001–1000 μg/L	0.0003 μg/L	A, B, C, D, E	–	Sensor parameters: Train: 12/16 OED groups; Test: 4/16 OED groups	Peak current	R^2^: 0.9897, RMSE: 4.5611, MAE: 3.0237	Lake water	[66]
ANN (IdentQuantNet)	CV	SPCE/CNTs/P450	10–180 μM	–	B	C, D	Gaussian features from CV scan redox peaks: Test: simulated and measured data (*N* = 20,000 simulated samples), 10 k data points per drug; Test: 20% simulated and measured	Drug classification and conc.	Identification Prec.: 100%; Rec.: 100%; F1: 100%, Quantification (MAE) CP: 6.67 µM; IFO: 4.88 µM	PBS buffer	[77]
Prototype DNN (soft voting)	OCP, EIS	Lipid membranes	–	–	C, D	E	OCP and EIS sensor data: Train: 1566 generated data; Test: 340 data points	Wine classification (6 types)	Accuracy: 95% (no errors), 90% (with errors)	Wine	[78]
ElasticNet, RF, KNN, SVM, DNN	CV	Au-sputtered Celgard^®^ membrane (platinized)	20–80 ppm	–	D	B, C, E	CV scans: Train and Test: 5-fold CV on pulses (12 cycles each) for: Pure NO_2_ (0, 20, 40, 80 ppm); Pure CO_2_ (0, 20, 40, 80 ppm); Combination of mixtures (20/60, 40/40 ppm)	NO_2_ and CO_2_ conc.	Avg. error (ppm): NO_2_: KNN = 0, RF = 0.18, DNN = 0.7; CO_2_: KNN = 5.4, RF = 6.5, DNN = 5.6; Mixture: KNN = 4.1, RF = 6.1, DNN = 4.9	Synthetic air	[79]

**Electrochemical Sensor Characteristics**. Advantages: (A) low LOD, (B) wide LDR, (C) selective electrode, (D) high reproducibility of sensor, (E) low deposition time. Disadvantages: (A) high LOD, (B) narrow LDR, (C) non-selective electrode, (D) low reproducibility of sensor, (E) high deposition time, Avg.—average, Conc.—concentration, Acc.—accuracy, Pcs.—precision, Rec.—recall.

**Table 2:** Summary of characteristics and performance of SVM implementation in electrochemical biosensing in food, biological and environmental matrices.

Algorithm Architecture	Electrochemical Method	Sensor Material	Linear Range	LOD	Electrochemical Characteristics		AI/ML Characteristics			Matrix	Ref.
					Adv.	Disadv.	Input	Output	Performance		
SVM-RBF	LSV	MIPs/GO	0.1–100 PFU/mL	0.1 PFU/mL	A, B, C, D	E	*N* = 360 data points from 28 patients; 2 features: absolute current change (ΔI) and relative change percentage (%ΔI/I_0_)	Classification (presence/absence) of ZIKV	Acc.: 91%; Pcs.: 89.7%	Urine	[80]
PCA, SVM	CV	BDD	–	–	D	C	*N* = 147 spectra (21 wines × 7 measurements) CV pre-processing in 100-segment windows, Testing: Embedded, PCA/SVM cross-validation	Wine variety classification and authentication	Acc.: 96%; Semi-supervised: 90% ± 10%; Supervised: 93% ± 8%; Unsupervised: 71% ± 17%	Wine and Coffee	[81]
SVM	EIS	Graphene/TiO_2_/SPCE	31.25–9000 ng/mL	10.56–14.05 ng/mL	A, B, C, D, E	–	*N* = 217 samples: Train:Test::70:30; RC components or PCs from EIS	Classification of DENV1 versus DENV2 IgG	Acc.: 94%; F1: 0.9388	PBS buffer	[82]
SVM	CV	GCE	–	–	D, E	C	Voltammetric fingerprints: Train: Tea extracts with reference TPC; Test: Commercial tea samples	Antioxidant activity prediction	Acc.: 93.5% R: 0.96 RMSE: 0.21 R^2^: >0.96	Tea extracts	[83]
PSO-SVM	Potentiometry	GCE/CRGN/NO_3_-ISM	100–100,000 μM	25 μM	B, D	A, C, E	Potential values: Train:Test::40:9 datasets	Nitrate conc.	MAE: 0.542 mM RMSE: 0.634 mM R^2^: 0.997	PM2.5 samples	[93]
SVM, NNET, GLMNET	CA	Electroactive biofilm/graphite anode/steel net cathode	1–10 mg/L	–	–	B, C, D, E	Current-time data features: Train:Test::20:3 samples	Toxicant conc. Mn^2+^, NO_2_^−^, TCH	RMSE: Mn^2+^: 0.21, NO_2_^−^: 0.23, TCH: 0.27; MAE: Mn^2+^: 0.19, NO_2_^−^: 0.21, TCH: 0.33	Water samples	[94]
SVM	Amperometric	YSZ, LSC, ITO, Au, Pt	–	–	B	C	9 out of 45 physics-based features; Train: 112 pts (89%); Test: 14 pts (11%)	Regression (I_P2_ current)	R^2^: 0.999 (train), 0.921 (test) LOM Gaussian R^2^: 0.985 (train), 0.984 (test) HOM linear	Exhaust gas	[95]
SVM	DPV	VP/COOH-MWCNTs-Nf(IP)	0.1–41.1 µM	5.8–6.4 nM	A, B, C, D, E	–	DPV response currents from 20 to 21 samples (15–20 training, 6–7 testing)	CLB and RAC conc.	Test: R^2^ = 0.9948–0.9972, RMSE = 0.6483–0.8551 µM, MAE = 0.5953–0.7281 µM, RPD = 7.093–17.462	Beef samples	[96]
SVM, PCA	SWV	CSPE	–	–	C, D, E	–	*N* = 549 training samples (2196 SWV scans), 27 variables from 4 pH conditions (pH 5, 7, 10/NQS, 12)	Class. (cocaine, heroin, ketamine, amphetamine, methamphetamine, MDMA + 24 adulterants/cutting agents)	Acc.: 91.8%; Sens.: 92.9%; Spec.: 100%; Selective Spec.: 89.7%; F1: 97.5%	Seized street samples, buffer	[84]
SVM (RBF Kernel)	CV	AQ/NR-GCE	3–2145 μM	–	B, C, D, E	–	160 voltammetric features, 18 spectroscopic features (150–1000 nm), 4 exp. features: time, temp, type, pH	Nitrate conc.	R^2^ = 0.97	Water, juice, plant extract, saliva	[97]
SVR-RBF	EIS	Ionic liquid	410–50,000 ppm	–	B, D, E	C	EIS spectra PCA features; Train: 6 experimental spectra, Test: 14 simulated spectra	CO_2_ conc.	Config1: R^2^ = 0.95; MAPE = 11.2%, Config2: R^2^ = 0.965, MAPE = 10%	Gaseous CO_2_	[98]
SVR	CV, DPV	MoS_2_-functionalized paper	0.01–100 μM	0.01 μM	A, B, C, D	E	CV and DPV oxidation currents: Train: 45 samples; Test: 5 samples	Dopamine conc.	Acc.: 98.87 ± 0.98% RMSE: 0.022 MAE: 0.016	Serum samples	[85]
SVR, MLR	Amperometric	SPEC Sensors assembly	0–10,000 ppb	0.1–1 ppb	A, B, E	C, D	NO_2_, T, RH sensor data: 3 measurement runs 5-fold CV; Train and Test: 3 measurement runs (1 h each); 5-fold cross-validation; Time-series (5 s windows)	Adapted NO_2_ conc.	SVR (Best); MSE: 0.0043–0.0109 Error reduction: 40–60%	Urban ambient air	[86]
ELM, ABC-LS-SVM	MLAPS	Pt, Au, Ni, Cr, W, Ag	–	–	C, D, E	–	Multi-frequency pulse voltammetry scans; Train: 40 samples, Test: 20 samples	Liquor purity classification and prediction	ELM Accuracy: 90.0%; ABC-LS-SVM R^2^: 0.9726; ABC-LS-SVM; MSE: 0.0233; ABC-LS-SVM MRE: 6.55%	Liquor samples	[99]
LS-SVM, ANN	SWV	HNT/BP-AgNPs	0.7–55 μM (Portable) 0.3–600 μM (Traditional)	0.3 μM (Portable) 0.1 μM (Traditional)	A, B, C, D, E	–	Current from voltammetric scans: *N* = 25-group CCD train samples; real samples test dataset	MH conc.	R^2^ ≅ 1 (LS-SVM/ANN) RMSE: Low (LS-SVM) MAE: Low (LS-SVM) RPD: 14.5992 (LS-SVM)	Sweet potato, carrot	[87]
BP-ANN-GA, LS-SVM/RBF/ELM	DPV	BP-AgNPs/MWCNTs-Nf(IP)/GCE	0.05–30.0 μM	0.0032 μM	A, B, C, D	E	DPV current responses: *N* = 24 train, 8 test samples using Kennard–Stone Train–Test splitting	CLB conc.	LS-SVM: R^2^ = 0.9977, RMSE = 0.0303, MAE = 0.0225, RPD = 18.74	Pork, pig serum	[89]
BP-ANN-GA, LS-SVM	DPV	AgNPs/MWCNTs/GO nanohybrid	0.2–122.2 μM	0.0139 μM	A, B, C, D, E	–	DPV peak currents: Train:Test::80:20	BN conc.	LS-SVM: R^2^ (train): 0.9997 R^2^ (test): 0.9963 RMSE: 0.6023 MAE: 0.3272 RPD: 10.20	Tea, cucumber	[90]
LS-SVM	DPV	MoSe_2_-BC	0.01–9.5 μM	0.002 μM	A, B, C, D	E	Peak current	HP conc.	R^2^ = 0.9983, RMSEP = 0.0999, MAEP = 0.0552, RPD = 13.82	Orange peel	[91]
LS-SVM, ANN	LSV	LIPG on polyimide	0.5–500 μM	0.16 μM	A, B, C, D, E	–	Peak currents from LSV; calibration set	SA conc.	R^2^: 0.99996 (cal), 0.99989 (pred) RMSE: 0.9566 (cal), 1.0088 (pred) MAE: 0.5226 (cal), 0.6736 (pred) RPD: 63.4683	Plant extracts	[92]

**Electrochemical Sensor Characteristics**. Advantages: (A) low LOD, (B) wide LDR, (C) selective electrode, (D) high reproducibility of sensor, (E) low deposition time. Disadvantages: (A) high LOD, (B) narrow LDR, (C) non-selective electrode, (D) low reproducibility of sensor, (E) high deposition time, Avg.—average, Conc.—concentration, Acc.—accuracy, Pcs.—precision, Rec.—recall, Cal.—calibration set, pred—prediction set, Sens.—sensitivity, Spec.—specificity.

**Table 4:** Summary of characteristics and performance of linear and multivariate regression in electrochemical sensing in food, biological and environmental matrices.

Algorithm Architecture	Electrochemical Method	Sensor Material	Linear Range	LOD	Electrochemical Characteristics		AI/ML Model Characteristics			Matrix	Ref.
					Adv.	Disadv.	Input	Output	Performance		
ULR	SWV	Carbon-coated 3D-PMNE	2–140 μM	0.129 μM	A, B, C, D, E	–	Sensing data: Peak current versus conc.	Buprenorphine conc.	R^2^ = 0.985	Artificial interstitial fluid	[146]
OLSR	Dual-CA	Au-NMEs/DNA probes/MCH-SAM	–	3.08 nM	A, C	D, E	Train: Unbound, non-ferrocene 1× PBS CA traces (0.02–0.38 ms); Test: Ferrocene-containing MP experiments (IFN-γ 100 pM–500 nM)	Conc. (via MSE) Drift-normalized current; Quant. of IFN-γ, Dopamine, DNA, Doxorubicin	MSE: LOD (3.08 nM to 1.53 nM); Sensitivity improvement (85% for IFN-γ); *p*-values (ANOVA): <0.0001, <0.001, <0.01	PBS, saliva (undiluted), serum	[148]
ULR	SWV	GR/SPCE	1–120 µM	0.13 µM	A, B, C, D, E	–	Peak current value from SWV spectra	Lidocaine conc.	R^2^: 0.98	Artificial interstitial fluid	[147]
PLSR	DPV	Cu wire	25–200 ppb	1 ppb	A, B, C, D	E	DPV current signatures (8 × 200), 8 conc. levels of Malathion	Malathion conc. quantification	Validation error: ≤5% (25–200 ppb); 10% (1 ppb), Nominal error: 5.0%	KCl Solution	[149]
PLSR	FFT-SWV	g-C_3_N_4_-CNT-GCE	1–20 µM	0.25–0.46 µM	A, C, D	B, E	17 training solutions FFT-SWV voltammograms	Simultaneous conc. MOR, MET, and UA	RMSECV: 0.1584–0.1951 µM; RMSEP: 0.1659–0.2035 µM; R^2^: 0.9545–0.9651	Urine samples	[150]
PLS Regression	Electrochemical deposition	Ag films on glass	0.01–10 g/L	0.01–0.1 g/L	C, D, E	A, B	SERS spectra: *N* = 150 (855 log-transformed features); Train:Test::80:20	HSA conc. (log scale)	R^2^ = 0.98, RMSE = 0.15	Aqueous solution	[151]
PCA, PCR	SWV	BDD	–	–	D, E	C	Electrochemical voltage-current spectral data from SWV (10 samples; 5 companies, 3 replicates)	CAF content quantification (mg/100 g)	Acc.: 93.88%; 80% quantified with 100% accuracy	Commercial coffee beverages	[152]

**Electrochemical Sensor Characteristics**. Advantages: (A) low LOD, (B) wide LDR, (C) selective electrode, (D) high reproducibility of sensor, (E) low deposition time. Disadvantages: (A) high LOD, (B) narrow LDR, (C) non-selective electrode, (D) low reproducibility of sensor, (E) high deposition time, Conc.—concentration, Quant.—quantification.

**Table 5:** Summary of characteristics and performance of Bayesian and probabilistic methods in electrochemical sensing in food and environmental matrices.

Algorithm Architecture	Electrochemical Method	Sensor Material	Linear Range	LOD	Electrochemical Characteristics		AI/ML Model Characteristics			Matrix	Ref.
					Adv.	Disadv.	Input	Output	Performance		
Naïve Bayes, ANN, SVM, DT	DPV	Ag_2_O-BiOBr-Nf composite	0–13.62 μM	–	B, D	C	CV scan features (after augmentation) Features: F1-F4, Train:Test::80:20, *N* = 117 samples	Multi-class classification of Ni^2+^ conc. (0, 0.2, 0.4, 0.6, 0.8 ppm)	Naïve Bayes Acc.: 93.2%	Aqueous	[153]
BRT, BLR, GP	Electrochemical sensor cluster	Alphasense sensors (NO_2_-B43F, O_x_-B431, CO-B4)	–	–	D, E	C	Sensor cluster data: NO_2_, O_x_, CO, VOCs, humidity, temperature; *N* = 8490 training points, 25,956 test points	NO_2_ and O_x_ conc.	RMSE for NO_2_: GP = 5.2 ppb, BRT = 7.2 ppb, BLR = 6.6 ppb	Ambient air	[154]
Bayesian regularized ANN	DPV	AgNPs-Co_3_O_4_/fCNTs	0.06–60.81 μM	0.0008 μM	A, B, C, D	E	Peak current from DPV: *N* = 48 points, Train:Test::70:30	Conc. of sunset yellow	R: 0.99, MSE: 0.29, Max Error: 1.32	Commercial beverages	[155]

**Electrochemical Sensor Characteristics**. Advantages: (A) low LOD, (B) wide LDR, (C) selective electrode, (D) high reproducibility of sensor, (E) low deposition time. Disadvantages: (A) high LOD, (B) narrow LDR, (C) non-selective electrode, (D) low reproducibility of sensor, (E) high deposition time, Conc.–concentration, Acc.—accuracy, Pcs.—precision, Rec.—recall.

**Table 6:** Summary of characteristics and performance of miscellaneous algorithmic paradigms in electrochemical sensing in food and biological matrices.

Algorithm Architecture	Electrochemical Method	Sensor Material	Linear Range	LOD	Electrochemical Characteristics		AI/ML Model Characteristics			Matrix	Ref.
					Adv.	Disadv.	Input	Output	Performance		
KNN	SWV	Graphite/SPCE, CoPc/SPCE, Pd/SPCE	–	–	D	C, E	SWV Voltammograms: 3 SPCEs × 20 samples; LOOCV	Multi-class classification (5 compounds)	Acc.: 100% Pcs.: 1.00 Rec.: 1.00 F1: 1.00	PBS buffer	[156]
KNN	CV, CA	Pd@Cu-BDC	1–6070 μM	0.83 μM	A, B, C, D	E	*N* = 74,000+ experimental (80:20::Train:Test); Redox potentials, current responses with Gaussian noise	Regression (AA conc.); Classification (sweat components & fruit sample origin)	Acc.: 96.67% (regression); Acc.: 97% (classification)	Artificial sweat, fruit juices	[157]
k-Means, GMM, PLR	CV	SPCE	5–30 μM	–	E	C, D	CV scans; Simulated dataset (1 M sets) generated from 3 measured conc. Groups, Test: 10 k sets (MTX: 40 μM fixed conc.)	ETO and MTX conc.	MAPE: ETO: 0.32%, MTX: 4.78%	PBS buffer and DMSO	[158]
MLP, ANN, DT, GMM	CV	SPCE	0–310 µM	–	A, B, D, E	C	Gaussian features (Mean, Variance, Amplitude, Peak) from CVs; Simulated (10,000 samples/drug) & Measured datasets	Class. and Conc. for ETO, MTX, IFO, CP, 5-FU	Ident.: Prec./Rec./F1: 100%; Quant.: MAPE: 5.03–21.55%; R^2^: 0.8364–0.9571	0.1 M PBS (pH 7.4)	[159]
SISSO	SWV	Au TFE	–	–	B, D	–	SWV currents at multiple potentials: MDA-MB-231 cells exposed to DOX (4 conc.), *N* = 3 samples per conc. (15 measurements) with five-fold CV; Test: Five-fold cross-validation	Cell viability (%)	Acc: 98–104%; R^2^: 0.994	Cell culture	[160]
FFT, Custom ML	OPC	PANI/IrO_x_/Carbon/PB	–	–	D, E	C	60 experiments (human blood) 14 species, 9 genera: FFT of potential/derivative signals; LOBO cross-validation	Gram and genus classification	Gram ID: 99% acc. (5.75 h) Genus ID: 85% acc. (7.8 h) No false positives/negatives	Human whole blood	[161]

**Electrochemical Sensor Characteristics.** Advantages: (A) low LOD, (B) wide LDR, (C) selective electrode, (D) high reproducibility of sensor, (E) low deposition time. Disadvantages: (A) high LOD, (B) narrow LDR, (C) non-selective electrode, (D) low reproducibility of sensor, (E) high deposition time, Avg.—average, Conc.—concentration, Acc.—accuracy, Pcs.—precision, Rec.—recall, Ident.—identification, Quant.—quantification.

**Table 7:** Cross-paradigm comparative assessment of AI/ML architectures in electrochemical sensing.

Paradigm	Performs Mechanistic Basis	Key Limitations	Computational Cost	POC/ Clinical Applicability	Robustness	Regulatory Readiness
ANN/CNN/RNN/DNN	Universal approximation; latent feature extraction from raw voltammograms	Data hunger; black-box; domain shift; over-optimistic augmented metrics	GPU training; edge inference after quantization (5.6 ms; 0.1 mJ) with 2–4% accuracy loss	High analytical precision; quantization penalty may breach clinical tolerance for trace biomarkers	Low–moderate (biofluid collapse; 75.2% adversarial vulnerability)	Lowest (UQ/XAI ≈ absent; ~82% no UQ)
SVM/LS-SVM	Structural risk minimization → small-data generalization	O(n^2^–n^3^); ~10^4^ cap; empirical kernels; no calibrated probabilities	Moderate training; tiny inference footprint (linear kernel on ATmega328)	Strong for portable agro-food/environmental screening	Moderate	Low–moderate (needs Platt/GP variants)
DT/RF/GB/XGBoost	Variance reduction; mixed-type inputs; partial interpretability	Miss latent voltammetric structure; GPU > 10^3^ samples; global-only interpretability	GPU training; moderate inference	Good screening; insufficient for high-precision clinical without prediction intervals	Moderate–low (inter-site R^2^ 0.31–0.64; cross-batch gaps)	Moderate
ULR/OLS/PLS/PCR	Transparent; <1 ms; statistical calibration	Fails under fouling/saturation non-linearity; unvalidated component counts	Negligible	Ideal wearable/microneedle POC	Poor in complex biofluids	High defensibility, limited precision
Bayesian/GP/BRNN	Native UQ; prior regularization under scarcity	O(n^3^); often published as point estimates	High training	Risk-aware triage; needs sparse inference	Good with physics-informed priors	Highest (native UQ matches IVDR/FDA)
KNN/SISSO/FFT/GMM	Minimal overhead or closed-form interpretability	Memory-bound; simulation-bias propagation; non-standardized	Low inference (KNN stores all data)	Niche embedded/pathogen ID	Low generalizability; small cohorts	Variable (SISSO high per parameter)

## Data Availability

The data are self-contained within the review. Further inquiries can be directed to the corresponding author.

## Supplementary Materials

The following supporting information can be downloaded at: https://www.mdpi.com/article/10.3390/bios16090470/s1, Section S1: materials and methods; Section S1.1: development and implementation of the coding scheme; Section S1.2: systematic evidence maps methodology; Section S1.2.1: joint-probability network; Section S1.2.2: contiguous doublets and triplets in paths approach; Section S1.3. Supplementary Results for Contiguous Doublets and Triplets Analysis; Figure S1: flow diagram of study counts during retrieval, screening, appraisal and analysis stages of the review; Table S1: coding manual for positive and negative coded characteristics employed in the review (References [29,30,31,33,35,36,39,46,47,51,53,54,56,60,62,65,67,68,69,73,77,78,80,81,85,93,99,101,102,103,105,109,116,121,123,124,126,127,128,130,141,145,146,149,153,157,158,159,162,163,164,165,166,167,168,169,170,171,172,173,174,175,176,177,178,179,180,181,182,183,184,185,186,187,188,189,190,191,192,193,194,195,196,197,198,199,200,201,202,203,204,205,206] are cited in the supplementary File.

## Abbreviations

The following abbreviations are used in this manuscript:

1-D	One-Dimensional
3-APBA	3-Aminophenylboronic Acid
5-F CV	5-Fold Cross-Validation
5-FU	5-Fluorouracil
8-OHdG	8-hydroxy-20-deoxyguanosine
AA	Ascorbic Acid (Vitamin C)
ABC-LSSVM	Artificial Bee Colony–Least-Squares Support Vector Machine
ABTS	2,2′-Azinobis-(3-ethylbenzthiazoline-6-sulphonate)
Acc.	Accuracy
AdaBoost	Adaptive Boosting (Classifier)
AgNPs	Silver Nanoparticles
AI	Artificial Intelligence
ALSTM-FCNs	Attention LSTM-Fully Convolutional Networks
ANN	Artificial Neural Network
ANOVA	Analysis of Variance
Anti-ECM1	Antibodies against ECM1
AQ	Anthraquinone-2-sulfonate
AUC	Area Under the Curve (ROC)
AuNF	Gold Nanoflowers
AuNPs	Gold Nanoparticles
BA	Benomyl
BDD	Boron-Doped Diamond
BES	Bio-Electrochemical Sensor
Bi/GCE	Bismuth Film-Modified Glassy Carbon Electrode
BIAI1	Bio-Inspired AI-Designed Peptide
BLR	Boosted Linear Regression
BN	Benomyl
BNN	Bayesian Neural Network
BP	Back Propagation
BPA	Bisphenol A
BP-ANN	Back Propagation Artificial Neural Network
BRNN	Bayesian Regularization Neural Network
BRTs	Boosted Regression Trees
CA	Chronoamperometry
CA 15-3	Cancer Antigen 15-3
CAF	Caffeine
CAP	Chloramphenicol
CatBoost	Categorical Boosting
CB	Carbon Black (Vulcan xc-72)
CBD	Cannabidiol
CBZ	Carbamazepine
CC	Catechol
CCD	Central Composite Design
Cdot	Carbon Quantum Dot
CGA	Chlorogenic Acid
CHI	Chitosan
CIP	Ciprofloxacin
CLB	Clenbuterol
CMC	Carboxymethyl Cellulose
CNN	Convolutional Neural Network
COOH-MWCNTs	Carboxylated Multi-Walled Carbon Nanotubes
CP	Cyclophosphamide
Cross val.	Cross-Validation
CSTB	Cystatin B
CSWV	Cyclic Square-Wave Voltammetry
cTnI	Cardiac Troponin-I
cTnT	Cardiac Troponin-T
CV	Cyclic Voltammetry
DA	Dopamine
DCE	Dichloroethane
DEHP	Di(2-ethylhexyl) phthalate
DENV1/DENV2	Dengue Virus Serotype 1/2
DMSO	Dimethyl Sulfoxide
DNA	Deoxyribonucleic Acid
DO	Dissolved Oxygen
DOP	Dopamine
DOX	Doxorubicin
DPASV	Differential Pulse Anodic Stripping Voltammetry
DPV	Differential Pulse Voltammetry
DT	Decision Tree
DTW	Dynamic Time Warping
Dual-CA	Dual-Chronoamperometry
EBFC	Enzyme Biofuel Cell
ECL	Electrochemiluminescence
ECM1	Extracellular Matrix Protein-1
EIS	Electrochemical Impedance Spectroscopy
ELM	Extreme Learning Machine
EP	Epinephrine
EschC	*Escherichia coli*
ETO	Etoposide
e-tongue	Electrochemical tongue
F1	F1-Score (harmonic mean of precision and recall)
F2	F2-Measure
FCN	Fully Convolutional Networks
FDA	United States Food and Drug Administration
FFT	Fast Fourier Transform
FFT-SWV	Fast Fourier Transform Square-Wave Voltammetry
FL	Federated Learning
FTIR	Fourier Transform Infrared Spectroscopy
GA	Genetic Algorithm
GBA	Gradient Boosting Algorithm
GB	Gradient Boosting
GBT	Gradient-Boosted Trees
g-C_3_N_4_-CNT-GCE	Carbon Nitride–Carbon Nanotube-Modified Glassy Carbon Electrode
GCE	Glassy Carbon Electrode
GDPR	General Data Protection Regulation
GDY	Graphdiyne
GLM	Generalized Linear Model
GMM	Gaussian Mixture Model
GO	Graphene Oxide
GOx	Glucose Oxidase
GP	Gaussian Process
GPR	Gaussian Process Regression
GPU	Graphics Processing Unit
HIPAA	Health Insurance Portability and Accountability Act
His-pHEG	Histidine-Functionalized poly(2-hydroxyethyl methacry-late-co-ethylene glycol dimethacrylate)
HOM	High-Order Model
HP	Hespertine
HRP	Horseradish Peroxidase
HSA	Human Serum Albumin
HVA	Homovanillic Acid
ID	Identification
IDEs	Interdigitated Electrodes
IFN-γ	Interferon-gamma
IFO	Ifosfamide
IgG	Immunoglobulin G
IoT	Internet-of-Things
ISF	Interstitial Fluid
ITIES	Interface between Two Immiscible Electrolyte Solutions
ITO	Indium Tin Oxide
IVD	In Vitro Diagnostic
IVDR	In Vitro Diagnostic Regulation
KNNs	K-Nearest Neighbors
LAACV	Large Amplitude Alternating Current Voltammetry
LASSO	Least Absolute Shrinkage and Selection Operator
LDA	Linear Discriminant Analysis
LDR	Linear Dynamic Range
LIG	Laser-Induced Graphene
LightGBM	Light Gradient Boosting Machine
LIME	Local Interpretable Model-Agnostic Explanations
LOD	Limit of Detection
LOM	Low-Order Model
LOOCV	Leave-One-Out Cross-Validation
LOQ	Limit of Quantitation
LR	Linear Regression
LSBoost	Least-Squares Boosting
LSC	Lanthanum Strontium Cobaltite
LS-SVM	Least-Squares Support Vector Machine
LSTM	Long Short-Term Memory
LSV	Linear Sweep Voltammetry
LTA4H	Leukotriene A4 Hydrolase
MAE	Mean Absolute Error
MAPE	Mean Absolute Percentage Error
MC-Dropout	Monte Carlo Dropout
MCH	Mercaptohexanol
MDA-MB-231	Human Breast Adenocarcinoma Cell Line
MDMA	3,4-Methylenedioxymethamphetamine
MeP	Methylparaben
MET	Methadone
MH	Maleic Hydrazide
MIP	Molecularly Imprinted Polymer
ML	Machine Learning
MLP	Multilayer Perceptron
MLR	Multiple Linear Regression
MNB	Multinomial Naïve Bayes
MNR	Methadone
MNZ	Metronidazole
MOF	Metal–Organic Framework
MOR	Morphine
MPA	Mycophenolic Acid
MRE	Mean Relative Error
MSE	Mean Squared Error
MTX	Methotrexate
MUC-1	Mucin 1
MWCNT	Multi-Walled Carbon Nanotube(s)
MX	MXene
N+	Metastatic (with lymph node metastasis)
N0	Non-Metastatic (without lymph node metastasis)
NA	Niclosamide
NB	Naïve Bayes
NCs	Nanocomposites
NEP	Norepinephrine
Nf	Nafion
NH_2_-MIL-88(Fe)	Amino-Functionalized Metal–Organic Framework (Iron-Based)
NME	Nanostructured Microelectrode
NPs	Nanoparticles
NR	Nitrate Reductase
NQS	1,2-naphthoquinone-4-sulfonic acid sodium salt
OCP	Open-Circuit Potential
OLS	Ordinary Least Squares
ORP	Oxidation-Reduction Potential
OSCC	Oral Squamous Cell Carcinoma
OTE	Oolong Tea-Derived
PA	Palmitic Acid
PAC	Paracetamol
PANI	Polyaniline
PB	Prussian Blue
PBS	Phosphate-Buffered Saline
PCA	Principal Component Analysis
PCA-SVM	Principal Component Analysis–Support Vector Machine
PCR	Principal Component Regression
Pcs.	Precision
Pd@Cu-BDC	Palladium-Incorporated Copper Benzene-1,4-Dicarboxylate Metal–Organic Framework
PEDOT	Poly(3,4-ethylenedioxythiophene)
PET	Polyethylene Terephthalate
PHE	Phenol
PIML	Physics-Informed Machine Learning
PINNs	Physics-Informed Neural Networks
PLS	Partial Least Squares
PMNE	Printed Microneedle Electrode
POAP	Poly(o-aminophenol)
POC	Point-of-Care
P-PCMs	Phosphorus-Doped Porous Carbon Microspheres
Prec.	Precision
PSO	Particle Swarm Optimization
PVA	Polyvinyl Alcohol
QD	Quantum Dot
Quant.	Quantification
R^2^	Coefficient of Determination (R-squared)
R^2^v	Determination Coefficient of the Validation Set
RAC	Ractopamine
RBF	Radial Basis Function
Rec.	Recall
ReLU	Rectified Linear Unit
RF	Random Forest
RFE	Recursive Feature Elimination
RGO	Reduced Graphene Oxide
Ridge	Ridge Regression
RMSE	Root-Mean-Squared Error
RMSECV	Root-Mean-Squared Error of Cross-Validation
RMSEP	Root-Mean-Squared Error of Prediction
RMSEV	Root-Mean-Squared Error of the Validation Set
RNN	Recurrent Neural Network
ROCAUC	Receiver Operating Characteristic Area Under the Curve
RPD	Relative Prediction Deviation
RRMSE	Relative Root-Mean-Squared Error
RSD	Relative Standard Deviation
SA	Salicylic Acid
SAM	Self-Assembled Monolayer
SARS-Cov-2	Severe Acute Respiratory Syndrome Coronavirus-2
SCV	Staircase Cyclic Voltammetry
Sens.	Sensitivity
SER	Serotonin
SERS	Surface-Enhanced Raman Scattering
SG	Savitzky–Golay
SGD	Stochastic Gradient Descent
SHAP	SHapley Additive exPlanations
SISSO	Sure Independence Screening and Sparsifying Operator
SMAPE	Symmetric Mean Absolute Percentage Error
SoCs	System on Chip(s)
SPCE	Screen-Printed Carbon Electrode(s)
SPE	Screen-Printed Electrode(s)
Spec.	Specificity
*StaphA*	*Staphylococcus aureus*
SVC	Support Vector Classification
SVM	Support Vector Machine
SVR	Support Vector Regression
SWASV	Square-Wave Anodic Stripping Voltammetry
SWV	Square-Wave Voltammetry
TBPA	Tetrabromobisphenol A
TCH	Tetracycline Hydrochloride
TDDATFAB	Tetradodecylammonium Tetrakis (Pentafluorophenyl) Borate
TEAC	Trolox Equivalent Antioxidant Capacity
THC	Tetrahydrocannabinol
TRL	Technology Readiness Level
Tyr	Immobilized Tyrosinase
TYR	Tyrosine
UA	Uric Acid
ULR	Univariate Linear Regression
UME	Ultramicroelectrode
UQ	Uncertainty Quantification
*VibrioP*	*Vibrio parahaemolyticus*
VOC	Volatile Organic Compound
VP	Violet Phosphorene
XAI	Explainable AI
XGBoost	Extreme Gradient Boosting
YOLOv5x	You Only Look Once version 5x (Object Detection Algorithm)
YSZ	Yttria-Stabilized Zirconia
ZIF-67	Zeolitic Imidazolate Framework-67
ZIF-8	Zinc Imidazole Framework-8
ZIKV	Zika Virus
β-LG	Beta-lactoglobulin
