# Supplementary material for: Artificial Intelligence in Electrochemical Sensing: A Network Evidence Map of Translational Barriers and Pathways to Point-of-Care Deployment
Source: Biosensors (Basel). 2026 Aug 27;16(9):470. doi: 10.3390/bios16090470 (PMC13604714; doi:10.3390/bios16090470)
Supplement: Supplementary file 1 [file biosensors-16-00470-s001.zip › biosensors-4469692-supplementary.pdf]

Supplementary Materials

Artificial Intelligence in Electrochemical Sensing: A Network Evidence Map of Translational Barriers and Pathways to Point-of-Care Deployment

Muhammad Saqib [1,2,*], Elena I. Korotkova [1], Kunquan Li [3], Neda Firoz [4], Mrinal Vashisth [4], Amrit L. Hui [4], Olga I. Lipskikh [1] and Pradip Kumar Kar [5]

1 Chemical Engineering Division, School of Earth Sciences and Engineering, National Research Tomsk Polytechnic University, 30 Lenin Avenue, 634050 Tomsk, Russia; eikor@tpu.ru (E.I.K.)
2 UNESCO Laboratory of Environmental Electrochemistry, Department of Analytical Chemistry, Faculty of Science, Charles University, Hlavova 8/2030, CZ 128 43 Prague, Czech Republic
3 College of Engineering, Nanjing Agricultural University, Nanjing 210031, China; kqlee@njau.edu.cn
4 National Research Tomsk State University, 36 Lenin Avenue, 634050 Tomsk, Russia
5 Parasitology Laboratory, Department of Zoology, Cooch Behar Panchanan Barma University, Vivekananda Street, Cooch Behar 736101, India; karpradip@gmail.com
* Correspondence: saqib@tpu.ru

S1. Materials and Methods

S1.1. Development and Implementation of the Coding Scheme

The coding scheme was brainstormed in an open-ended manner with refinements to avoid redundancies and ensure proper phrasings. The initial thematic grouping is an expert-judgement step inherent to coding-scheme development; it was consolidated after establishing face validity on a subset of examined studies by a single validator (subject-matter expert) in November 2025 and fixed before code registration, after which all coding and analytical steps were rule-based and quantitative. Subsequently, the process of coding was undertaken by a single coder in two rounds over a duration of seven months (November 2025 to June 2026), with each round lasting roughly three months with a short washout period in between the rounds. To ensure consistency, the phrasing of each code was finalized before code registration along with the operationalized definition and set inclusion/ exclusion criteria. During registration, the codes for AI/ML strengths and weaknesses (F–Z) were intermixed (not registered in their thematic grouping order) to prevent ordering bias [1]. The mean Jaccard Similarity across two rounds in the coding process of positive and negative characteristics was 100% and 99.8% respectively. To identify edge cases and coding inconsistencies, checks of the data structure were performed using the Python programming language. This step ensured that the codes are either advantage/ strength or disadvantage/ weakness with zero logical violations (overlaps, duplication or improper data formatting).

The coding scheme framework for the evaluation of AI/ML paradigms in electrochemical sensing was conceptualized by considering common issues or limitations, advancements in the field of AI and ML approaches and consulting the regulatory guidelines and observations from represented studies during the face validation step. The electrochemical characteristics (codes A–E) are attributed to IUPAC and electroanalytical chemistry guidelines such as the statistical boundaries of Limit of Detection, Linear Dynamic Range and selectivity as baselines for validation of sensor materials prior to the application of AI/ ML approaches [2–7]. The model performance and robustness codes (F, I, J, L, M, R, V and W) are refined in consideration of domain shifts and reproducibility issues due to fouling and matrix effects. In this regard, ML models trained on simple matrices may fail during real sample analyses, or such models are implemented to compensate for drifts [8–20]. Adversarial perturbations (code Z) are a represented issue in deep learning applications on continuous streamlined data, such as from IoT applications

[21]. In this regard, approaches such as domain adaptation are useful to improve model accuracy [22,23]. Furthermore, the data infrastructure and quality codes (N, O, P, U and Y) are related to Findable, Accessible, Interoperable and Reusable data principles [24], and advancements such as federated learning [25], which are relevant for computational analytical chemistry to further enhance quality and interpretability of the implemented AI/ ML paradigms [26–31]. Finally, the deployment, transparency and ethics (Codes G, H, K, Q, S, T and X) align with regulatory frameworks for POC applications and Software as a Medical Device regulations by the European Economic Area Regulation of the European Union 2017/746 on in vitro diagnostic medical devices [19] and the Food and Drug Administration of the United States [20]. These regulatory bodies mandate quantification of uncertainty (code H), explainability of the implementation (code T) and privacy guidelines (codes Q and X) for clinical approval [26,32–40]. A detailed description of each code with phrasing, definition, criteria for positive or negative occurrence and corresponding exemplars is provided in Table S1 following the guidelines in [41,42].

Notably, the scheme A–Z captures only presence or absence and not the magnitude, for example nanomolar and picomolar both get binned as 'Low LOD'. The exact values are described in corresponding tabulated data in the main manuscript within their respective sections. Additionally, codes such as temporal drift do not register the exact rate of the drift, which is essential for recalibration in wearable devices [43]. The coding scheme explicitly evaluates outcomes (overfitting, interpretability) without specific bias estimates due to high heterogeneity in implementations (e.g. spatial features using CNN [44] as opposed to drift tracking using LSTMs [45]). As the field of electrochemistry advances in conjunction with AL/ ML paradigms, the themes of green chemistry, energy efficiency of paradigms, cost-estimates per sensor and test would also become more relevant in future systematic examinations using such network mapping approaches.

S1.2. Systematic Evidence Maps' Methodology

The Excel file with each row summarizing the examined studies was imported into Python using the Pandas read excel method. Before processing further, logical consistencies were checked and the code columns of advantages, disadvantages, strengths and weaknesses were expanded using the comma symbol. Finer subdivisions were then created by using a dictionary data structure for mapping a code as advantage or disadvantage string depending on the occurrence of code within respective columns. The entire string representing a study's characteristics was then treated as a descriptive path. Subsequently, each unique string originating from the data frame column was treated as the nodes contributing to the networks. The final networks were then captured using Selenium and ChromeDriver for hierarchical repulsion network layout. The legend was created using HTML elements and composited with the final network using GIMP 3.0.

S1.2.1. Joint-probability Network

This network provides an undirected and bidirectional relationship summary of the coding scheme, representing % within a column. The frequency of nodes was iteratively updated and each consecutive edge was retained. Each unique edge is counted only once ensuring exact prevalence in reported percentages. Additionally, the undirected nature implies that the network is readable in both directions (path A to B and B to A are symmetrical). The ordering of columns (and thus edge observance) is theoretically guided, for example, model input takes precedence over output, thus the column order influences observance of edges. The node percentages were calculated within each column, and the network was filtered at 5% to reduce node cluttering. The joint-probability was calculated as the ratio of edge frequency and to the total references observed (length of data frame) for a given edge. Edge cluttering was reduced by filtering edges for probability > 5%. After final filtering, 65 out of 658 unique edges remained. All edges were colored based on nodes, and nodes were colored based on the column of origin. The network provides a general overview (percentage summaries) without accounting for true prevalence. Implementation details are as follows:

Column-wise node frequency:

$$Node\ \% \ = \left(\frac{Node\ count\ in\ a\ given\ column}{Total\ nodes\ in\ the\ given\ column}\right) \times 100$$

Node pair as edge within a path: $O(NxCxM)$ complexity for N rows, C columns and M nodes per column.

Edge frequency across all paths:

$$Edge\ \% \ = \left(\frac{Studies\ containing\ both\ nodes}{Total\ number\ of\ studies}\right) \times 100$$

S1.2.2. Contiguous Doublets and Triplets in Paths Approach

This network represents directed and ordered relationships with all significant pathways highlighted according to pre-defined probability thresholds. For the contiguous paths network, all 2-node sub-paths (doublets) and 3-node sub-paths (triplets) within and across columns were computed (true co-occurrence frequency). The entire network's nodes and edges were filtered at 10% probability. Calculated node percentages were added to the node label and node sizing was set according to prevalence within the column of origin and colored accordingly. The network was filtered at >10% probability. To highlight critical paths (edges participating in high-probability triplets) the following logic was adopted: (a) Baseline edge width was proportional to sub-path probability and a green color was assigned, however for probability >0.5, the edge was colored red indicating strong triplet participation for code set, (b) If probability >0.5, a dark green color indicates strong doublets. This network represents true prevalence across studies without causal links/ inference, and only sequential relationships are investigated quantitatively. After final filtering 66 doublet sub-paths out of total 617 and 50 triplet sub-paths out of total 1036 triplets were visualized in the network. Due to visualization limitation (representation of triplet probabilities in network paths), the computation for triplets in all 50 sub-paths is provided in S1.3. Implementation details are as follows:

Column-wise node frequency:

$$Node\ \% = \left(\frac{Node\ count\ in\ a\ given\ column}{Total\ nodes\ in\ the\ given\ column}\right) \times 100$$

Computing consecutive node triplets $(\mathrm{i}, \mathrm{i}+1, \mathrm{i}+2)$: $O(NxCxM^2)$ complexity for N rows, C columns and M^2 nodes per column.

Updating edge frequency across all paths: The recurring and ordered sub-paths include doublets (two consecutive nodes) and triplets (three consecutive nodes). These represent the shared ordered patterns within the dataset: the precedence of frequently occurring characteristics, two and three node patterns, and participation of doublets within high-probability triplets. All assessment codes were seperated in individual categories: 2 advantages and disadvantages groups for Electrochemical Characteristics, and 6 strengths and weaknesses groups for Model Performance and Robustness, Data Infrastructure and Quality, and Deployment, Transparency and Ethics groups.

Each doublet was counted as follows:

$$P(A \to B) = \left(\frac{Number\ of\ studies\ containing\ A \to B}{Total\ number\ of\ studies}\right)$$

Where, if $P(A \to B) \geq\ 0.5 \}$ node color = dark green ; these edges indicate common directed doublets.

Each triplet was counted as:

$$P(A \to B \to C) = \left(\frac{Number\ of\ studies\ containing\ A \to B \to C}{Total\ number\ of\ studies}\right)$$

Where, if $P(A \to B \to C) \geq\ 0.5 \}$ node color = red; these indicate common triplets.

All observed paths reflect the theoretical order from the final curated dataset (presented in dotplots in the main manuscript), where changing the inclusion criteria or coding rules also modify the underlying network paths.

S1.3. Supplementary Results for Contiguous Doublets and Triplets Analysis

This section provides the details of probability computations for triplets not visible in in the main manuscript Figure 10. All observed triplets with at-least 10% probability (n=50) are presented in descending order as follows:

1. High data signal quality → High-quality dataset requirement → Low training resource requirements: 56.4% (75/133)
2. Low training resource requirements → Low data scalability → On request access for dataset: 55.6% (74/133)
3. High-quality dataset requirement → Low training resource requirements → Low data scalability: 52.6% (70/133)
4. Automated processing → No issues with privacy → Real-time performance: 47.4% (63/133)
5. Low maintenance/calibration needs → Automated processing → No issues with privacy: 44.4% (59/133)
6. High sensitivity and precision → Enhances multiplexing → No risk of overfitting: 42.9% (57/133)
7. Vulnerable to adversarial perturbations → High data signal quality → High-quality dataset requirement: 39.8% (53/133)
8. Selective electrode → High reproducibility of biosensor/sensor → Low deposition time: 37.6% (50/133)
9. Wide LDR → Selective electrode → High reproducibility of biosensor/sensor: 37.6% (50/133)
10. On request access for dataset → Low maintenance/calibration needs → Automated processing: 36.8% (49/133)
11. Enhances multiplexing → No risk of overfitting → Generalization of AI/ML approach: 36.1% (48/133)
12. Low LOD → Wide LDR → Selective electrode: 34.6% (46/133)
13. Stable temporal response → Environmentally robust → Robust to complex sample matrices: 32.3% (43/133)
14. Low data scalability → On request access for dataset → Low maintenance/calibration needs: 31.6% (42/133)
15. High reproducibility of biosensor/sensor → Low deposition time → –: 27.1% (36/133)
16. Robust to complex sample matrices → Widely applicable → High sensitivity and precision: 27.1% (36/133)
17. High sensitivity and precision → No risk of overfitting → Generalization of AI/ML approach: 25.6% (34/133)
18. Widely applicable → High sensitivity and precision → Enhances multiplexing: 25.6% (34/133)
19. Concentration Output → Stable temporal response → Environmentally robust: 24.1% (32/133)
20. No issues with privacy → Real-time performance → Interpretable and transparent: 23.3% (31/133)
21. Environmentally robust → Robust to complex sample matrices → High sensitivity and precision: 22.6% (30/133)
22. Voltammetric Features → Concentration Output → Stable temporal response: 22.6% (30/133)
23. Robust to complex sample matrices → High sensitivity and precision → No risk of overfitting: 19.5% (26/133)
24. Robust to complex sample matrices → High sensitivity and precision → Enhances multiplexing: 18.8% (25/133)
25. Stable temporal response → Robust to complex sample matrices → High sensitivity and precision: 18.0% (24/133)
26. Environmentally robust → Robust to complex sample matrices → Widely applicable: 17.3% (23/133)
27. Real-time performance → Interpretable and transparent → Suitable for edge computation: 16.5% (22/133)
28. Delayed performance → Issue with model interpretability and transparency → Not suitable for edge computation: 15.8% (21/133)

29. Issues with cross-reactivity → Vulnerable to adversarial perturbations → High data signal quality: 15.8% (21/133)
30. Selective electrode → High reproducibility of biosensor/sensor → Long deposition time: 15.8% (21/133)
31. Environmental sensitivity mismatch → Sample matrix effects degrade performance → Limited applicability: 15.0% (20/133)
32. Low deposition time → '– '(All Electrochemical Characteristics Advantages) → Voltammetric Features: 15.0% (20/133)
33. DPV → Low LOD → Wide LDR: 14.3% (19/133)
34. High-quality dataset requirement → Low training resource requirements → On request access for dataset: 14.3% (19/133)
35. Limited applicability → Issues with cross-reactivity → Vulnerable to adversarial perturbations: 14.3% (19/133)
36. No issues with privacy → Real-time performance → Suitable for edge computation: 14.3% (19/133)
37. No risk of overfitting → Generalization of AI/ML approach → Environmental sensitivity mismatch: 14.3% (19/133)
38. Quantified prediction uncertainty → Automated processing → No issues with privacy: 14.3% (19/133)
39. Concentration Output → Stable temporal response → Robust to complex sample matrices: 13.5% (18/133)
40. No risk of overfitting → Generalization of AI/ML approach → Temporal drift & signal decay: 13.5% (18/133)
41. No uncertainty quantification in predictions → Delayed performance → Issue with model interpretability and transparency: 12.8% (17/133)
42. Real-time performance → Suitable for edge computation → No uncertainty quantification in predictions: 12.8% (17/133)
43. Suitable for edge computation → No uncertainty quantification in predictions → Issue with model interpretability and transparency: 12.8% (17/133)
44. No risk of overfitting → Generalization of AI/ML approach → Limited applicability: 12.0% (16/133)
45. Low maintenance/calibration needs → Quantified prediction uncertainty → Automated processing: 11.3% (15/133)
46. Generalization of AI/ML approach → Environmental sensitivity mismatch → Limited applicability: 10.5% (14/133)
47. High data scalability → High data signal quality → High-quality dataset requirement: 10.5% (14/133)
48. Issue with AI/ML approach generalization → Vulnerable to adversarial perturbations → High data signal quality: 10.5% (14/133)
49. Low data scalability → On request access for dataset → Automated processing: 10.5% (14/133)
50. Temporal drift & signal decay → Environmental sensitivity mismatch → Sample matrix effects degrade performance: 10.5% (14/133)

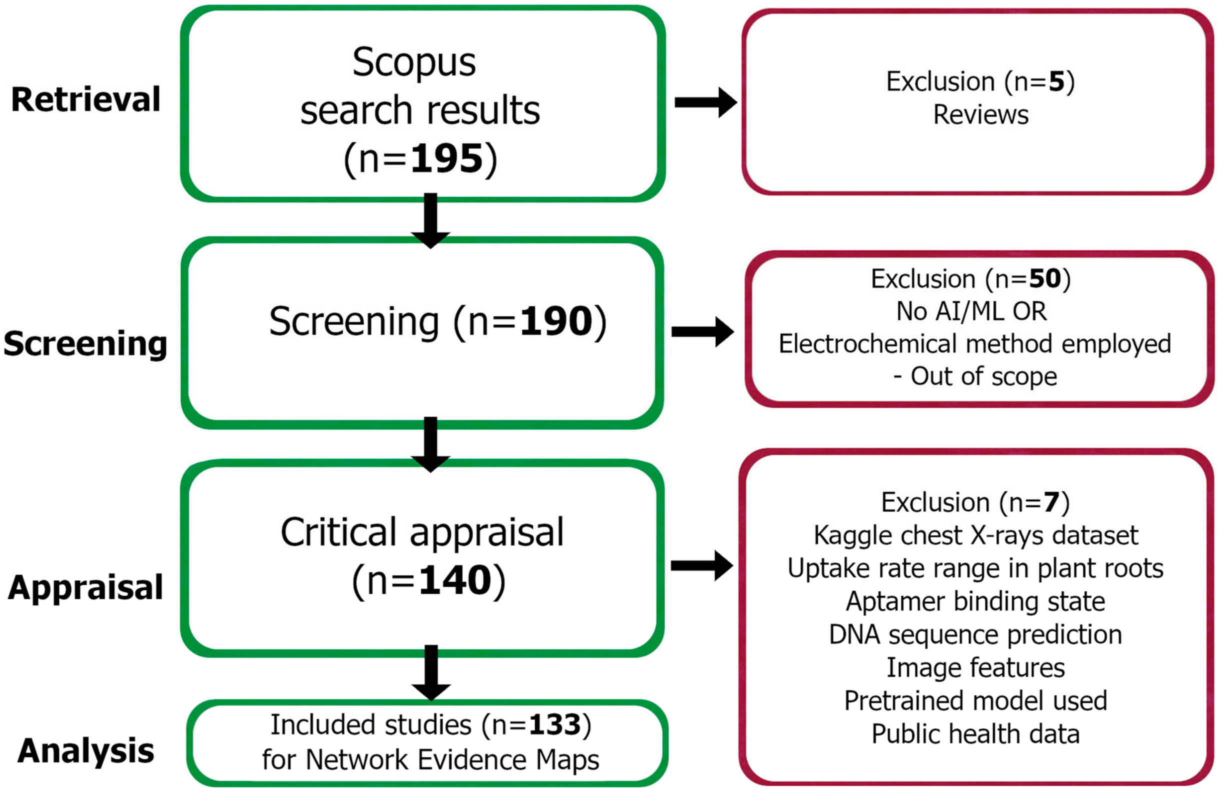

Figure S1. Flow diagram of study counts during retrieval, screening, appraisal and analysis stages of the review.

Table S1: Coding manual for positive and negative coded characteristics employed in the review

Cod e	Scope	Positive Characteristic (Advantages/ Strengths)	Negative Characteristic (Disadvantages/ Weaknesses)
		**Electrochemical Biosensor/ Sensor Characteristics** *These codes relate to the (bio)sensor platform itself.*	
**A**	Limit of Detection: Defined by IUPAC and clinical chemistry standards [2]	Low Limit of Detection<br>Criteria: Study demonstrates that the ML model recovers signals at physical detection limits/ Low LOD (bio)sensor is present<br>Example: AI/ML denoising promotes low LOD [46,47]	High Limit of Detection<br>Criteria: Study demonstrates that the ML model recovers signals at physical detection limits/ High LOD (bio)sensor is present<br>Example: Poor signal-to-noise ratio forces models near boundary conditions [48,49]
**B**	Linear Dynamic Range	Wide Linear Dynamic Range<br>Criteria: ML model successfully expands the concentration range/ Wide LDR is apparent for the fabricated (bio)sensor<br>Examples: ML regression enables linearization of non-linear sensor responses [47]	Narrow Linear Dynamic Range<br>Criteria: ML fails to expand the concentration range/ Narrow LDR is apparent for the fabricated (bio)sensor<br>Examples: Models fail outside training ranges [50] or require additional sample dilution [49]
**C**	Selectivity: AI/ ML paradigms can aid in resolving overlapping peaks [3]	Selective: Reduces the ML's burden to resolve peaks<br>Criteria: Sensor natively distinguishes the target analyte, reducing the computational complexity<br>Examples: Minimal interference (<5%) via coordination [51], MIP cavities tuned to Zika Virus particles [52]	Non-selective: AI/ ML paradigm compensates for non-selectivity<br>Criteria: Sole reliance on AI/ ML paradigm for the target analyte, failing for novel interferents<br>Examples: Electrochemical tongue-assisted via pattern recognition [53], insufficient selectivity of the fabricated sensor for respective analytes [54]
**D**	Reproducibility: relevant to domain gaps in AI/ ML paradigms [4]	High reproducibility of (bio)sensor: Enables transfer learning across batches<br>Criteria: High-performance despite batch-to-batch variation/ reports of standard deviation/ error metrices<br>Examples: RSD<0.36% across 50 days stability [55], high reproducibility with low RSD (n=10) [56]	Low reproducibility of (bio)sensor: Models overfit to artifacts/ noise as a consequence of low reproducibility<br>Criteria: Low-performance in lieu of batch-to-batch variation/ no standard deviation/ error metrices reported<br>Examples: Reporting of intra-batch variation requiring ML compensation [57,58], absence of inter-batch reproducibility metrices [59]
**E**	Deposition Time: AI can accelerate discovery and optimization of materials [5]	Low deposition time: Enables rapid sensor fabrication<br>Criteria: Fast fabrication process or such process directly facilitates the generation of sufficient training data<br>Examples: Rapid electropolymerization [60], under-minute detection [61]	Long deposition time: Limits dataset size and rapid deployment<br>Criteria: Slow fabrication process or such process limiting data collection or size<br>Examples: Additional curing or drying time [52,62], resting time for optimal analyte adsorption [51]
**–**	Empty character (en-dash symbol)	If no advantages are present assign '–'	If no disadvantages are present assign '–'

**Model Performance and Robustness**<br>*These codes relate to the AI/ML paradigm implementation and core performance metrics.*			
**F**	Temporal Stability: AI/ML enables drift compensation [8,9]	Stable temporal response: Extends the validity of model up to weeks/ months<br>Criteria: Model maintains accuracy over extended periods without frequent recalibration<br>Examples: 20-day stability and 81–87% signal retention [63,64]	Temporal drift: Fouling effects result in signal peak shifting<br>Criteria: Model's performance degrades over time due to sensor fouling or signal decay/ no discussion of adaptation to such drift effects<br>Examples: Fluctuations in temperature, degradation over measurement cycles [65,66]
**I**	Environmental Robustness: Physical constraints embedded into AI./ML architectures empower lab-to-field translation [10]	Environmentally robust: ML aids in adjustment of readings according to the environmental parameters<br>Criteria: Model explicitly accounts/ compensates for environmental variable<br>Examples: Multiple pH, temperature and ambient conditions [46,63], sensor validated in artificial and leach-out tests across 2–11 pH [64]	Environmental sensitivity mismatch: Lab-trained models fail in field applications<br>Criteria: Model was trained under controlled lab conditions<br>Examples: Sensor performs optimally at strict pH or narrow pH range only [48,51,58]
**J**	Sample Matrix Effects: Domain adaptation for lot-to-lot and site-to-site shift [11]	Robust to complex sample matrices: Domain adaptation for features<br>Criteria: Model successfully predicts concentrations in complex real-world samples compared to buffer<br>Examples: High recoveries in real samples [48,67]	Sample matrix effects degrade performance: Issues with mass transport or potential electrode fouling effects<br>Criteria: Model's accuracy drops significantly when tested in real biological or environmental matrices compared to buffer<br>Examples: Lack of real sample validation, exclusively validated in buffers [54,68,69]
**L**	Applicability Scope: AI/ ML aid in evolving situations for calibration transfer and maintenance [12]	Widely applicable: Transfer learning enables adaptation to multiple analytes<br>Criteria: AI/ML approach can be easily adapted to detect multiple different analytes and/or across different sensing platforms<br>Examples: Food safety, gas pollution and human health [67], multiple antibiotics and drugs [70,71]	Limited applicability: Models are limited to single analytes<br>Criteria: Model is highly specialized for a single analyte and/ or cannot be adapted or transferred to other targets<br>Examples: Exclusive to single biomolecule, pesticide or virus detection [47,51,52]
**M**	Sensitivity and Precision: AI/ML's potential for electrochemistry enabling informed decision-making [13,14]	High sensitivity and precision: AI/ML paradigm estimate subtle signal features<br>Criteria: Model demonstrably improves the LOD, SNR, or predictive precision compared to traditional analytical methods<br>Examples: exceptional performance as per AI./ML performance metrices [46,60]	Low sensitivity and precision: Amplified noise or model learning spurious correlations<br>Criteria: Model exhibits high prediction variance, fails at low concentrations, or amplifies background noise<br>Examples: Raw signals of poor quality, described as chaotic and overlapping [59,65]
**R**	Multiplexity: AI/ML paradigms	Enhances Multiplexing: ML deconvolutes overlapping signals and	Issues with cross-reactivity: Interferents result in non-linear mixing of signals

	can aid in resolving signal peaks in multiplexed sensing [17]	enables multi-analyte detection<br>Criteria: Model successfully enables the simultaneous detection of multiple analytes from a single sensor or scan/ resolving overlapping peaks<br>Examples: Simultaneous multiple analytes detected [48,72] or multiple electrodes resolve cross-reactive analytes [67]	Criteria: Model fails to distinguish between the target analyte and interferents, resulting in false positives or skewed concentrations<br>Examples: Peak current reduction when Cd/Pb coexist with Ni/Cu [58], explicit interference requiring ML correction [73]
**V**	Risk of Overfitting: Advances in AI/ML research tackling overfitting can aid in electrochemical sensing [15,16,32]	No risk of overfitting: Regularization and proper cross-validation<br>Criteria: Model performs well on unseen test data/ plausible AI/ ML performance metrices<br>Examples: Cross-validation with separate reporting of validation error [51], k-fold cross-validation performed [55,60]	Risk of overfitting: Model retains sensor-specific artifacts during testing<br>Criteria: Model fails on unseen test data/ implausible AI/ ML performance metrices<br>Examples: Limited discussion of regularization [74], sparse datasets used for model training [59,75]
**W**	Generalization: Barrier to POC-testing applications, diverse cohort improves generalization [14,19,20]	Generalization of AI/ ML approach: Models perform well across environmental conditions/ use cases<br>Criteria: Model is validated across diverse conditions/ not requiring user-specific recalibration.<br>Example: Generalization from buffer to complex matrices [46,63]	Issue with AI/ ML approach generalization: Model fails outside lab conditions<br>Criteria: Model is validated across narrow conditions/ requiring user-specific recalibration.<br>Example: Not transferable to other sample types or sites [52,76], or measurements performed under controlled lab conditions [53]
**Z**	Adversarial Robustness: Adversarial training enables successful IoT and sensor network integration [18]	Resilient to input perturbations: Adversarial training prepares for worst-case noise<br>Criteria: Study demonstrates that the model maintains accurate predictions even when the input signal is subjected to intentional noise<br>Examples: Model performs well despite introduction of noise [77–79]	Vulnerable to adversarial perturbations: Addition of small noise alters model performance<br>Criteria: Study omits adversarial procedures/ no report of such a procedure<br>Examples: No explicit testing against input perturbations/ manipulations [48,51,52]
		**Data Infrastructure and Quality**<br>***These codes relate to the data and computational requirements.***	
**N**	Data Scalability: Advancements addressing scalability bottlenecks allow efficient healthcare IoT applications [25]	High data scalability: Large datasets are beneficial for generalizability, cloud-based paradigms aggregates data<br>Criteria: Data pipeline or architecture allows for seamless scaling to accommodate larger datasets without performance degradation<br>Examples: Large datasets/ datapoints via simulation [68,74]	Low data scalability: Sparse datasets plateau model performance<br>Criteria: Model architecture is compromised by data size/ computational approach cannot scale to handle larger datasets<br>Examples: Limited datasets used (19–60 samples) [47,69,80]
**O**	Data Accessibility: FAIR principles	Data availability: Public datasets enable benchmarking	On request access for dataset: Hinders transparency of implementation and

	enable validation and benchmarking [24]	Criteria: Raw data, code, or trained models are made publicly available<br>Examples: Data available via GitHub, or supplementary [77,81], shared large datasets [70,82]	independent validation<br>Criteria: Access is restricted or only provided upon direct request to the authors<br>Examples: Data made available upon (reasonable) request [51,52]
**P**	Signal Quality: Tradeoff between noise and signal preservation is well documented [30,31]	High data signal quality: Ensures clean input and requirement of additional denoising steps<br>Criteria: Raw electrochemical signals are of high-quality decreasing burden on AI/ML approach<br>Examples: Well-defined voltammetric peaks [61,63]	Low data signal quality: ML compensation for denoising<br>Criteria: Raw electrochemical signals are of noisy/ require denoising algorithms<br>Examples: Raw signals require noise/ drift corrections [53,83]
**U**	Dataset Quality Requirement: ML models propagate systematic biases due to noisy labels [28,29]	High-quality dataset requirement: Well annotated/ labeled data<br>Criteria: Study emphasizes the use of high-quality, validated, reference-method-labelled data<br>Examples: Real samples with clinical characterization, ground-truth with validation of samples [51,84]	No high-quality dataset requirement: Model training was performed on noisy labels<br>Criteria: Model prone to systematic biases/ low-quality data/ absence of ground-truth<br>Examples: Heavy reliance on simulated data [54]
**Y**	Training Resource Requirements: Lightweight architectures and scaling techniques empower edge-devices [26,27]	Low training resource requirements: Enables rapid deployment on resource-constrained devices<br>Criteria: Model can be trained efficiently on standard hardware/ low training time<br>Examples: No requirement for specialized GPU [61,69]	High computational training cost / long training time: GPU-intensive architectures prevent deployment or application in resource-constrained settings<br>Criteria: Model requires specialized hardware/ long training time<br>Examples: High-end systems utilized for model training and prediction [65,82]
	**Deployment, Transparency and Ethics**<br>***These codes relate to the operational usability, interpretability, and maintenance overhead of the AI/ML implementation and ethical (privacy and security concerns) in case of biomedical applications.***		
**G**	Calibration Burden: Self-calibrating or drift-resistant ML models empower set-and-forget paradigms [35]	Low maintenance/calibration needs: Drift-resistant paradigms are implemented with minimal recalibration<br>Criteria: Deployed system requires minimal to no recalibration<br>Examples: Smartphone-integrated and set-and-forget implementations [64,84,85]	High calibration burden and maintenance overhead: Frequent recalibration defeats the purpose of AI/ ML implementation<br>Criteria: Deployed system requires frequent/ manual recalibration<br>Examples: Multiple calibration steps or standard addition method [49,51]
**H***	Uncertainty Quantification (UQ): Confidence intervals prevent overconfidence near diagnostic cut-offs [15,32]	Quantified prediction uncertainty: Bayesian methods output prediction intervals<br>Criteria: Model provides a measure of confidence, error bars, or prediction intervals in addition to point estimates<br>Examples: Models report uncertainty or utilize confidence intervals [77,85]	No uncertainty quantification in predictions: Point estimates fail near critical thresholds resulting in false classification<br>Criteria: Model outputs only deterministic point estimates without confidence/ variance/ error estimates<br>Example: Binary classification without

			confidence intervals [48,52]
**K**	Processing Automation: End-to-end streamlining reduces potential for human-error [36]	Automated processing: Streamlined setup minimizes the potential for human error<br>Criteria: Data processing pipeline is fully automated from raw signal input to final prediction<br>Examples: End-to-end implementations and automated extractions [48,60,67]	Manual processing: Increases human bias and negatively affect scalability<br>Criteria: Workflow requires manual baseline correction, peak selection, or human curation<br>Examples: Human screening prior to ML processing [86], manual cleaning of samples [87]
**Q**	Privacy: HIPAA/GDPR compliance through blockchain and decentralization for tackling privacy and security concerns [37,38]	No issues with privacy: On-device inference ensures local processing (clinical samples), or environmental applications<br>Criteria: Data processing and inference occur entirely on the edge/local device, with no sensitive raw data (clinical information) transmitted to external servers<br>Examples: Environmental/ pharmaceutical application imply no personal health data [60,63]	Issues with privacy: Cloud-dependent or third-party involvement compromises privacy and security<br>Criteria: System requires cloud computing and requires the transmission of raw, unencrypted, or identifiable health data over a network<br>Examples: Web apps for regulated biomolecules [55], data transmissions involving remote server [88]
**S**	Real-Time Performance: Inference times are compatible with physiological feedback loops for real time monitoring [39,40]	Real-time performance: Lightweight implementations enable closed-loop control and aid in rapid analyte detection<br>Criteria: Model can process inputs and generate predictions fast enough to support real-time, closed-loop, or immediate decision-making<br>Examples: Quantized model with 33ms enables real-time performance [77], ESP32 based device without batch performance delays [67]	Delayed performance: Batch processing breaks feedback loops, latencies caused due to external processing of datasets<br>Criteria: Model's inference time is too slow for real-time applications/ requires batch processing or introducing significant latency<br>Examples: no explicit discussion of interference latency [51], offline modelling in MATLAB [89]
**T**	Interpretability: Post-hoc interpretability is essential for debugging model failures points [33]	Interpretable and transparent: Paradigms strengthened via SHAP/ LIME/ PLS enable analyses of feature importance<br>Criteria: Study employs explainable AI (XAI) techniques/ uses inherently interpretable models, to clarify how inputs influence the final prediction<br>Examples: SHAP for determining feature importance [60,84]	Issue with model interpretability and transparency: Interpretable models, where feature importance or prediction rules are not established<br>Criteria: Model operates as a "black box" lacking specifics of decision-making process<br>Examples: AI/ ML paradigms deployed without interpretability tools [48,52,90]
**X**	Edge Computability: Enabling edge-inference on resource-constrained devices [26,39,40]	Suitable for edge computation: Quantized models deployed on microcontrollers enable on-site applications<br>Criteria: Model is optimized (e.g., quantized, pruned) to run efficiently on resource-constrained edge devices or microcontrollers without internet connectivity	Not suitable for edge computation: Cloud dependency introduces additional latency (apart from privacy concerns)<br>Criteria: Model requires cloud infrastructure/ not suitable for edge devices/ requires high-performance computing<br>Examples: Streamlit deployment and requirement for internet connectivity [92],

	Examples: ESP32 with Tiny ML [77], SVM with linear kernel on ATmega328 device [91]	implementation on Tesla T4 GPU workstation [93]

[*]**Operational Definition**: UQ is coded as 'present' only if the study reports per-prediction confidence intervals, prediction intervals, Bayesian credible intervals, conformal prediction bands, or explicit variance/error estimates for individual predictions. Aggregate performance metrics (R^2, RMSE, MAE) alone do not satisfy this criterion.
Abbreviations: AI –Artificial Intelligence; GDPR –General Data Protection Regulation; HIPAA –Health Insurance Portability and Accountability Act; IoT –Internet of Things; IUPAC –International Union of Pure and Applied Chemistry; LDR –Linear Dynamic Range; LOD –Limit of Detection; ML –Machine Learning; POC –Point-of-Care

References

1. Krippendorff, K. Recording/Coding. In *Content Analysis: An Introduction to Its Methodology*; SAGE Publications, Inc., 2019; pp. 128–156 ISBN 978-1-0718-7878-1.
2. Armbruster, D.A.; Pry, T. Limit of Blank, Limit of Detection and Limit of Quantitation. *Clin Biochem Rev* **2008**, *29 Suppl 1*, S49-52.
3. Liu, J.; Li, J.; Xu, Y.; Low, S.S.; Ning, H.; Yu, S.; Xu, D.; Liu, Q. A Fused Convolutional Transformer for Voltammetric Electronic Tongue Analysis Tasks. *Journal of Environmental Chemical Engineering* **2024**, *12*, 113462, doi:10.1016/j.jece.2024.113462.
4. Shi, Y.; Ying, X.; Yang, J. Deep Unsupervised Domain Adaptation with Time Series Sensor Data: A Survey. *Sensors (Basel)* **2022**, *22*, 5507, doi:10.3390/s22155507.
5. Harris, S.B.; Biswas, A.; Yun, S.J.; Roccapriore, K.M.; Rouleau, C.M.; Puretzky, A.A.; Vasudevan, R.K.; Geohegan, D.B.; Xiao, K. Autonomous Synthesis of Thin Film Materials with Pulsed Laser Deposition Enabled by In Situ Spectroscopy and Automation. *Small Methods* **2024**, *8*, e2301763, doi:10.1002/smtd.202301763.
6. Saqib, M.; Solomonenko, A.N.; Hazra, N.K.; Aljasar, S.A.; Korotkova, E.I.; Dorozhko, E.V.; Vashisth, M.; Kar, P.K. Electrochemical Detection of Heavy Metals Using Graphene-Based Sensors: Advances, Meta-Analysis, Toxicity, and Sustainable Development Challenges. *Biosensors* **2025**, *15*, 505, doi:10.3390/bios15080505.
7. Saqib, M.; Vashisth, M.; Korotkova, E.I.; Hui, A.L.; Aremu, S.O.; Das, S.; Deb, A.; Hazra, N.K.; Saha, R.; Saha, S.; et al. Recent Advances in Electrochemical Detection of Antibiotics on Graphene-Based Sensors and Biosensors, Impact and Sustainable Development Challenges: A Systematic Review and Meta-Analysis. *Biosensors* **2026**, doi:10.3390/bios16050234.
8. Vergara, A.; Vembu, S.; Ayhan, T.; Ryan, M.A.; Homer, M.L.; Huerta, R. Chemical Gas Sensor Drift Compensation Using Classifier Ensembles. *Sensors and Actuators B: Chemical* **2012**, *166–167*, 320–329, doi:10.1016/j.snb.2012.01.074.
9. Krayden, A.; Avraham, M.; Ashkar, H.; Blank, T.; Stolyarova, S.; Nemirovsky, Y. TinyML-Based Real-Time Drift Compensation for Gas Sensors Using Spectral–Temporal Neural Networks. *Chemosensors* **2025**, *13*, 223, doi:10.3390/chemosensors13070223.
10. Raissi, M.; Perdikaris, P.; Karniadakis, G.E. Physics-Informed Neural Networks: A Deep Learning Framework for Solving Forward and Inverse Problems Involving Nonlinear Partial Differential Equations. *Journal of Computational Physics* **2019**, *378*, 686–707, doi:10.1016/j.jcp.2018.10.045.
11. Deshmukh, M.T.; Thakre, Y.; Bisen, W.H.; Sankar, K.; Avthankar, A.; Shahade, A.K.; Bhaiyya, M.; Kulkarni, M.B. From Drops to Decisions: AI/ML-Driven Biofluidics for Clinical Diagnostics and Healthcare Intelligence. *Anal Chem 98*, 24–42, doi:10.1021/acs.analchem.5c06356.
12. Nikzad-Langerodi, R.; Andries, E. A Chemometrician's Guide to Transfer Learning. *Journal of Chemometrics* **2021**, *35*, e3379, doi:10.1002/cem.3379.
13. LeCun, Y.; Bengio, Y.; Hinton, G. Deep Learning. *Nature* **2015**, *521*, 436–444, doi:10.1038/nature14539.
14. Shi, X.; Zhang, G.; Lu, Y.; Pang, H. Applications of Machine Learning in Electrochemistry. *Renewables* **2023**, *1*, 1–26, doi:10.31635/rnwb.023.202300039.
15. Srivastava, N.; Hinton, G.; Krizhevsky, A.; Sutskever, I.; Salakhutdinov, R. Dropout: A Simple Way to Prevent Neural Networks from Overfitting. *J. Mach. Learn. Res.* **2014**, *15*, 1929–1958.

16. Kang, M.; Kim, D.; Kim, J.; Kim, N.; Lee, S. Strategies to Enrich Electrochemical Sensing Data with Analytical Relevance for Machine Learning Applications: A Focused Review. *Sensors (Basel)* **2024**, *24*, 3855, doi:10.3390/s24123855.
17. Antunes, M.C.; J. Simão, J.E.; Duarte, A.C.; Tauler, R. Multivariate Curve Resolution of Overlapping Voltammetric Peaks: Quantitative Analysis of Binary and Quaternary Metal Mixtures. *Analyst* **2002**, *127*, 809–817, doi:10.1039/b200243b.
18. Madry, A.; Makelov, A.; Schmidt, L.; Tsipras, D.; Vladu, A. Towards Deep Learning Models Resistant to Adversarial Attacks.; February 15 2018.
19. Kahles, A.; Goldschmid, H.; Volckmar, A.-L.; Ploeger, C.; Kazdal, D.; Penzel, R.; Budczies, J.; Flechtenmacher, C.; Gassner, U.M.; Brüggemann, M.; et al. Regulation (EU) 2017/746 (IVDR): Practical Implementation of Annex I in Pathology. *Pathologie (Heidelb)* **2023**, *44*, 86–95, doi:10.1007/s00292-023-01274-6.
20. Commissioner, O. of the FDA Releases Artificial Intelligence/Machine Learning Action Plan Available online: https://www.fda.gov/news-events/press-announcements/fda-releases-artificial-intelligencemachine-learning-action-plan (accessed on 1 August 2026).
21. Sánchez Sánchez, P.M.; Huertas Celdrán, A.; Bovet, G.; Martínez Pérez, G. Adversarial Attacks and Defenses on ML- and Hardware-Based IoT Device Fingerprinting and Identification. *Future Generation Computer Systems* **2024**, *152*, 30–42, doi:10.1016/j.future.2023.10.011.
22. Richiardi, J.; Ravano, V.; Molchanova, N.; Gordaliza, P.M.; Kober, T.; Cuadra, M.B. Chapter 6 - Domain Shift, Domain Adaptation, and Generalization: A Focus on MRI. In *Trustworthy AI in Medical Imaging*; Lorenzi, M., Zuluaga, M.A., Eds.; The MICCAI Society book Series; Academic Press, 2025; pp. 127–151 ISBN 978-0-443-23761-4.
23. Liu, C.; Deng, Z.; Zhang, X.; Bao, H.; Cheng, D. Battery State of Health Estimation across Electrochemistry and Working Conditions Based on Domain Adaptation. *Energy* **2024**, *297*, 131294, doi:10.1016/j.energy.2024.131294.
24. Wilkinson, M.D.; Dumontier, M.; Aalbersberg, I.J.J.; Appleton, G.; Axton, M.; Baak, A.; Blomberg, N.; Boiten, J.-W.; da Silva Santos, L.B.; Bourne, P.E.; et al. The FAIR Guiding Principles for Scientific Data Management and Stewardship. *Sci Data* **2016**, *3*, 160018, doi:10.1038/sdata.2016.18.
25. Rieke, N.; Hancox, J.; Li, W.; Milletarì, F.; Roth, H.R.; Albarqouni, S.; Bakas, S.; Galtier, M.N.; Landman, B.A.; Maier-Hein, K.; et al. The Future of Digital Health with Federated Learning. *npj Digit. Med.* **2020**, *3*, 119, doi:10.1038/s41746-020-00323-1.
26. Baek, Y.; Bae, B.; Shin, H.; Sonnadara, C.; Cho, H.; Lin, C.-Y.; Mu, Y.; Shen, C.; Shah, S.; Wang, G.; et al. Edge Intelligence through In-Sensor and near-Sensor Computing for the Artificial Intelligence of Things. *npj Unconv. Comput.* **2025**, *2*, 25, doi:10.1038/s44335-025-00040-6.
27. Tan, M.; Le, Q.V. EfficientNet: Rethinking Model Scaling for Convolutional Neural Networks 2020.
28. Muller, M.; Wolf, C.T.; Andres, J.; Desmond, M.; Joshi, N.N.; Ashktorab, Z.; Sharma, A.; Brimijoin, K.; Pan, Q.; Duesterwald, E.; et al. Designing Ground Truth and the Social Life of Labels. In Proceedings of the Proceedings of the 2021 CHI Conference on Human Factors in Computing Systems; Association for Computing Machinery: New York, NY, USA, May 7 2021; pp. 1–16.
29. Nou, S.; Lee, J.-S.; Ohyama, N.; Obi, T. The Improvement of Ground Truth Annotation in Public Datasets for Human Detection. *Machine Vision and Applications* **2024**, *35*, 49, doi:10.1007/s00138-024-01527-1.
30. Blitz, J.P.; Klarup, D.G. Signal-to-Noise Ratio, Signal Processing, and Spectral Information in the Instrumental Analysis Laboratory. *J. Chem. Educ.* **2002**, *79*, 1358–1358, doi:10.1021/ed079p1358.
31. Savitzky, A.; Golay, M.J.E. Smoothing and Differentiation of Data by Simplified Least Squares Procedures. *Anal. Chem.* **1964**, *36*, 1627–1639, doi:10.1021/ac60214a047.
32. Gal, Y.; Ghahramani, Z. Dropout as a Bayesian Approximation: Representing Model Uncertainty in Deep Learning. In Proceedings of the Proceedings of the 33rd International Conference on International Conference on Machine Learning - Volume 48; JMLR.org: New York, NY, USA, June 19 2016; pp. 1050–1059.
33. Arrieta, A.B.; Díaz-Rodríguez, N.; Ser, J.D.; Bennetot, A.; Tabik, S.; Barbado, A.; Garcia, S.; Gil-Lopez, S.; Molina, D.; Benjamins, R.; et al. Explainable Artificial Intelligence (XAI): Concepts, Taxonomies, Opportunities and Challenges toward Responsible AI. *Information Fusion* **2020**, *58*, 82–115, doi:https://doi.org/10.1016/j.inffus.2019.12.012.
34. Malin, B.A.; Emam, K.E.; O'Keefe, C.M. Biomedical Data Privacy: Problems, Perspectives, and Recent Advances. *J Am Med Inform Assoc* **2013**, *20*, 2–6, doi:10.1136/amiajnl-2012-001509.

35. Jing, L.; Li, Q.; Li, S.; Li, H.; Xia, F. Toward Autonomous Electrochemical Sensing: Advances and Challenges in Calibration-Free and Long-Duration Technologies. *Acc. Mater. Res.* **2026**, *7*, 721–734, doi:10.1021/accountsmr.6c00038.
36. Rial-Rodríguez, E.; Kappe, C.O.; Laudadio, G. Automating Synthetic Organic Electrochemistry: Concepts and Advancements. *Chemistry–Methods* **2025**, *5*, e202500104, doi:10.1002/cmtd.202500104.
37. Lee, S.; Al-Khalifa, O.; Zhang, S. Communication-Efficient Learning of Deep Networks from Decentralized Data. **2025**, 1310–1321.
38. Barbaria, S.; Jemai, A.; Ceylan, H.İ.; Muntean, R.I.; Dergaa, I.; Boussi Rahmouni, H. Advancing Compliance with HIPAA and GDPR in Healthcare: A Blockchain-Based Strategy for Secure Data Exchange in Clinical Research Involving Private Health Information. *Healthcare (Basel)* **2025**, *13*, 2594, doi:10.3390/healthcare13202594.
39. Prabha, M.; Nandhini, S.; Dayanidhy, M.; Pradeep, R. Edge-AI Integrated Secure Wireless IoT Architecture for Real Time Healthcare Monitoring and Federated Anomaly Detection. *Sci Rep* **2025**, *16*, 574, doi:10.1038/s41598-025-30150-x.
40. Arjunan, G. Optimizing Edge AI for Real-Time Data Processing in IoT Devices: Challenges and Solutions. *International Journal of Scientific Research and Management (IJSRM)* **2023**, *11*, 944–953, doi:10.18535/ijsrm/v11i06.ec2.
41. Nelson, G.; Quinn, S.; Grant, S.; Trevino, S.D.; Day, E.; Schweer-Collins, M.; Carter, H.; Boedeker, P.; Tanner-Smith, E. Ten Practices for Successful Study Coding in Research Syntheses: Developing Coding Manuals and Coding Forms. *Res Synth Methods 16*, 709–728, doi:10.1017/rsm.2025.10019.
42. McKenzie, J.E.; Brennan, S.E.; Ryan, R.E.; Thomson, H.J.; Johnston, R.V. Summarizing Study Characteristics and Preparing for Synthesis. In *Cochrane Handbook for Systematic Reviews of Interventions*; John Wiley & Sons, Ltd, 2019; pp. 229–240 ISBN 978-1-119-53660-4.
43. Takeda, R.; Lisco, G.; Fujisawa, T.; Gastaldi, L.; Tohyama, H.; Tadano, S. Drift Removal for Improving the Accuracy of Gait Parameters Using Wearable Sensor Systems. *Sensors (Basel)* **2014**, *14*, 23230–23247, doi:10.3390/s141223230.
44. Dinesh, G.; Hemakumar, V.S.; Muthevi, A.K.; Kapila, D.; Kumar, N.V.P.S.; Kumar, K.V. Dual-Channel CNN-Enhanced Electrochemical Sensing Using Graphene Platforms for Ultra-Trace Organic Pollutant Detection. *Microchemical Journal* **2025**, *218*, 115330, doi:10.1016/j.microc.2025.115330.
45. Esmaeili, F.; Cassie, E.; Nguyen, H.P.T.; Plank, N.O.V.; Unsworth, C.P.; Wang, A. Predicting Analyte Concentrations from Electrochemical Aptasensor Signals Using LSTM Recurrent Networks. *Bioengineering (Basel)* **2022**, *9*, 529, doi:10.3390/bioengineering9100529.
46. Yousaf, A.; Zhao, Z.; Akhtar, N.; Aizaz, M.; Shahzadi, M. Machine Learning-Optimized Trimetallic MOF Integrated Nanofibers for Precise Detection of p-Nitrophenol in Agricultural Soil. *Chemical Engineering Journal* **2025**, *509*, 161299, doi:10.1016/j.cej.2025.161299.
47. Zhang, H.; Luo, B.; Liu, K.; Wang, C.; Hou, P.; Zhao, C.; Li, A. Development of Surface Molecular-Imprinted Electrochemical Sensor for Palmitic Acid with Machine Learning Assistance. *Talanta* **2024**, *275*, 126124, doi:10.1016/j.talanta.2024.126124.
48. Lahari, S.A.; Kumawat, N.; Amreen, K.; Ponnalagu, R.N.; Goel, S. IoT Integrated and Deep Learning Assisted Electrochemical Sensor for Multiplexed Heavy Metal Sensing in Water Samples. *npj Clean Water* **2025**, *8*, 10, doi:10.1038/s41545-025-00441-x.
49. Kaewket, K.; Outrequin, T.C.R.; Deepaisarn, S.; Wijitsak, J.; Sunon, P.; Ngamchuea, K. Machine Learning-Guided Cobalt@Copper Dual-Metal Electrochemical Sensor for Urinary Creatinine Detection. *ACS Sens.* **2025**, *10*, 3471–3483, doi:10.1021/acssensors.4c03592.
50. Ortega, G.A.; Viltres, H.; Mozaffari, H.; Ahmed, S.R.; Srinivasan, S.; Rajabzadeh, A.R. Ultra-Low Dual Detection of Tetrahydrocannabinol and Cannabidiol in Saliva Based on Electrochemical Sensing and Machine Learning: Overcoming Cross-Interferences and Saliva-to-Saliva Variations. *Sens. Diagn.* **2024**, *3*, 1298–1309, doi:10.1039/D4SD00102H.
51. Khuntia, A.; Kundu, M.; Mahapatra, K.; Kumawat, A. An Electrochemical Detection of Malathion Pesticide Using Cu Electrode and Enhanced by Machine Learning. *Chem Eng & Technol* **2025**, *48*, e70036, doi:10.1002/ceat.70036.
52. Sukjee, W.; Sirisangsawang, P.; Thepparit, C.; Auewarakul, P.; Puttasakul, T.; Sangma, C. MIP-Based Electrochemical Sensor with Machine Learning for Accurate ZIKV Detection in Protein- and Glucose-Rich Urine. *Analytical Biochemistry* **2025**, *702*, 115854, doi:10.1016/j.ab.2025.115854.

53. Arita, R.; Morita, N.; Takemura, K.; Iwasaki, W.; Ueda, S.; Ohmagari, S. Electrochemical Fingerprinting of Complex Solutions Using Boron-Doped Diamond Electrodes: Advanced Classifications by Machine Learning. *Diamond and Related Materials* **2024**, *144*, 110951, doi:10.1016/j.diamond.2024.110951.
54. Du, L.; Rodino, F.; Thoma, Y.; Carrara, S. Identification and Quantification of Multiple Drugs by Machine Learning on Electrochemical Sensors for Therapeutic Drug Monitoring. *IEEE Sens. Lett.* **2024**, *8*, 1–4, doi:10.1109/LSENS.2024.3418197.
55. Pal, A.; Biswas, S.; Chaudhury, K.; Das, S. Paper Sensor Modified with MoS_2 for Detection of Dopamine Using a Machine-Intelligent Web App Interface. *ACS Appl. Mater. Interfaces* **2023**, *15*, 43060–43074, doi:10.1021/acsami.3c03899.
56. Abbas, W.; Zafar, F.; Abou Taleb, M.F.; Ameen, M.; Sami, A.; Mazhar, M.E.; Akhtar, N.; Fazal, M.W.; Ibrahim, M.M.; El-Bahy, Z.M. Machine Learning Trained Poly (3,4-Ethylenedioxythiophene) Functionalized Carbon Matrix Suspended Cu Nanoparticles for Precise Monitoring of Nitrite from Pickled Vegetables. *Food Chemistry* **2024**, *460*, 140395, doi:10.1016/j.foodchem.2024.140395.
57. Kammarchedu, V.; Butler, D.; Ebrahimi, A. A Machine Learning-Based Multimodal Electrochemical Analytical Device Based on eMoSx-LIG for Multiplexed Detection of Tyrosine and Uric Acid in Sweat and Saliva. *Analytica Chimica Acta* **2022**, *1232*, 340447, doi:10.1016/j.aca.2022.340447.
58. Kailasam, V.; Sankararajan, R.; Kailasam, M.; Suseela, S.B. Machine Learning Assisted Metal Oxide-Bismuth Oxy Halide Nanocomposite for Electrochemical Sensing of Heavy Metals in Aqueous Media. *Cryst. Res. Technol.* **2024**, *59*, 2300173, doi:10.1002/crat.202300173.
59. Sun, X.; Liu, F.; Xue, X. Machine Learning Combined with Electrochemical Sensor for Rapid Detection of Sudan Red I in Food. *Food Measure* **2024**, *18*, 95–104, doi:10.1007/s11694-023-02150-w.
60. Dashtaki, R.M.; Dashtaki, S.M.; Heydari-Bafrooei, E.; Piran, M.J. Enhancing the Predictive Performance of Molecularly Imprinted Polymer-Based Electrochemical Sensors Using a Stacking Regressor Ensemble of Machine Learning Models. *ACS Sens.* **2025**, *10*, 3123–3133, doi:10.1021/acssensors.5c00364.
61. Sert, E.; Kalındemirtaş, F.D.; Karakuş, E.E.; Erol, A.; Özbaş, F.; Küçükdeniz, T.; Karakuş, S. Machine Learning Regression for Assessing Sensing Performance and Anticancer Potential of Oolong Tea-Derived Silver Nanoparticles. *J. Electrochem. Soc.* **2025**, *172*, 037503, doi:10.1149/1945-7111/adb90f.
62. Zhao, S.; Liu, W.; Song, D. Rapid Detection and Prediction Model Establishment of Propachlor Residues in Food Assisted by Machine Learning. *Food Measure* **2023**, *17*, 5972–5979, doi:10.1007/s11694-023-02084-3.
63. Oveis Mirabootalebi, S.; Mackie, A.; Vos, G.; Rahimi Azghadi, M.; Liu, Y. Machine Learning-Assisted Pulse Electrodeposition of Copper for Enhanced Nitrate Sensing. *ChemElectroChem* **2025**, *12*, e202500013, doi:10.1002/celc.202500013.
64. Kadian, S.; Sahoo, S.S.; Kumari, P.; Shukla, S.; Narayan, R.J. Minimally Invasive Detection of Buprenorphine Using a Carbon-Coated 3D-Printed Microneedle Array. *Microchim Acta* **2024**, *191*, 672, doi:10.1007/s00604-024-06754-x.
65. Liu, P.-C.; Chou, T.-I.; Chiu, S.-W.; Tang, K.-T. A Dual-Path Deep Learning Model for Low-Cost Air Quality Sensor Calibration. *IEEE Sensors J.* **2024**, *24*, 39914–39922, doi:10.1109/JSEN.2024.3472291.
66. Siddiqui, J.; Deen, M.J. Modeling Biodegradable Free Chlorine Sensor Performance Using Artificial Neural Networks. *Adv Materials Technologies* **2024**, *9*, 2300990, doi:10.1002/admt.202300990.
67. Xing, Z.; Jiang, Y.; Zogona, D.; Wu, T.; Xu, X. Fully Nondestructive Analysis of Capsaicinoids Electrochemistry Data with Deep Neural Network Enables Portable System. *Food Chemistry* **2023**, *417*, 135882, doi:10.1016/j.foodchem.2023.135882.
68. Matsumoto, T.; Du, L.; Rodino, F.; Thoma, Y.; Premachandra, C.; Carrara, S. Optimized Quantification of Multiple Drug Concentrations by WeightedMSE With Machine Learning on Electrochemical Sensor. *IEEE Sens. Lett.* **2024**, *8*, 1–4, doi:10.1109/LSENS.2024.3452009.
69. Landolsi, K.; Jacem, W.; Hamza, K. A Hybrid GA-ANN Model to Predict the Response of Heavy Metal Ion Sensor. In Proceedings of the 2023 IEEE International Conference on Artificial Intelligence & Green Energy (ICAIGE); IEEE: Sousse, Tunisia, October 12 2023; pp. 1–5.
70. Aliev, T.A.; Belyaev, V.E.; Pomytkina, A.V.; Nesterov, P.V.; Shityakov, S.; Sadovnichii, R.V.; Novikov, A.S.; Orlova, O.Yu.; Masalovich, M.S.; Skorb, E.V. Electrochemical Sensor to Detect Antibiotics in Milk Based on Machine Learning Algorithms. *ACS Appl. Mater. Interfaces* **2023**, *15*, 52010–52020, doi:10.1021/acsami.3c12050.

71. Du, L.; Matsumoto, T.; Rodino, F.; Premachandra, C.; Thoma, Y.; Carrara, S. Optimized IdentQuantNet: A Machine Learning-Based Approach for Identification and Quantification of Multiple Drugs with Interaction on Electrochemical Sensors in Personalized Medicine. *Microchemical Journal* **2025**, *219*, 116191, doi:10.1016/j.microc.2025.116191.
72. Kammarchedu, V.; Ebrahimi, A. Advancing Electrochemical Screening of Neurotransmitters Using a Customizable Machine Learning-Based Multimodal System. *IEEE Sens. Lett.* **2023**, *7*, 1–4, doi:10.1109/LSENS.2023.3247002.
73. Yang, W.; Geng, L. Machine Learning Assisted Electrochemical Sensing Technology for the Detection of Nitrate Ions in PM2.5. *International Journal of Electrochemical Science* **2024**, *19*, 100430, doi:10.1016/j.ijoes.2023.100430.
74. Du, L.; Thoma, Y.; Rodino, F.; Carrara, S. Automatic Simulation of Electrochemical Sensors by Machine Learning for Drugs Quantification. *Electrochimica Acta* **2024**, *491*, 144304, doi:10.1016/j.electacta.2024.144304.
75. Liang, C.; Jiao, C.; Gou, H.; Luo, H.; Diao, Y.; Han, Y.; Gan, F.; Zhang, D.; Wu, X. Facile Construction of Electrochemical and Self-Powered Wearable Pressure Sensors Based on Metallic Corrosion Effects. *Nano Energy* **2022**, *104*, 107954, doi:10.1016/j.nanoen.2022.107954.
76. Apostolopoulos, I.D.; Androulakis, S.; Kalkavouras, P.; Fouskas, G.; Pandis, S.N. Calibration and Inter-Unit Consistency Assessment of an Electrochemical Sensor System Using Machine Learning. *Sensors* **2024**, *24*, 4110, doi:10.3390/s24134110.
77. De Oliveira Filho, J.I.; Faleiros, M.C.; Ferreira, D.C.; Mani, V.; Salama, K.N. Empowering Electrochemical Biosensors with AI: Overcoming Interference for Precise Dopamine Detection in Complex Samples. *Advanced Intelligent Systems* **2023**, *5*, 2300227, doi:10.1002/aisy.202300227.
78. Jung, H.H.; Yea, J.; Lee, H.; Jung, H.N.; Jekal, J.; Lee, H.; Ha, J.; Oh, S.; Song, S.; Son, J.; et al. Taste Bud-Inspired Single-Drop Multitaste Sensing for Comprehensive Flavor Analysis with Deep Learning Algorithms. *ACS Appl. Mater. Interfaces* **2023**, *15*, 46041–46053, doi:10.1021/acsami.3c09684.
79. Wang, Y.; Hui, B.; Zhang, Y.; Liu, K.; Wang, J.; Zhang, D.; Liang, J.; Li, J.; Zhang, K.; Yang, S. KNN-Synergized Dual-Mode Pd@Cu-MOF Biosensor Breaks Matrix Barriers for Ascorbic Acid Detection in Biofluids and Fruits. *Chemical Engineering Journal* **2025**, *520*, 166355, doi:10.1016/j.cej.2025.166355.
80. Sun, X.; Liu, F.; Xue, X. Identification of Liquor Adulteration Based on Machine Learning and Electrochemical Sensor. *Food Measure* **2024**, *18*, 3502–3509, doi:10.1007/s11694-024-02420-1.
81. Martins, B.R.; Andrade, C.M.R.; Simão, G.F.; De Paula Martins, R.; De Faria, L.V.; Matias, T.A.; Júnior, V.R.; Munoz, R.A.A.; Alves, R.P. Electrochemical Immunosensors on Laser-Induced Graphene Platforms for Monitoring of Anti-RBD Antibodies After SARS-CoV-2 Infection. *Biosensors* **2024**, *14*, 514, doi:10.3390/bios14110514.
82. Lee, K.; Ha, S.M.; Gurudatt, N.G.; Heo, W.; Hyun, K.-A.; Kim, J.; Jung, H.-I. Machine Learning-Powered Electrochemical Aptasensor for Simultaneous Monitoring of Di(2-Ethylhexyl) Phthalate and Bisphenol A in Variable pH Environments. *Journal of Hazardous Materials* **2024**, *462*, 132775, doi:10.1016/j.jhazmat.2023.132775.
83. Yamanouchi, M.; Yokoshiki, Y.; Dohi, M.; Tokuda, T.; Koh, S.; Watanabe, T. Machine Learning–Integrated Electrochemical Sensors for Accurate and Continuous Free Chlorine Monitoring. *ACS Sens.* **2025**, *10*, 8743–8753, doi:10.1021/acssensors.5c02634.
84. Garcia-Junior, M.A.; Andrade, B.S.; Lima, A.P.; Soares, I.P.; Notário, A.F.O.; Bernardino, S.S.; Guevara-Vega, M.F.; Honório-Silva, G.; Munoz, R.A.A.; Jardim, A.C.G.; et al. Artificial-Intelligence Bio-Inspired Peptide for Salivary Detection of SARS-CoV-2 in Electrochemical Biosensor Integrated with Machine Learning Algorithms. *Biosensors* **2025**, *15*, 75, doi:10.3390/bios15020075.
85. Emaminejad, S.A.; Sparks, J.; Cusick, R.D. Integrating Bio-Electrochemical Sensors and Machine Learning to Predict the Efficacy of Biological Nutrient Removal Processes at Water Resource Recovery Facilities. *Environ. Sci. Technol.* **2023**, *57*, 18372–18381, doi:10.1021/acs.est.3c00352.
86. Bao, Q.; Li, G.; Yang, Z.; Qu, Z.; Wei, J.; Cheng, W.; Lin, L. Multichannel Electrochemical Workstation-Based Data Collection Combined with Machine Learning for Online Analysis of Tyrosine. *New J. Chem.* **2023**, *47*, 8073–8082, doi:10.1039/D3NJ00351E.
87. Wang, M.; Cetó, X.; Del Valle, M. A Novel Electronic Tongue Using Electropolymerized Molecularly Imprinted Polymers for the Simultaneous Determination of Active Pharmaceutical Ingredients. *Biosensors and Bioelectronics* **2022**, *198*, 113807, doi:10.1016/j.bios.2021.113807.
88. Shan, A.; Imran, H.; Batool, K.; Baig, M.M.; Lee, S.G.; Lim, S. Rapid On-Site Detection of Riboflavin Using Nanozyme GdSn-MnO2 Based Printed Electrode Interface with Portable and Wireless Sensor Device. *Journal of Alloys and Compounds* **2025**, *1032*, 181189, doi:10.1016/j.jallcom.2025.181189.

89. Zhu, X.; Liu, P.; Ge, Y.; Wu, R.; Xue, T.; Sheng, Y.; Ai, S.; Tang, K.; Wen, Y. MoS2/MWCNTs Porous Nanohybrid Network with Oxidase-like Characteristic as Electrochemical Nanozyme Sensor Coupled with Machine Learning for Intelligent Analysis of Carbendazim. *Journal of Electroanalytical Chemistry* **2020**, *862*, 113940, doi:10.1016/j.jelechem.2020.113940.
90. Gürsu, G.; Yıldız, D.E.; Taşaltın, N.; Baytemir, G.; Karakuş, S.; Karaca, B.; Akarsu, C.H.; Başçeken, S. Impact of Nitro Substituents on Dopamine Sensing and Nanostructure Morphology: A Machine Learning Approach for PANI:2- and 3-Nitro-1H-Pyrrole Nanocomposite Sensors. *J. Electrochem. Soc.* **2024**, *171*, 127512, doi:10.1149/1945-7111/ad9ccb.
91. Challhua, R.; Prati, R.; Champi, A. Feature Engineering and Machine Learning for Electrochemical Detection of Rabies Virus in Graphene-Based Biosensors. *Microchemical Journal* **2024**, *204*, 111074, doi:10.1016/j.microc.2024.111074.
92. Xu, Y.; Rao, Z.; Liu, Z.; Zheng, W.; Zhou, Y.; Lu, N.; Yang, Y. Improving the Selectivity in Electrochemical Detection of Chloramphenicol Against Metronidazole With Machine Learning. *IEEE Sensors J.* **2023**, *23*, 17883–17890, doi:10.1109/JSEN.2023.3291423.
93. Datta, B.; Manasur, B.; Sreelekha, G.; Verma, P.; Adak, C.; Shukla, R.P.; Dutta, G. Quantification of L-Lactic Acid in Human Plasma Samples Using Ni-Based Electrodes and Machine Learning Approach. *Talanta* **2025**, *286*, 127493, doi:10.1016/j.talanta.2024.127493.
